# DksA inhibitors against intracellular and persistent *Salmonella* are effective in acute models of infection

**DOI:** 10.1126/sciadv.aea6832

**Published:** 2026-03-18

**Authors:** Ju-Sim Kim, Vijay Kumar, Lin Liu, Yu J. Choi, Simona Senovaityte, Bruce D. McCollister, Nathan Wlodarchak, David J. Orlicky, Peter J. Rice, Shaodong Dai, Michael F. Wempe, Andrés Vázquez-Torres

**Affiliations:** ^1^ Department of Immunology and Microbiology, University of Colorado School of Medicine, Aurora, CO, USA.; ^2^ Department of Pharmaceutical Sciences, Skaggs School of Pharmacy and Pharmaceutical Sciences, University of Colorado, Aurora, CO, USA.; ^3^ Cogent Biosciences, 4840 Pearl E. Circle, Boulder, CO, USA.; ^4^ University of Colorado Cancer Center, University of Colorado, Aurora, CO, USA.; ^5^ Department of Medicine, Division of Infectious Diseases, University of Colorado School of Medicine, Aurora, CO, USA.; ^6^ Veterans Affairs Eastern Colorado Health Care System, Denver, CO, USA.; ^7^ Department of Pathology, University of Colorado School of Medicine, Aurora, CO, USA.; ^8^ Department of Biologics and Physical Sciences, Kentucky State University, Frankfort, KY, USA.

## Abstract

We are in dire need of antibiotics endowed with new mechanisms of action. The DksA protein regulates the transcription of genes involved in metabolism, translation, and virulence in Gram-negative bacteria. DksA is evolutionarily conserved among Gram-negative pathogens but is absent in humans. Here, we identified a conserved acidic pocket at the tip of the coiled-coil domain of DksA that is amenable for drug development. Our bioinformatics and experimental approaches identified *N*-(3,4-dimethoxyphenyl)-1H-1,2,4-triazole-3-carboxamide as a DksA inhibitor with moderate antimicrobial activity. Derivatization of the dimethoxyphenyl functionality and aliphatic linker of *N*-(3,4-dimethoxyphenyl)-1H-1,2,4-triazole-3-carboxamide generated several new chemical entities with excellent IC_50_ values against DksA-regulated in vitro transcription and improved antimicrobial activity against *Salmonella* and several other Gram-negative bacteria. Our pharmacokinetic and pharmacodynamic evaluations indicate that the *N*-(4-phenylbutyl)-1H-1,2,4-triazole-3-carboxamide analog is absorbed in the gastrointestinal tract of rats and is distributed into viscera. The systemic administration of the *N*-(4-phenylbutyl)-1H-1,2,4-triazole-3-carboxamide analog protected mice against oral and systemic *Salmonella* infections while practically preventing the formation of microabscesses and necrotic foci in *Salmonella*-infected mice. Our investigations have identified a previously unknown class of antibiotics against the transcriptional regulator DksA that is endowed with antimicrobial activity against Gram-negative pathogens.

## Teaser

Antibiotics against the DksA transcription factor of Gram-negative rods protect mice from oral and systemic *Salmonella* infection.

## INTRODUCTION

The incidence of antibiotic-resistant bacteria is increasing at an accelerated rate due to prescription mismanagement, agricultural abuse, socioeconomic inequalities, and lack of novel antibiotic development (*1*). Only 24 new antibiotics have been approved since 1987 and none with novel mechanisms of action (*2*–*4*). After the initial boom of antibiotic discovery in the early 20th century, the pharmaceutical industry shifted away from antibiotic research due to low return on investment (*2*, *5*). Most newer antibiotics are simple modifications or reformulations of existing drugs (*2*), severely limiting mechanistic diversity among available antibiotics.

Because of physiological properties related to cell wall composition, efflux systems, or metabolic preferences, many populations of bacteria are intrinsically resistant to several classes of antibiotics. Bacteria have adopted multiple strategies against “natural antibiotics” secreted by microbes occupying overlapping ecological niches (*6*). Bacteria can also acquire antibiotic resistance traits from other organisms (*4*, *5*, *7*, *8*), a situation that is compounded by the blanketed utilization of antibiotics in domestic animals and their improper application in human prescriptions. β-Lactams are the most prescribed antibiotics worldwide. Hence, it is not unexpected that the acquisition of β-lactamases has proliferated among bacteria (*9*). β-Lactamase inhibitors and other classes of antibiotics are facing similar challenges (*10*, *11*). In addition to the proliferation of antimicrobial resistance due to the selective pressures associated with antibiotic usage, the evolution of antibiotic resistance has outpaced anthropogenic drug manufacturing and even preceded it. For example, *Staphylococcus aureus* acquired the methicillin resistance locus *mecA* 14 years before methicillin was clinically used (*12*). Collectively, these problems underscore the dire need for novel, mechanistically diverse antibiotics.

The Centers for Disease Control and Prevention identified 16 bacterial pathogens of public health concern, all of which are resistant to numerous classes of antibiotics (*13*). Ten of these threats are Gram-negative bacteria, including 3 of the 4 most problematic pathogens. Of the 41 antibiotics in the development pipeline, only 10 represent a new class or mechanistic target. None of the novel classes of antibiotics are directed against Gram-negative pathogens (*13*, *14*). This leaves a considerable gap in drug discovery for *Pseudomonas aeruginosa*, *Acinetobacter baumannii*, or carbapenem-resistant Enterobacteriaceae such as *Escherichia coli* or *Klebsiella pneumoniae* (*4*).

The investigations herein have exploited the uniqueness of the DksA protein, an allosteric regulator of RNA polymerase in Gram-negative bacteria. The DksA protein binds to the secondary channel of RNA polymerase (*15*). In cooperation with the guanosine tetraphosphate alarmone, binding of the α-helical DksA protein to the secondary channel of RNA polymerase clashes with the bridge helix of the β′ subunit of RNA polymerase holoprotein, controlling the initiation of transcription (*16*). The DksA protein modulates the kinetics of the open complex of multiple genes, including those encoding ribosomal RNA, tRNA, and amino acid biosynthesis (*16*–*20*). Several virulence programs are also under control of the DksA-mediated stringent response (*17*, *21*). Hence, it is not unexpected that the DksA protein is essential in the pathogenesis of Gram-negative bacteria as varied as *Borrelia burgdorferi*, enterohemorrhagic *E. coli*, *Legionella pneumophila*, *Vibrio cholerae*, *Shigella flexneri*, or *Salmonella enterica* (*22*–*26*). In addition to fostering bacterial pathogenesis, a functional DksA protein is necessary for the expression of antibiotic tolerance (*27*–*30*).

In the following investigations, we have examined the therapeutic potential of novel antibiotics against the conserved transcription factor DskA that is present in a variety of Gram-negative bacteria but absent in Gram-positive bacteria and mammalian host cells. Our research has identified a charged pocket at the tip of the DksA coiled-coil domain that is the target of novel *N*-(substituted)-1H-1,2,4-triazole-3-carboxamide analogs. The antibiotics against DksA described here are not only endowed with antibacterial activity against bacterial cells growing extracellularly in media or intracellularly in macrophages but also protect mice against the gastroenteritis and systemic complications associated with acute *Salmonella* infections.

## RESULTS

### Identification of chemicals targeted against DksA

**Fig. 1.**
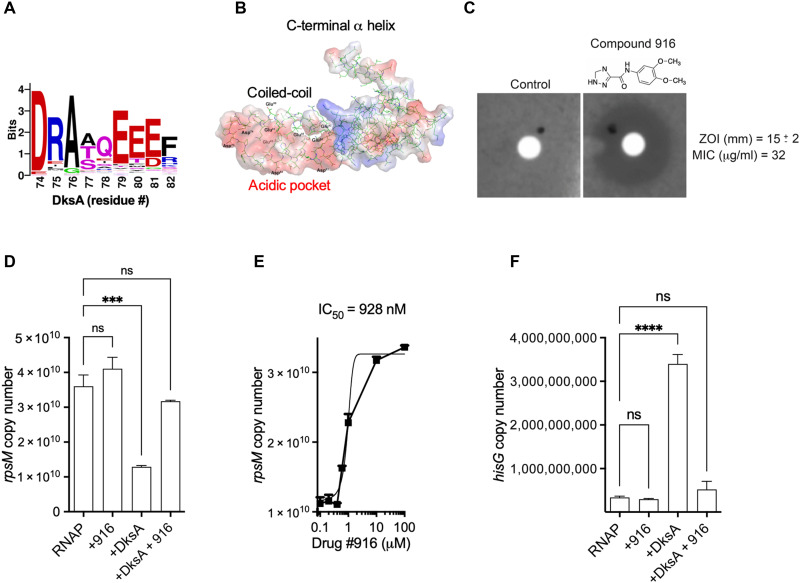
Identification of drugs with anti-DksA activity. (**A**) Graphical representation of the consensus sequence of the acidic patch at the tip of the coiled-coil domain of DksA generated using Sequence Logo (*50*) as displayed as amino acid frequency. (**B**) Surface charge of the DksA protein modeled from *E. coli* PBD 1TJL showing the acidic pocket at the tip of the coiled-coil domain in red. (**C**) Antibiotic activity of compound 916 against *Salmonella* as measured in a disk diffusion assay in agar plates prepared in EG medium. Zone of inhibition (ZOI) is measured in millimeters. The MIC was calculated in *Salmonella* grown in EG medium at 37°C in a shaker incubator. The effect of compound 916 on DksA-dependent *rpsM* (**D**) and *hisG* (**F**) in vitro transcription was assessed using quantitative reverse transcription polymerase chain reaction (qRT-PCR). Reactions were performed with 1 or 0.1 μM compound 916 for *rpsM* (D) and *hisG* (F), respectively, in the presence or absence of 5 μM DksA. RNA polymerase (RNAP) in (D) and (F) in the absence of DksA and compound 916 was tested as control. (**E**) Half maximal inhibitory concentration (IC_50_) for compound 916 was determined by fitting the data to a least square equation plotted on the semilog of the drug concentration. Data are the mean ± SD from three to four independent experiments. ns, not significant. ***P < 0.001; ****P < 0.0001.

DksA is highly conserved among Gram-negative bacteria, making this protein a potential therapeutic target. Moreover, orthologs of the DksA transcriptional regulatory protein are present in prokaryotes but absent in humans. Searching for areas in DksA potentially amenable to drug targeting, we focused on the Asp^74^ residue located at the tip of the coiled-coil domain, which is both highly conserved among DksA orthologs and is essential for DksA protein function (Fig. 1A) (*18*). Structural modeling and sequence comparison revealed an area of negatively charged residues at the tip of the coiled-coil domain that encompasses Asp^64^, Asp^71^, Asp^74^, Glu^79^, Glu^80^, Glu^81^, and Glu^85^ in the *E. coli* Protein Data Bank (PDB) 1TJL DksA structure (Fig. 1, A and B). The DksA region containing the negatively charged pocket at the tip of the coiled-coil domain was selected as a small-molecule binding target. The compounds identified in this screen were further analyzed for binding to the acidic pocket at the tip of DksA coiled-coil domain with the data-crunching computer search program Dock (*31*). We used the commercially available ZINC databases for virtual screening of compounds (*32*). This analysis identified more than 200 compounds (table S1). A panel of 40 compounds was selected from the top hits based on free energy binding scores (table S1 in yellow). These compounds were investigated using the computer modeling program O (*33*). Two additional criteria were applied to narrow down the list of candidates. First, the compounds needed to fit deep into the pocket without any clashes. Second, the compounds bound to DksA should have minimal exposed moieties. The compounds selected were further refined according to energy minimization as calculated by the CNS (Crystallographic and NMR System) program (*34*).

Twenty-five drugs that met the criteria described above were obtained from MolPort (table S2) and tested for their ability to interfere with *Salmonella* growth. Compounds 370, 677, 819, and 916 exhibited inhibitory activity against *Salmonella* grown in M9 minimal media as measured by zones of inhibition in disk diffusion assays and minimal inhibitory concentrations (MICs) (Fig. 1C and fig. S1, A and B). The MIC of compound 916 [*N*-(3,4-dimethoxyphenyl)-1H-1,2,4-triazole-3-carboxamide] against nontyphoidal *S. enterica* serovar Typhimurium strain 14028s was estimated to be 32 μg/ml (i.e., 129 μM) (Fig. 1C). A similar approach also showed that compound 916 also had an MIC of 32 μg/ml against *E. coli* strain W3110. None of the other 21 compounds showed any anti-*Salmonella* activity as assessed by disk diffusion assays.

We also tested direct anti-DksA function of the 25 drugs in a high-throughput screen that combines in vitro transcription with highly specific and quantitative reverse transcription polymerase chain reaction (qRT-PCR) (*35*). DksA regulates the kinetics of the RNA polymerase–DNA open complex, repressing promoters such as *rpsM*, which encodes the 30*S* ribosomal protein S13, while activating amino acid biosynthesis and acquisition genes such as *hisG* (*19*). For our initial screen, we selected the *rpsM* promoter because it is one of the strongest promoters in the bacterial cell. From the 25 compounds tested, only compound 916 prevented DksA-mediated repression of *rpsM* in vitro transcription (Fig. 1D and fig. S1, C to F). The estimated half maximal inhibitory concentration (IC_50_) value of compound 916, as measured by the DksA-mediated repression of *rpsM* in vitro transcription, was about 928 nM (Fig. 1E). Compound 916 also antagonized DksA-mediated activation of *hisG* in vitro transcription (Fig. 1F). The opposite effects that compound 916 has on transcription of *rpsM* and *hisG* nicely mirror the DksA-mediated activation or repression of *rpsM* and *hisG* genes, respectively (*19*). We noted that as little as 0.10 μM, compound 916 completely inhibited DksA-dependent activation of *hisG* in vitro transcription, whereas up to 1.0 μM concentrations of compound 916 were required to achieve complete derepression of the *rpsM* gene. The notable differences that compound 916 has on DksA-dependent *rpsM* or *hisG* gene transcription likely reflect differences in promoter activity [in our experience, *rpsM* is driven by one of the strongest promoters in *Salmonella* (*36*)]. The opposite effects compound 916 has on *rpsM* and *hisG* expression suggest that the effects compound 916 has on transcription are mediated through DksA, not RNA polymerase. In support of this idea, addition of up to 10 μM compound 916 affected neither *rpsM* nor *hisG* in vitro transcription by the RNA polymerase itself (Fig. 1, D and F). Collectively, our investigations show high specificity of compound 916 against DksA-dependent regulation of transcription and raise the possibility that DksA inhibitors could be incorporated in the treatment of infections caused by Gram-negative bacteria.

### Compound 916 inhibits virulence gene transcription of intracellular *Salmonella*

**Fig. 2.**
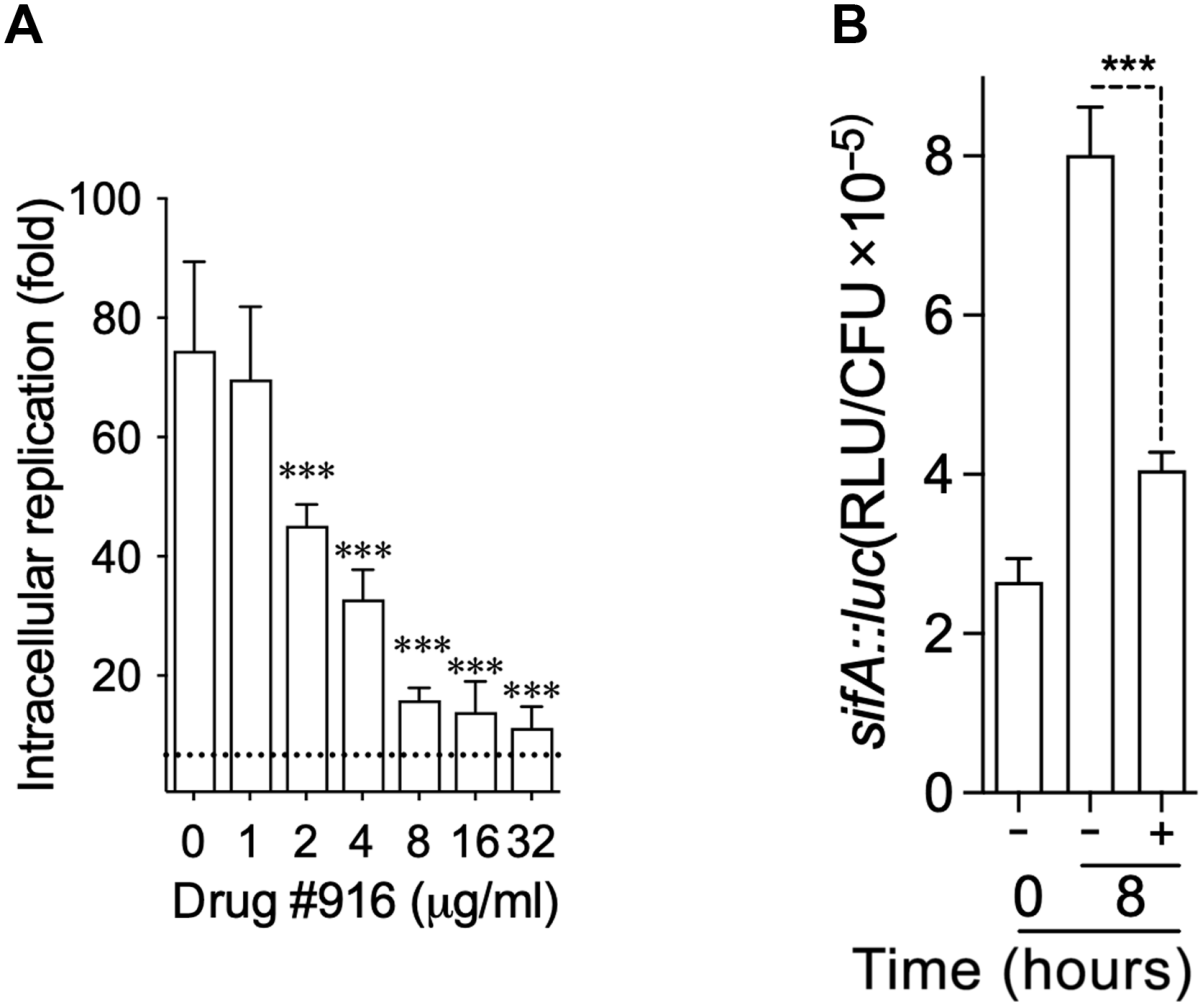
Antimicrobial activity of anti-DksA drug against intracellular *Salmonella*. (**A**) Intracellular replication of the indicated *Salmonella* strains 20 hours after infection of J774 cells with an MOI of 2. Where indicated, J774 cells were treated with compound 916. ***P < 0.001 as determined by one-way analysis of variance (ANOVA). (**B**) Intracellular SPI-2 gene transcription was assessed measure luminescence signal from To and T8 treated with drug #916 (16 μg/ml) or without. Data are the mean ± SD from two independent experiments, *P* < 0.001 by one-way ANOVA.

Compound 916 inhibits DksA-mediated function in the reconstituted biochemical in vitro transcription system and has antimicrobial activity against *E. coli* and *Salmonella* grown in minimal media. We evaluated the antibiotic activity of compound 916 against *Salmonella* residing within murine host cells. We found that compound 916 exerts antimicrobial activity against intracellular *Salmonella* growing in macrophages in complex cell culture media containing 10% fetal bovine serum (Fig. 2A). These findings suggest reasonable penetrability of compound 916 through cell host membranes. The addition of compound 916 (32 μg/ml) did not change (*P* = 0.3) the low levels of lactate dehydrogenase released from J774 cells 18 hours after infection with wild-type or Δ*dksA Salmonella* (fig. S2A), indicating that this drug appears to be well tolerated by host cells.

The poor intracellular growth of Δ*dksA Salmonella* was unaffected by compound 916, suggesting that this drug has minimal off-target effects on bacteria. In addition to coordinating the expression of critical metabolic biosynthetic pathways, DksA is a critical activator of the *Salmonella* pathogenicity island-2 (SPI-2) virulence program (*21*, *37*, *38*) encoding a type III secretion system essential for the pathogenesis of this enteric bacterium (*39*). The addition of compound 916 to *Salmonella*-infected macrophages repressed transcription of the *sifA* gene encoding a key effector of the SPI2 type III secretion system (Fig. 2B). Cumulatively, these investigations demonstrate that compound 916 inhibits transcription of DksA-dependent housekeeping genes and the *sifA* virulence gene.

### Identification of derivatives of *N*-(3,4-dimethoxyphenyl)-1H-1,2,4-triazole-3-carboxamide (compound 916) with improved anti-DksA activity

**Fig. 3.**
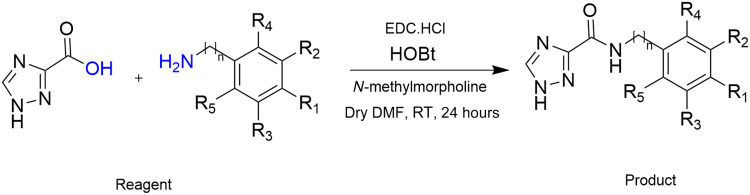
Strategy used for chemical synthesis of anti-DksA analogs. General approach to synthesized anti-DksA analogs in which the 1,2,4-triazole-3-carboxyamide functional group was subject to systematic medicinal chemistry. RT, room temperature.

We used in silico modeling to inform SAR studies of compound 916 and DksA. Our analyses indicate that there is space to diversify the compound 916 scaffold. We initiated our search for improved DksA inhibitors by maintaining the 1,2,4-triazole-3-carboxyamide functional group while introducing modifications in the 3,4-dimethoxyphenyl group (Fig. 3). The compounds were prepared via the coupling of a carboxylic acid with an amine. We prioritized production of analogs according to the energy minimizations calculated with the CNS program (*34*). On the basis of free energy binding scores and molecular complementarity, we identified derivatives of compound 916 with greater docking scores toward the pocket at the tip of the DksA coiled-coil domain. We introduced 3,4-dimethoxybenzyl and 3,4-dimethoxyphenethyl substitutions to establish whether one or more methylene groups between the 3,4-dimethoxyphenyl functionality improves activity. We also replaced the 3,4-dimethoxyphenyl functionality with aromatic groups and produced new chemical entities based on the scaffold of compound 916 (table S3).

**Fig. 4.**
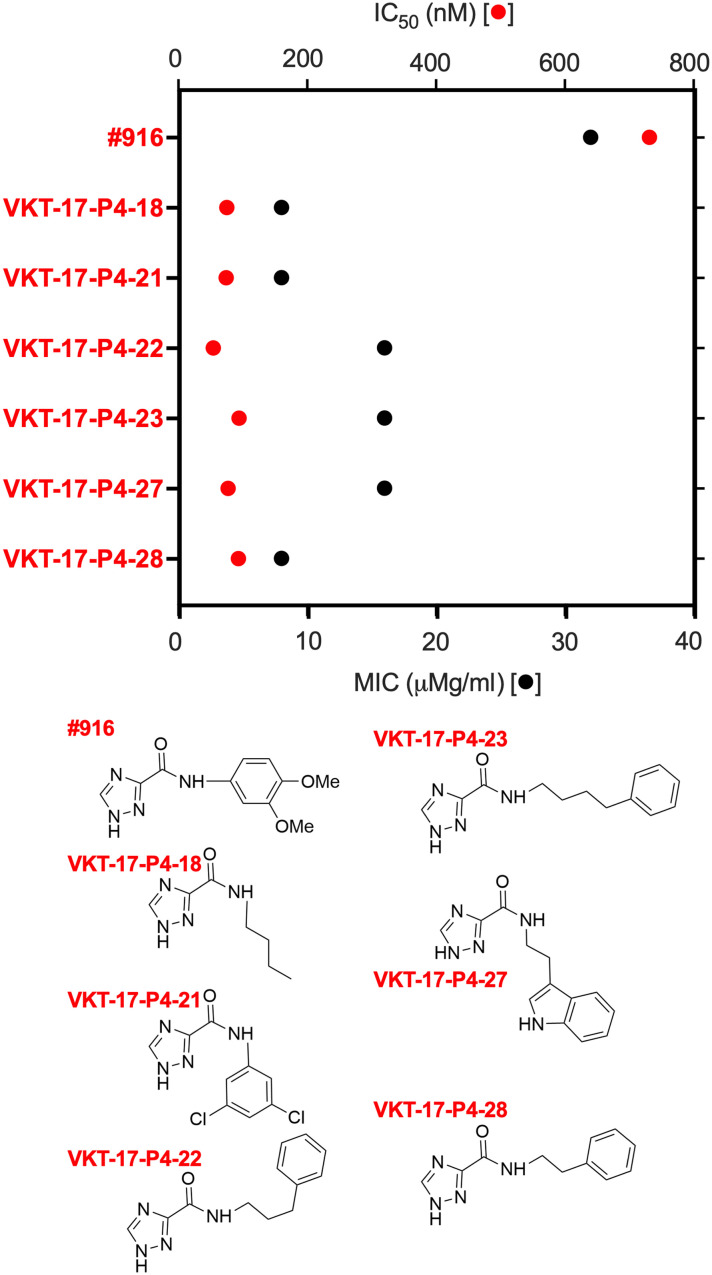
Screen of *N*-(substituted)-1H-1,2,4-triazole-3-carboxamide analogs with improved anti-DksA activity. The bottom *x* axis with symbols in black shows MIC values for the analogs derived from the parent compound #916. The top *x* axis depicts the IC_50_ inhibition of in vitro–transcribed DNA encoding the *rpsM* promoter in reactions containing recombinant DksA protein and the RNA polymerase holoenzyme. The bottom of the figure shows the structures of the anti-DksA analogs.

All synthetically prepared compounds were purified (e.g., column chromatography) and structurally characterized via analytical methods, such as 400-MHz ^1^H–nuclear magnetic resonance (NMR) and 100-MHz ^13^C-NMR, and the mass was confirmed via liquid chromatography–tandem mass spectrometry (LC-MS/MS) (fig. S3). Six of the “916 analog” derivatives showed improved MIC values (i.e., 8 to 16 μg/ml) and greater inhibition of DksA function as assessed in our high-throughput *livJ* in vitro transcription assay (i.e., inhibition of transcription at <0.1 μM compared to 1 mM for the parent 916 compound; see table S3). The new DksA inhibitors also had improved IC_50_ values as determined by the derepression of *rpsM* in vitro transcription in reactions containing RNA polymerase and DksA (Fig. 4). The IC_50_ values for the derivatives were up to 10-fold lower than those calculated for the parent drug (e.g., 72 versus 928 nM, respectively). We also confirmed direct interaction between one derivative, VKT-17-P4-23, by using microscale thermophoresis (MST) with a dissociation constant (*K*_d_) of 124 μM (fig. S4C). Because GreA and GreB factors share similar coiled-coil structure with DksA (fig. S4A) but lack the acidic pocket of DksA (fig. S4B), we also tested binding of VKT-17-P4-23 to the transcription elongation and fidelity GreA and GreB recombinant proteins. Binding assessment by MST strongly suggests that GreA and GreB do not bind to VKT-17-P4-23 (fig. S4C). Together, our investigations have identified analogs of *N*-(3,4-dimethoxyphenyl)-1H-1,2,4-triazole-3-carboxamide with improved anti-DksA activity and specific binding to DksA.

### *N*-(3,4-dimethoxyphenyl)-1H-1,2,4-triazole-3-carboxamide analogs exhibit antimicrobial activity against multiple Gram-negative pathogens and intracellular *Salmonella*

**Fig. 5.**
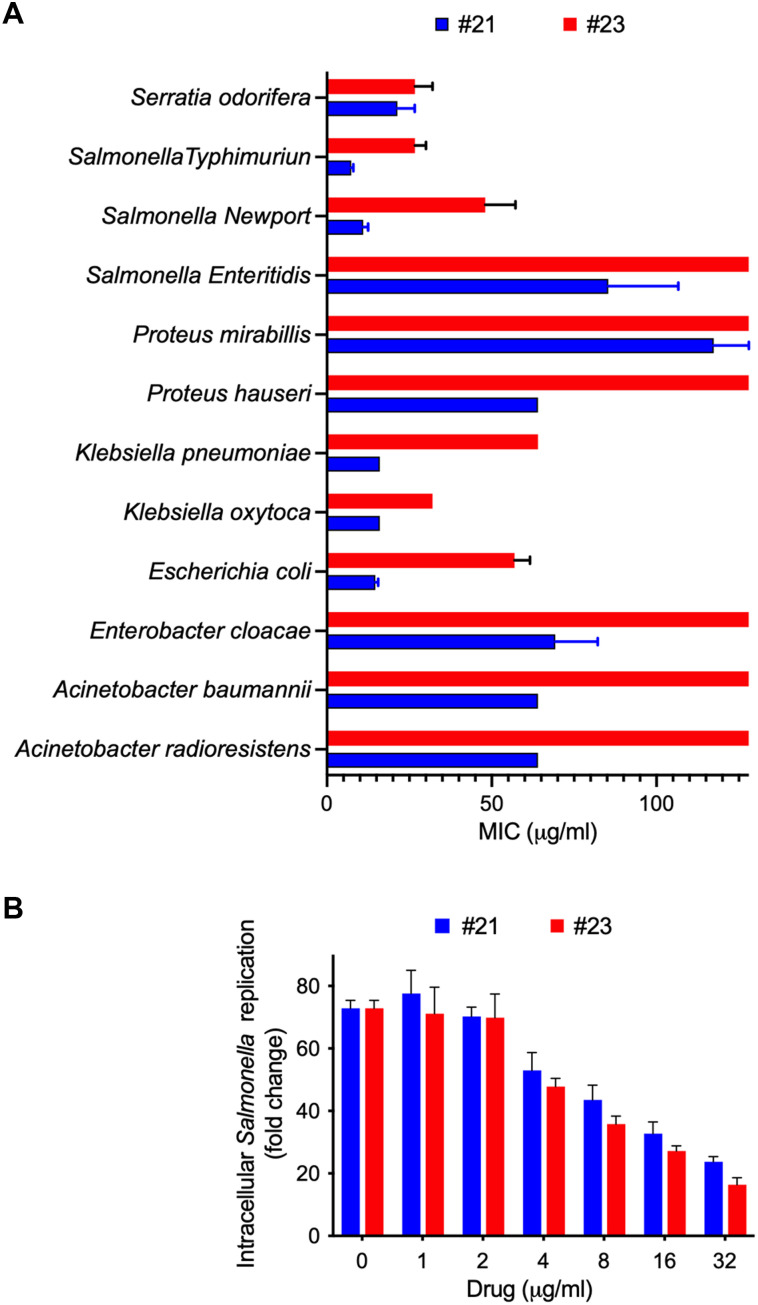
Effectiveness of *N*-(3,4-dimethoxyphenyl)-1H-1,2,4-triazole-3-carboxamide analogs against several Gram-negative pathogens and intracellular *Salmonella*. (**A**) The MIC of compounds VKT-17-P4-21 (#21) and VKT-17-P4-23 (#23) was determined against several Gram-negative pathogens isolated at the Microbiology Clinical Laboratory at the University of Colorado Hospital. (**B**) Antimicrobial activity of anti-DksA drugs against intracellular *Salmonella* after 20 hours of culture in J774 cells. Data are the mean ± SD from three to four independent experiments. Four micrograms per milliliter is the minimal concentration of VKT-17-P4-21 and VKT-17-P4-23 at which there is statistical significance (*P* < 0.0001 by two-way ANOVA) when compared to untreated controls.

*N*-(3,5-dichlorophenyl)-1H-1,2,4-triazole-3-carboxamide (i.e., VKT-17-P4-21) exhibited substantial antimicrobial activity against all pathogens tested (MIC values ranged from 8 to 128 μg/ml against *S.* Typhimurium and *Proteus mirabilis*, respectively) (Fig. 5A and table S4). The VKT-17-P4-21 analog also showed antimicrobial activity (i.e., MIC = 64 μg/ml) against antibiotic-resistant bacteria such as *A. baumannii*. In general, the *N*-(4-phenylbutyl)-1H-1,2,4-triazole-3-carboxamide analog (i.e., VKT-17-P4-23) showed more limited antimicrobial activity than compound VKT-17-P4-21. The estimated MIC for VKT-17-P4-23 against *S.* Typhimurium was 16 μg/ml. We also compared the effectiveness of VKT-17-P4-21 and VKT-17-P4-23 against intracellular *Salmonella* residing in J774 macrophage-like cells (Fig. 5B). VKT-17-P4-21 and VKT-17-P4-23 inhibited growth of intracellular *Salmonella* in a dose-dependent manner with similar effectiveness (*P* = 0.698). This finding is unexpected since the MIC against *Salmonella* for VKT-17-P4-21 is lower than VKT-17-P4-23.

### *N*-(3,4-dimethoxyphenyl)-1H-1,2,4-triazole-3-carboxamide analogs have antimicrobial activity against persistent *Salmonella*

**Fig. 6.**
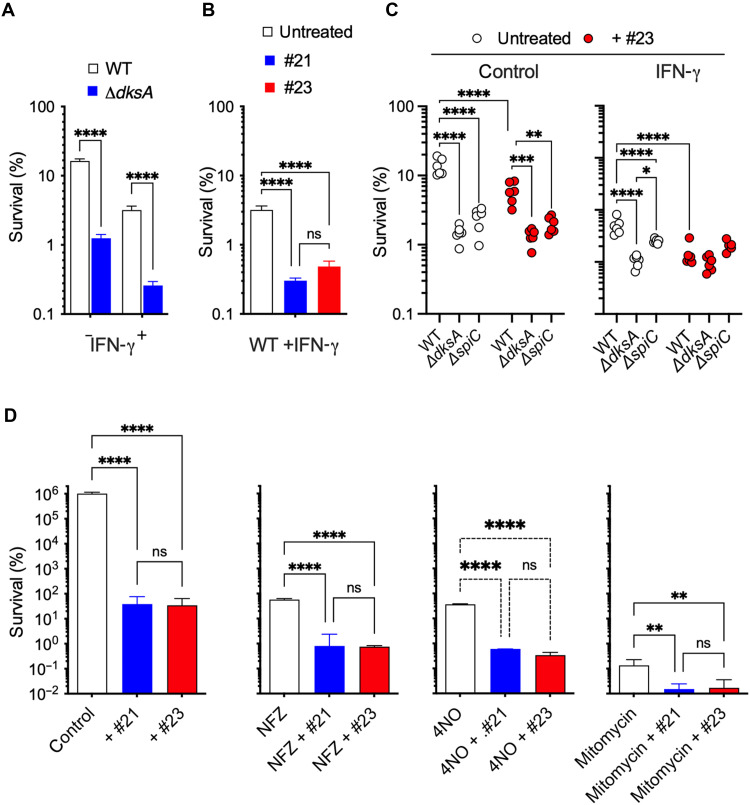
*N*-(3,4-dimethoxyphenyl)-1H-1,2,4-triazole-3-carboxamide analogs exert antimicrobial activity against persistent *Salmonella*. (**A**) Survival of wild-type (WT) and Δ*dksA Salmonella* in periodate-elicited macrophages from C57BL/6 mice. Where indicated, macrophages were treated with IFN-γ (200 U/ml) for 24 hours before infection with the indicated *Salmonella* strains at an MOI of 10. % survival = (CFU at *t*20h/CFU *t*0h) × 100. *N* = 4. (**B** and **C**) Effect of anti-DksA drugs against persistent populations of intracellular *Salmonella* selected in IFN-γ–treated periodate-elicited macrophages from C57BL/6 mice as described in the “Antimicrobial activity against intracellular persisters” section of the Materials and Methods. Where indicated, the macrophages were infected with wild-type, Δ*dksA* or Δ*spiC Salmonella* at an MOI of 2. Untreated macrophages were used in (C) as controls of IFN-γ–treated cells. Where indicated, the specimens were treated with the anti-DksA antibiotic VKT-17-P4-21 (i.e., #21) or VKT-17-P4-23 (i.e., #23) (32 μg/ml). *N* = 4 to 6. **P < 0.01; ****P < 0.0001 by one- or two-way ANOVA. (**D**) Stationary phase *Salmonella* grown in LB broth were exposed to 8, 8, and 1.5 μg/ml of the DNA synthesis inhibitors nitrofurazone (NFZ), 4-nitroquinoline *N*-oxide (4NO), and mitomycin, respectively. *Salmonella* surviving 4NFZ, 4NO, and mitomycin in vitro treatment are considered persister cells. Where indicated, some of the samples were cotreated with anti-DksA compound VKT-17-P4-21 or VKT-17-P4-23 (32 μg/ml). The percentage of *Salmonella* surviving 20 hours after treatment was estimated by the number of bacteria able to form a colony in LB agar plates. *N* = 4 to 6. **P < 0.01; ****P < 0.0001 by one-way ANOVA.

Bacteria unable to synthesize the nucleoside alarmone guanosine tetraphosphate cannot establish persistent infections (*40*, *41*). Because the stringent response in Gram-negative bacteria is coordinated by the synergistic actions of guanosine tetraphosphate and DksA, we tested whether DksA facilitates the establishment of persistence by intracellular *Salmonella* in periodate-elicited macrophages from C57BL/6 mice. Interferon-γ (IFN-γ)–treated macrophages killed more than 95% of wild-type *Salmonella*; however, the remaining 5% of the cells survived in a persistent state that has been reported to be driven by the stringent response regulator guanosine tetraphosphate (*40*). *dksA*-deficient *Salmonella* was more easily killed than wild-type controls in unstimulated macrophages (Fig. 6A). We also noted that fewer Δ*dksA Salmonella* established a persistent state in IFN-γ–activated macrophages compared to wild-type controls (Fig. 6A), demonstrating a critical role for DksA for sustaining a persistent state in intracellular bacteria. Given these exciting results, we tested whether inhibitors of DksA prevent the formation of persistently infected, IFN-γ–treated macrophages. On the basis of the results presented in Fig. 5B, analogs VKT-17-P4-21 and VKT-17-P4-23 were used at 32 μg/ml. Our investigations indicate that the DksA inhibitors VKT-17-P4-21 and VKT-17-P4-23 significantly (*P* < 0.0001) reduced the formation of intracellularly selected populations of *Salmonella* persistent cells (Fig. 6B). These findings raise the interesting possibility that compounds that inhibit DksA function can be used for treatment of persistent infections as monotherapy.

Next, we examined whether the antimicrobial activity of compound VKT-17-P4-23 against DksA-mediated persistence is codependent on the SPI-2 type III secretion system. For these experiments, we used a Δ*spiC* mutant deficient in a critical component of the SPI-2 translocon. The Δ*dksA* and Δ*spiC* mutants were similarly attenuated in periodate-elicited macrophages (Fig. 6C). Addition of VKT-17-P4-23 increased killing of wild-type *Salmonella* but not Δ*dksA* and Δ*spiC*. These findings suggest that compound #23 inhibits the DksA-regulated, SPI-2-dependent survival of *Salmonella* during the innate response of macrophages. In this population of macrophages, most benefits of DksA are dependent on the SPI-2 type III secretion system. We also checked the antimicrobial activity of VKT-17-P4-23 persistent *Salmonella* selected in macrophages by the stimulation with IFN-γ (*40*, *42*, *43*). Wild-type *Salmonella* was isolated in lower numbers from IFN-γ–treated macrophages than untreated controls (Fig. 6C). The addition of VKT-17-P4-23 to IFN-γ–treated macrophages reduced the *Salmonella* burden compared to untreated controls. This observation is consistent with the idea that VKT-17-P4-23 is active against persister *Salmonella*. The antimicrobial activity of VKT-17-P4-23 against persister *Salmonella* was dependent on DksA as suggested by the fact that Δ*dksA Salmonella* was isolated in fewer numbers from IFN-γ–treated macrophages than wild-type isogenic controls and that VKT-17-P4-23 did not worsen the hypersusceptibility of Δ*dksA Salmonella* in IFN-γ–treated macrophages. We also observed that Δ*dksA Salmonella* was isolated in lower numbers from IFN-γ–treated macrophages than Δ*spiC* controls, suggesting that DksA contributes to the establishment of persistence in ways that are independent of the SPI-2 type III secretion system.

The stringent response coordinates a genetic program that results in the development of persistent populations of bacteria that are resistant to antibiotics. We next tested the potential of our lead compounds to promote killing of persistent populations of *Salmonella* selected in response to antibiotics that target DNA biosynthesis. Compounds VKT-17-P4-21 and VKT-17-P4-23 significantly (*P* < 0.01) reduced the number of *Salmonella* persistently infected cells selected after treatment with 4-nitroquinoline *N*-oxide (4NO), nitrofurazone (NFZ), or mitomycin (Fig. 6D). Our research demonstrates that inhibitors of DksA sensitize persistent bacteria to antibiotic killing. This information indicates that, in addition to serving as direct antibiotics, compounds against DksA may be used in combination therapies.

### *N*-(3,4-dimethoxyphenyl)-1H-1,2,4-triazole-3-carboxamide analogs are absorbed in rats

Because of their inhibitory effects against DksA-mediated transcription and because of their antimicrobial activity against bacterial cell cultures and intracellular *Salmonella*, we were interested in evaluating the effectiveness of anti-DksA compounds in an experimental model of *Salmonella* infection. In preparation for the evaluation of antibiotic activity in a murine model of *Salmonella* infection, we obtained pharmacokinetic (PK) information on the absorption and distribution of the antibiotic candidates VKT-17-P4-21 (fig. S5A) and VKT-17-P4-23 (fig. S5B) in healthy male Sprague-Dawley rats. The area under the curve (AUC)_0-24h_ for VKT-17-P4-21 after intraperitoneal administration was 254.1 ng*hour/ml, whereas the oral AUC_0-24h_ was 821.3 ng*hour/ml with a *C*_max_ of 183 ng/ml and a *T*_max_ of 6.0 hours. In contrast, analog VKT-17-P4-23 displayed an intraperitoneal (1.0 mg/kg) AUC_0-24h_ of 6979 ng*hour/ml with a *C*_max_ of 798 ng/ml and a *T*_max_ of 2.0 hours. The analog VKT-17-P4-23 displayed an extended absorption period leading to significantly greater exposure. When VKT-17-P4-23 was administered orally (10.0 mg/kg), the AUC_0-24h_ was 1271 ng*hour/ml with a *C*_max_ of 447 ng/ml and a *T*_max_ of 0.5 hours. Thus, when one compares compound VKT-17-P4-21 to VKT-17-P4-23, the VKT-17-P4-23 analog had more favorable PK properties.

**Fig. 7.**
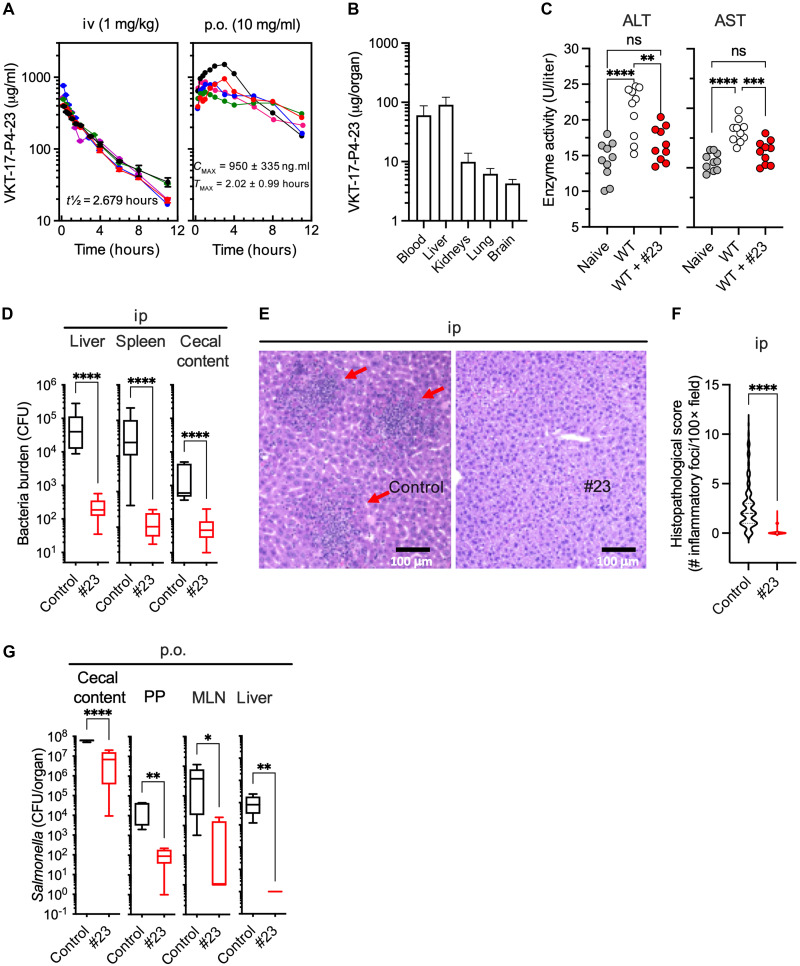
Compound VKT-17-P4-23 shows antibiotic activity in murine models of systemic and oral *Salmonella* infection. (**A**) PK characterization of anti-DksA drugs in fasted, male Sprague-Dawley rats dosed with 10 mg/kg p.o. or 1.0 mg/kg intraperitoneal (ip) of compounds VKT-17-P4-21 (i.e., #21) and VKT-17-P4-23 (i.e., #23). The concentrations of drug in blood (A) were quantified by LC-MS/MS. iv, intravenous. (**B**) PD analysis in tissues of rats inoculated with 10 mg/kg p.o. of VKT-17-P4-23. *N* = 4. (**C**) Concentrations of liver enzymes in plasma of C57BL/6 mice infected with about 200 CFU of *Salmonella* for 2 days. Some of the mice were treated with drug #23 (10 mg/kg) twice a day for 2 days, starting 12 hours before infection. Controls were inoculated with the PEG400 vehicle. Uninfected mice served to determine baseline levels of ALT and AST in blood. *N* = 5. **P < 0.01, ***P < 0.001, and ****P < 0.0001, as determined by one-way ANOVA. (**D**) C57BL/6 mice were inoculated intraperitoneally with about 200 CFU of wild-type *Salmonella*. Some mice were treated with drug #23 as described above. *Salmonella* burden in viscera and cecal content was calculated after 48 hours of infection. *N* = 10 (5 males, 5 females). (**E**) Hematoxylin and eosin (H&E)–stained sections were prepared from paraffin-embedded liver tissues from mice in (D). Images are representative from 10 mice per group. (**F**) Quantification of inflammatory foci in H&E-stained livers from data in (E). ****P < 0.0001 as determined by two-tailed *t* test. (**G**) Effects of VKT-17-P4-23 (10 mg/kg) treatment twice a day for 2 days on streptomycin-treated C57BL/6 mice orally gavaged with *S.* Typhimurium. The mice received drug 12 hours before *Salmonella* infection. Control mice were inoculated with the PEG400 vehicle control. *Salmonella* burdens in liver, spleen, mesenteric lymph nodes (MLN), Peyer’s patches (PP), and cecal content were determined 48 hours after infection. *N* = 10.

Next, we sought to perform a statistically more complete experiment on compound VKT-17-P4-23. First, we dosed fasted male Sprague-Dawley rats via intravenous dosing at 1.0 mg/kg, and 12 blood samples were collected. The semi-log plot of the blood concentration versus time demonstrates very consistent data with a distribution phase of essentially 24 min with a half-life (*T*_1/2_) of 2.68 hours (Fig. 7A). Second, oral gavage experiments were performed at 10.0 mg/kg. The semi-log plot of the blood concentration versus time for the oral dosing is plotted to demonstrate a very long absorption phase with extended drug concentrations with time (Fig. 7A). Third, a pharmacodynamic (PD) study was conducted by determining the concentration of VKT-17-P4-23 in blood, whole liver, both kidneys, lung, and brain after 1.0 hour of oral inoculation of rats at 10.0 mg/kg. PD assessment showed that compound VKT-17-P4-23 concentrated the highest in blood and liver, demonstrating that the liver has high hepatic extraction (Fig. 7B). Compound VKT-17-P4-23 was also observed in decreasing order in kidneys, lung, and brain. These data illustrate that compound VKT-17-P4-23 was able to cross the blood-brain barrier (BBB) and be detected in the brain samples. We also analyzed liver toxicity by assessing aspartate aminotransferase (AST) and alanine aminotransferase (ALT) in plasma. Intraperitoneal inoculation of C57BL/6 mice with *S.* Typhimurium strain 14028s increased AST and ALT plasma levels 2 days after infection (Fig. 7C). Treatment of infected mice with VKT-17-P4-23 brought AST and ALT to basal levels recorded in uninfected, naïve mice (Fig. 7C). These findings indicate that compound VKT-17-P4-23 protects mice from the liver toxicity associated with *Salmonella* infection.

### Compound VKT-17-P4-23 exhibits antibiotic activity against *Salmonella* in gastrointestinal and systemic murine models of infection

MIC values against *Salmonella* and other Gram-negative bacteria and IC_50_ estimations against DksA-mediated repression of *rpsM* transcription indicate that VKT-17-P4-23 is a less potent anti-DksA drug than its VKT-17-P4-21 analog. However, the antimicrobial activity of VKT-17-P4-21 and VKT-17-P4-23 is comparable when tested against intracellular *Salmonella* (Figs. 5B and 6A). Moreover, PK determinations indicate better absorption and distribution of compound VKT-17-P4-23 than VKT-17-P4-21. Considering these findings, we chose compound VKT-17-P4-23 for antibiotic testing in a murine model of acute *Salmonella* infection. Mice were infected with 200 to 320 colony-forming units (CFU) of wild-type *Salmonella* and treated with VKT-17-P4-23 (10 mg/kg) at 12-hour intervals. Control mice were treated in a similar fashion with the vehicle control polyethylene glycol 400 (PEG400). After 48 hours of infection, drug concentrations were determined in livers, spleens, and cecal contents (fig. S5C). Mice treated with drug VKT-17-P4-23 harbored significantly (*P* < 0.001) fewer *Salmonella* in livers, spleens, and intestinal contents than control mice treated with vehicle control (Fig. 7D). *Salmonella*-infected mice treated with VKT-17-P4-23 did not develop measurable histopathological lesions (Fig. 7, E and F). Moreover, the oral administration of VKT-17-P4-23 also protected mice against the gastrointestinal phase of the *Salmonella* infection (Fig. 7G). These findings illustrate the therapeutic potential of DksA inhibitors.

## DISCUSSION

Here, we have found small-molecule inhibitors that disrupt the action of a key metabolic- and virulence-mediating transcription factor. Our anti-DksA antibiotics have efficacy against in vitro and in vivo bacterial growth with good pharmacological properties. This is a promising first step and proof of concept for a previsusly unknown class of antibiotics. DksA is a transcription factor that is conserved in Gram-negative bacteria but absent in Gram-positive bacteria and mammalian cells. DksA has several conserved acidic amino acids on its coiled-coil region which are likely required for binding to RNA polymerase (*16*), making them an excellent target for disrupting DksA binding. This interaction is critical to control expression of several DksA-mediated virulence factors elicited by the stringent response (*18*, *26*, *35*, *44*–*48*). Phenotypic screening for biochemical and microbiologic activity would be difficult to perform in a high-throughput manner for transcriptional inhibition of DksA. Therefore, we used computational docking to search for small-molecule ligands which would bind to DksA in this region and likely disrupt transcription. We identified more than 400 compounds which may have bound DksA, and of the 25 tested, we found 4 with microbiologic activity. Compound 916 prevented DksA-mediated repression of *rpsM* in an in vitro transcription assay while repressing DksA-mediated activation of *hisG* in vitro transcription. Compound 916 did not affect transcription in the absence of DksA. Together, these findings suggest that *N*-(3,4-dimethoxyphenyl)-1H-1,2,4-triazole-3-carboxamide analogs affect transcription through their binding to DksA and not RNA polymerase, suggesting specific interactions with this transcription factor. This idea is further supported by the detection of direct binding of a derivative compound, VKT-17-P4-23, to DksA in the biophysical MST assay.

Since we had a promising hit with antimicrobial inhibition and a likely mechanism of activity, we set to improve the compound through computationally guided medicinal chemistry. The computational studies suggested that the 1,2,4-triazole-3-carboxyamide functional group made favorable hydrogen bonding contacts to the negatively charged amino acids in the pocket. Therefore, we chose to vary the 3,4-dimethoxyphenyl group. We input a variety of possible compounds designed with standard medicinal chemistry approaches and selected those with improved docking scores for synthesis. New compounds with a diverse mixture of aliphatic and aromatic functional groups were synthesized. Six of these compounds had improved microbiologic activity of two- to fourfold. Their ability to derepress *rpsM* transcription was generally improved by a factor of 10. This would suggest that improvements in microbiologic activity could be made with biophysical improvements. Of the improved compounds, compound VKT-17-P4-21 is the most analogous to the original hit, 916, with chloride substitutions for the O-methyl moieties and a para➔ortho translation. It is difficult for docking to discern such subtle differences; however, since the improvement was both biophysical and microbiologic, it is likely that stronger binding is occurring rather than just changes in bacterial entry/stability. The other active compounds shared a similarity in having an aliphatic tail or an aromatic group connected by an aliphatic tail. The length of the linker does not seem to modulate the effects too greatly, nor does substitution of the phenyl for an indole group. There are few aromatic amino acids or methionine in the hypothesized binding region on DksA, and the computationally predicted positions do not place the aromatic ring near any, suggesting that pi-pi stacking interactions with the phenyl are not a substantiative mediator of binding. Hence, this region is likely to make only weak hydrophobic interactions or none at all. This lack of “lock and key” specificity would suggest that further improvement can be gained by modifications of this region for a more robust fit.

Compounds VKT-17-P4-21 and VKT-17-P4-23 were selected to move forward since they were structurally dissimilar and had empirically better solubility and a reasonable synthetic yield. Compound VKT-17-P4-21 had some microbiologic inhibition in 12 different Gram-negative pathogens tested, whereas compound VKT-17-P4-23 only had activity in six of the same pathogens. Given the high conservation of DksA, this may be more due to bacterial entry/efflux, and notably, compound VKT-17-P4-21 has a more “compact” structure than compound VKT-17-P4-23, which has a four-carbon linker between functional groups and thus increased degrees of freedom. However, both compounds have similar MICs in an intracellular clearance assay and similar effectiveness in reducing intracellular persisters. This may be due to stability or accumulation differences. We did find that compound VKT-17-P4-23 has an extended absorption period in rats compared to compound VKT-17-P4-21 which may also suggest more biologic stability. Compound VKT-17-P4-23 has a reasonable *T*_1/2_ of 2.68 hours, which allows it to get to high concentrations in the liver and cecum. Moreover, compound VKT-17-P4-23 is even detectable in the brain, indicating that it can cross the BBB. This stability and good PK/PD properties suggest that it may be an effective therapeutic against Gram-negative pathogens and may be used against pathogens that cause infections of the central nervous system. Therefore, we tested compound VKT-17-P4-23 in a murine model of *Salmonella* infection and found that it significantly reduced systemic bacterial burden in the liver and spleen and was effective at clearing infection from the cecum, suggesting that all modes of infection are treatable with this compound. Compound VKT-17-P4-23 appears to be safe as it lowers the elevated concentrations of ALT and AST liver enzymes recorded in *Salmonella*-infected mice.

Collectively, we have identified DksA as a promising drug target in Gram-negative bacteria and found small-molecule inhibitors that disrupt its ability to regulate transcription via its interaction with RNA polymerase. After a round of modification, small molecules had improved affinity for DksA and improved microbiologic activity. These compounds inhibited the growth of a diverse panel of Gram-negative pathogens and were effective against *Salmonella* in an intracellular model of infection, including a significant reduction in the persistent population. Compound VKT-17-P4-23 was effective at clearing *Salmonella* in a mouse model of infection and demonstrated reasonable PK/PD properties. This work identifies previously unknown small molecules against a conserve targets and demonstrates their potential as safe in vivo therapeutics.

## MATERIALS AND METHODS

### Bacterial strains

The studies made use of *S. enterica* serovar Typhimurium American Type Culture Collection (ATCC) strain 14028s and its Δ*dksA::Cm* mutant derivative. All other clinical isolates are listed in table S5. Bacterial cells were grown on agar plates or Luria-Bertani (LB) broth in an orbital shaker incubator at 37°C.

### Bioinformatics

Protein sequences of the DksA coiled-coil domains were aligned using the ClustalW algorithm, and phylogenetic tree analysis was done in Dsgene (Discovery Studio). A highly conserved area of negatively charged residues in the coiled-coil domain was identified. The structure coordinates of the coiled-coil domain in *E. coli* DksA (PDB 1TJL), GreA (PDB 1GRJ), GreB (PDB 2P4V), and other Gram-negative bacteria (PDB 4IJJ and 6PTG) were retrieved from the PDB database. PDB files were loaded onto Coot (*49*) or PyMOL (version 0.99rc6 Schrödinger) programs to analyze the domain structures and amino acid interactions. The structure-based sequence alignment was performed in PyMOL.

### Chemicals

All commercial compounds used in the antibiotic screens were purchased from Molport (Lāčplēša, Rīga, Latvia). Sodium sulfate (Na_2_SO_4_), ammonium acetate (NH_4_OAC), and formic acid were purchased from Thermo Fisher Scientific (Pittsburgh, PA).

### Chemical synthesis

Anhydrous DMF (dimethylformamide), high-performance liquid chromatography (HPLC)–grade water, acetonitrile, dimethyl sulfoxide (DMSO), ethyl acetate, hexanes, methanol, methylene chloride (DCM), anhydrous sodium sulfate (Na_2_SO_4_), ammonium acetate (NH_4_OAC), and formic acid were purchased from Thermo Fisher Scientific (Pittsburgh, PA). 1*H*-1,2,4-triazole-3-carboxylic acid, DMSO-*d*_6_, 3,4-dimethoxyaniline, 1-hydroxybenzotriazole monohydrate (HOBt), EDC [*N*-(3-dimethylaminopropyl)-*N′*-ethylcarbodiimide], *N*-methylmorpholine (NMM), (3,4-dimethoxybenzyl)amine, (2,3-dimethoxybenzyl)amine, (4-methoxyphenyl)methanamine, 3-(trifluoromethoxy)aniline, piperonylamine, 4-(trifluoromethoxy)benzylamine, 3-methoxy-benzylamine, 3-(trifluoromethoxy)benzylamine, 4-methoxy-3-(trifluoromethyl)benzylamine, 4-(trifluoromethyl)aniline, butan-1-amine, 4-(*tert*-butyl)aniline, 3,5-dichloroaniline, 3-phenylpropan-1-amine, 4-phenylbutan-1-amine, (R)-(+)-α-methyl benzyl amine, (S)-(α-methyl benzyl amine, 2-(1*H*-indol-3-yl)ethanamine, 2-phenylethanamine, 2-(4-methoxyphenyl) ethanamine, (R)-(+)-4-methoxy-α-methylbenzylamine, and PEG400 were procured from Sigma-Aldrich Chemical Company (St. Louis, MO).

HOBt (337 mg, 2.2 mmol) and EDC (422 mg, 2.2 mmol) followed by NMM (0.88 ml, 8.0 mmol) were added via syringe to a stirred reaction of 1*H*-1,2,4-triazole-3-carboxylic acid (**1**, 226 mg, 2.0 mmol) and amine (**2**, 2.1 mmol) in dry DMF (10 ml). The mixture was stirred at room temperature (RT) under nitrogen (N_2_) until the solids were gradually dissolved. The contents were stirred at RT for 24 hours, then slowly diluted into ice-cold water, and extracted twice with 50 ml of DCM. The DCM phase was washed twice with 100 ml of cold water. The DCM phase was dried over anhydrous Na_2_SO_4_, filtered, concentrated under reduced pressure, and then chromatographed on silica gel to get the desired analog.

Chemical synthesis reactions were monitored via silica gel IB2-F thin-layer chromatography plates from J. T. Baker (Phillipsburg, NJ). Silica Gel (60 Å, 40 to 63 μm) was purchased from Sorbent Technologies (Norcross, GA). The ^1^H (proton) and ^19^F NMR spectra were recorded using a 400-MHz Bruker NMR, Avance III 400. The chemical shifts are reported in parts per million. An Applied Biosystems Sciex 4000 (Applied Biosystems; Foster City, CA) was equipped with a Shimadzu HPLC (Shimadzu Scientific Instruments Inc., Columbia, MD), and a Leap auto-sampler (LEAP Technologies, Carrboro, NC) was used to perform the LC-MS/MS. Nitrogen gas was procured from AirGas (Denver, CO). Reverse phase chromatography was performed using a Zorbax extended C18 (50 mm by 46 mm, 5-μm column) with a column guard at 40°C at a flow rate of 0.4 ml/min. Solvent A was composed of HPLC water, 10 mM NH_4_OAc, and 0.1% formic acid, whereas solvent B was composed of methanol:acetonitrile (1:1). Gradient conditions were used for the compounds and denoted for each compound (below), and compounds were monitored via electro-spray ionization positive mode (ESI+) at 450°C, curtain gas set at 10, collisional activated dissociation (CAD) set at 12 with N_2_ as the gas, and ion sources one (GS1) and two (GS2) were set at 30 with an entrance potential set at 10 V. Quadruple one (Q1) and Q3 were set to unit resolution with a dwell time of 200 ms. Samples (10 ml) were analyzed by LC-MS/MS methods (table S3).

### Disc diffusion assays

*S. enterica* serovar Typhimurium was grown in LB broth for 20 hours at 37°C with continuous shaking. One hundred microliters of overnight bacterial cultures diluted 1:1000 in phosphate-buffered saline (PBS) was spread onto M9 plates containing 1.5% Bacto agar (BD Diagnostics) in M9 minimal medium [48 mM Na_2_HPO_4_·7H_2_O, 22 mM KH_2_PO_4_, 8.56 mM NaCl, 18.69 mM NH_4_Cl, 1.0 mM MgSO_4_, 100 μM CaCl_2_, 0.2% d-glucose, and 50 μM FeSO_4_·7H_2_O (pH 7.0)]. Ten microliters of the compounds prepared 10 to 100 mM in DMSO were spotted onto 6-mm BBL blank paper discs that had been placed at the center of the plates. Plates were incubated at 37°C, and the zones of the inhibition were measured after 20 hours.

### Minimal inhibitory concentration

Bacteria, including *Salmonella*, *E. coli* strain W3110, and the other Gram-negative bacteria tested here, were grown in LB broth for 20 hours at 37°C with continuous shaking in the presence of antibiotics as appropriate. Bacterial cultures were diluted to ~10^5^ CFU/ml in EG (E salts-glucose) minimal medium [57.4 mM K_2_HPO_4_, 1.7 mM MgSO_4_, 9.5 mM citric acid, 16.7 mM H_5_NNaPO_4_, and 0.4% d-glucose (pH 7.0)] in 50-ml conical tubes (Celltreat, Pepperell, MA). Three milliliters of the diluted samples was transferred to polystyrene cell culture tubes (Life Science Frederick, CO). Antibiotics were dissolved and serially diluted in filter sterilized. Three milliliters of the diluted compounds was added at 1, 2, 8, 16, 32, 64, or 128 μg/ml into diluted bacterial cultures prepared in EG minimum medium as described above. Control cultures were treated with 3.0 ml of DMSO or 3 ml of EG minimum medium. The Δ*dksA Salmonella* mutant strain diluted in EG minimum medium was included as an additional control. MIC was determined after 20 hours of incubation at 37°C with continuously shaking.

### Killing of persistent *Salmonella*

*Salmonella* grown overnight in LB broth were diluted to ~10^5^/ml CFU in EG minimal media. One milliliter of the resulting suspensions was treated with 0.75 μM mitomycin, 8 μM 4-nitroqulinoline *N*-oxide, or 80 μg of nitrofurazone (all three purchased from Sigma-Aldrich, Milwaukee, WI) for 20 hours at 37°C. Some of the specimens were cotreated with VKT-17-P4-21 (8 μg/ml) or VKT-17-P4-23 (16 μg/ml). Percent of bacteria that survive killing was calculated after culture in LB plates.

### Antimicrobial activity against intracellular *Salmonella*

Macrophage-like J774 murine cells (ATCC TIB-67) were maintained in RPMI^+^ media [RPMI media supplemented with 2 mM l-glutamate, 1.0 mM sodium pyruvate, 15 mM Hepes buffer, 10% fetal bovine serum, and penicillin-streptomycin (100 U/ml)] in a 5% CO_2_ incubator at 37°C. The 10^5^ J774 cells per 100 μl per well were incubated in 96-well plates for 20 hours. Wild-type and mutant *Salmonella* were grown in LB broth for about 18 hours at 37°C with shaking, before dilution in RPMI^+^ media in the absence of penicillin-streptomycin. J774 cells were infected with *Salmonella* at a multiplicity of infection (MOI) of 2 in RPMI^+^ media. Plates were centrifuged for 1.0 min at 4000 rpm. After 25 min of incubation in a 5% CO_2_ incubator at 37°C, culture media was removed and replaced in RPMI^+^ medium containing gentamicin (50 μg/ml). After 1 hour, culture media in RPMI^+^ medium was supplemented with gentamicin (10 μg/ml) or gentamicin (10 μg/ml) and compound #916 (1 to 32 μg/ml). Control cells were incubated with gentamicin (10 μg/ml). J774 cells were lysed with 0.1% Triton prepared in PBS 1 and 18 hours after treatment with gentamicin (10 μg/ml). The cell lysates were spotted onto LB agar plates, and percentage survival was calculated by dividing the CFU isolated at times 0 and 18 hours.

### Antimicrobial activity against intracellular persisters

Peritoneal exudate cells were collected 4 days after C57BL/6 mice were inoculated intraperitoneally with 1 ml of filter-sterilized with sodium periodate (4 mg/ml) (Sigma-Aldrich). About 3.5 × 10^5^ peritoneal exudate cells were grown in RPMI^+^ media [RPMI media supplemented with 2 mM l-glutamate, 1 mM sodium pyruvate, 15 mM Hepes buffer, 10% fetal bovine serum, and penicillin-streptomycin (100 U/ml)] in a 96-well plate at 37°C in a 5% CO_2_ incubator for about 20 hours. Some of the cells were treated with IFN-γ (200 U/ml) for about 17 hours. Macrophages selected by adherence were infected at an MOI of 10 with wild-type Δ*dksA* or Δ*spiC Salmonella* that had been grown in LB broth for about 18 hours at 37°C with shaking and diluted in RPMI^+^ media in the absence of penicillin-streptomycin. After 25 min of incubation, the infected cells were cultured for 1 hour RPMI^+^ media containing gentamicin (50 μg/ml). The cells were then incubated in RPMI^+^ medium supplemented with gentamicin (10 μg/ml). Selected groups of cells were cotreated with compound VKT-17-P4-21 or VKT-17-P4-23 (16 μg/ml) at the time of the addition of gentamicin (10 μg/ml). Cells were lysed with 0.1% Triton prepared in PBS after 1 and 18 hours postincubation. Percent of *Salmonella* survival was calculated by recording CFU formed after overnight culture in LB agar plates.

### Intracellular SPI-2 gene transcription

J774 murine macrophage-like cells (ATCC TIB-67) were maintained in RPMI^+^ media in a 5% CO_2_ incubator at 37°C as described above. Next, 10^5^ J774 cells per 100 μl per well were incubated in 96-well plates for 20 hours. Macrophages were infected at an MOI of 20 with *sifA::LUC-*expressing *Salmonella* strains. After 25 min of incubation, infected cells were treated for 1 hour with RPMI^+^ media containing gentamicin (50 μg/ml) followed by 1 to 24 hours in RPMI^+^ media supplemented with gentamicin (10 μg/ml) or gentamicin (10 μg/ml) plus anti-DksA compound (8 to 16 μg/ml). Cells were collected 24 hours thereafter, and intracellular SPI-2 gene transcription was measured by following luciferase-mediated luminescence with the One-Glo luciferase kit (Promega, Thermo Fisher Scientific, Madison, WI). The luciferase activity was normalized to cell number. The supernatant from infected or uninfected samples was collected after several hours of infection to measure lactate dehydrogenase release using a Roche cytotoxicity detection kit (Roche, Indianapolis, IN).

### Overexpression and purification of His-tagged DksA proteins

The *dksA and greAB* genes were cloned into the C-terminal 6×His fusion vector pET-22b(+) (Novagen) and the N-terminal 6×His fusion vector pET-14 (Novagen), respectively. The plasmids were expressed in *E. coli* BL21 (DE3) (Invitrogen) (table S5). Cells were grown in LB broth at 37°C until reaching an optical density at 600 nm of 0.5 to 0.7, followed by induction with 0.1 mM isopropyl-β-d-thiogalactopyranoside. After 3 hours, cells were harvested, disrupted by sonication, and centrifuged to obtain cell-free supernatants. His-tagged DksA proteins were purified using TALON metal-affinity chromatography (Clontech) following the manufacturer’s protocol. Purified proteins were aliquoted and stored anaerobically in a BACTRON chamber (Shel Lab). Protein purity and molecular weight were confirmed by SDS–polyacrylamide gel electrophoresis.

### DksA-dependent in vitro transcription

In vitro transcription reactions were quantified using real-time PCR, following previously established methods (*35*). Briefly, DNA templates for *rpsM*, *hisG*, and *livJ* were cloned into pTim vector, which contains two Rho-independent transcriptional terminators (*35*) as detailed in table S6. DNA plasmids containing the target promoters (10 nM) were mixed with increasing concentrations of anti-DksA compounds in reaction buffer [40 mM Hepes (pH 7.4), 2 mM MgCl_2_, 60 mM potassium glutamate, 0.1% NP-40, 200 μM of each adenosine triphosphate, guanosine triphosphate, and uridine triphosphate, 8 U of RiboLock ribonuclease (RNase) inhibitor (Thermo Fisher Scientific), 5 nM *E. coli* RNA polymerse (holoenzyme; New England Biolabs), and 5 μM DksA proteins] in a final volume of 10 μl. Reactions were incubated at 37°C for 10 min, followed by termination at 70°C for 10 min. After deoxyribonuclease I treatment, template DNA was removed using the DNA-free DNA Removal Kit (Thermo Fisher Scientific). The resulting RNA preparations were reverse transcribed into cDNA using 100 U of MMLV reverse transcriptase (Promega), 0.45 μM N6 random hexamer primers (Thermo Fisher Scientific), and 20 U of RNase inhibitor (Promega). The amount of cDNA synthesized following 1 hour of incubation at 42°C was quantified by qRT-PCR using gene-specific primers and probes presented in table S7. Specific transcripts were normalized against standard curves generated from known amounts of template.

### Binding of compound to DksA

Binding of DksA, GreA, and GreB recombinant proteins to the compound was evaluated using MicroScale Thermophoresis by detecting temperature-induced changes in fluorescence of target protein in the presence of increasing concentrations of the anti-DksA compound VKT-17-P4-23. Briefly, 100 nM 6His recombinant protein was labeled with NT647 fluorescent dye using the Monolith NT His-Tag Labeling Kit RED-Tris-NTA (Nano Temper Technologies). The compound was serially diluted in PBS buffer with 5% DMSO, then mixed with an equal volume of NT647-labeled DksA protein (20 nM), and loaded into capillaries. Binding was measured on Monolith NT.115 at a light-emitting diode/excitation and MST power of 40% at Biophysics Core in the University of Colorado School of Medicine. *K*_d_ values from three independent experiments were determined from a dose-response curve, which was fitted to a one-site binding model using MO.Affinity Analysis v2.3.

### Pharmacokinetics

The in vivo portion was conducted at the University of Colorado Anschutz medical campus (Aurora, CO), in an Association for Assessment and of Laboratory Animal Care International accredited facility. All procedures were reviewed and approved by the University of Colorado, Medical Campus Committee on Animal Care; the research procedures adhered to the Principles of Laboratory Animal Care (National Institutes of Health publication no. 85-23, revised in 1985). Given their limited solubility, the compounds were prepared in PEG400. As a probe study, fasted male Sprague-Dawley rats (225 to 250 g) (*N* = 1 each for intraperitoneal and per oral (p.o.) for VKT-17-P4-21 and VKT-17-P4-23 (four rats in total) were dosed via intraperitoneal at 1.0 mg/kg and orally at 10.0 mg/kg using dose solutions of VKT-17-P4-21 and VKT-17-P4-23 formulated in PEG400 at 10.0 mg/ml. At various time points, blood samples (125 μl) were collected via tail veil collection using EDTA collection tubes, snap frozen, extracted via organic solvent extraction (250 μl) at a later time, and centrifuged at 11,000 rpm for 5.0 min, and supernatants were transferred to 96-well plates and analyzed by LC-MS/MS methods; standard curves were prepared from control rat blood.

Using the protocol 00720 approved by the University of Colorado Denver Institutional Animal Care and Use Committee, male Sprague-Dawley rats weighing ~292 ± 14 g on a standard rodent diet were fasted 12 hours before the study and dosed either intravenously through the lateral caudal tail vein or orally using oral gavage. All rats were administered VKT-17-P4-23 either intravenously at 1.0 mg/kg or orally dosed at 10 mg/kg based on each rat’s measured weight. A sample of five rats was dosed intravenously and was given a standard diet shortly after dosing, while another five rats were given dosed orally and were fasted for an additional 2 hours after dosing. Time zero began when each rat was administered the dose, and blood samples of 125 μl from the tail were collected using capillary tubes over a 24-hour period at approximate time points (*t*): *t* = 10 min, 30 min, 45 min, 60 min, 1.5 hours, 2 hours, 3 hours, 4 hours, 6 hours, 8 hours, 12 hours, and 24 hours. Blood samples were stored at <−70°C. Samples were brought to RT for work-up and mixed in a solution of 1:1 acetonitrile and methanol with VKT-17-P4-21, acting as the internal standard. Blood concentrations of VKT-17-P4-23 were analyzed via LC-MS/MS. An Applied Biosystems Sciex 4000 (Applied Biosystems, Foster City, CA) was equipped with a Shimadzu HPLC (Shimadzu Scientific Instruments Inc., Columbia, MD) and a Nexera X2 Sil-30 AC autosampler (Shimadzu Scientific Instruments Inc., Columbia, MD). The LC-MS/MS method used a Zorbax extend-C18 column (4.6 mm by 50 mm) equipped with a C18 column guard. The flow rate was 0.4 ml/min and solvent A was composed of H2O, 10 mM NH4OAc, 0.1% formic acid, and solvent B was composed of methanol:acetonitrile (1:1). The gradient conditions were as follows: 95% A for 0.5 min and then ramped to 95% B at 3.0 min and held for 2.0 min, at which point it was ramped back to 95% A at 6.0 min and held for 1.0 min (7.0-min total run time). VKT-17-P4-23 [245.15 ➔ 91.00 mass/charge ratio (*m/z*)] *t*_R_ = 4.3 min with a DP = 86, CE = 39, and CXP = 6. The analysis was performed in ESI (+) ion mode, and other instrument parameters were as follows: GS1/GS2 set to 35/50, temperature of 450°C, CAD = 12, CUR = 20, IS = 5500, and Q1/Q3 set to unit/unit resolution. VKT-17-P4-21 (internal standard; 257.00 ➔ 96.20 *m/z*) *t*_R_ = 4.6 min with a DP = 91, CE = 33, and CXP = 1.

### Pharmacodynamics

Tissues were weighed and snap frozen, stored at (−80° ± 10°C) until processed which involved cutting the tissues into small pieces, adding two volumes of PBS (pH 7.4) by tissue weight, and homogenizing using a hand-held homogenizer. Samples were taken in triplicate (200 μl) and extracted with extraction solution (1:1 methanol:acetonitrile containing VKT-17-P4-21 as an internal standard; 400 μl), vortex mixed (5 s), and centrifuged at 11,000 rpm for 5.0 min. The supernatants were transferred to 96-well plates and analyzed by LC-MS/MS analysis. Control tissues were processed, spiked with known amounts of VKT-17-P4-23, and used to prepare standard curves. The concentrations were adjusted to total organ weights.

### Ethics statement

This study was performed in accordance with the recommendations in the Guide for the Care and Use of Laboratory Animals of the National Institutes of Health. All animals were handled according to approved Institutional Animal Care and Use Committee protocol 00059 of the University of Colorado School of Medicine (assurance number A3269-01).

### Antibiotic activity in acute models of *Salmonella* infection

C57BL/6 mice, which were bred in the animal facility located at the University of Colorado School of Medicine following Institutional Animal Care and Use Committee guidelines, were used at 6 to 8 weeks of age. The mice were deprived of food and water 4 hours before oral inoculation of 100 μl of filter-sterilized streptomycin (200 mg/ml). After 24 hours of streptomycin treatment, the mice were inoculated by oral gavage with 10^8^ CFU of *S.* Typhimurium strain 14028s. Compound VKT-17-P4-23 was also tested in a systemic model of acute salmonellosis. *Salmonella* grown overnight in LB broth were diluted to 400 CFU/ml in PBS. Mice were inoculated intraperitoneally with 500 μl of the resulting bacterial suspension. Mice infected with *Salmonella* through oral gavage or intraperitoneal inoculation had been injected intraperitoneal with 200 μl of VKT-17-P4-23 (1.5 mg/ml) 1 hour before infection with *Salmonella*. Mice were dosed with the compound every 12 hours for three times postinoculation. Mice were euthanized 48 hours after *Salmonella* challenged. Livers and spleens were collected to determine bacterial burden after plating in LB agar containing the appropriate antibiotics. Tissue was also collected for histopathological analysis.

### Liver toxicity

C57BL/6 mice were infected intraperitoneally with about 200 CFU of *Salmonella* for 2 days. Where indicated, the mice were treated with compound VKT-17-P4-23 as described above. Naïve mice were included as controls. Blood was collected by cardiac puncture in tubes containing the anticoagulant heparin. The blood was centrifuged at 1000*g* at 4°C for 10 min. AST and ALT were assayed in plasma according to kits purchased from Ray Bio (Peachtree Corners, GA). Briefly, working solution, prepared by mixing assay buffer and enzyme mix solution at a volumetric ratio of 5:1, was mixed well and incubated at 37°C for 5 min. Ten microliters of plasma was pipetted into each well of the 96-well plate containing 100 μl of prewarmed working solution. The specimens were incubated at 37°C for 1 min. The absorbance at 340 nm was recorded at 1-min intervals for 5 min. Enzymatic activity was determined by regression analysis from standard curves prepared from known concentrations of AST and ALT provided by the manufacturer.

### Histopathology

Hepatic and splenic tissue were fixed in formalin and processed by the Research Pathology Histology Core, University of Colorado Anschutz Medical Campus for paraffin embedding, 4-μm serial microtome sectioning, and hematoxylin and eosin (H&E) staining. The H&E-stained specimens were observed and scored blinded under light microscopy. The average number of granulomas and necrotic foci per 200× field of liver and spleen H&E-stained sections were quantified.
