# Supplementary material for: DksA inhibitors against intracellular and persistent Salmonella are effective in acute models of infection
Source: Sci Adv. 2026 Mar 18;12(12):eaea6832. doi: 10.1126/sciadv.aea6832 (PMC12998499; doi:10.1126/sciadv.aea6832)
Supplement: Supplementary file 1 — Tables S1 to S7 Figs. S1 to S5 [file sciadv.aea6832_sm.pdf]

Science Advances
AAAS

Supplementary Materials for

DksA inhibitors against intracellular and persistent *Salmonella* are effective in acute models of infection

Ju-Sim Kim *et al.*

Corresponding author: Andrés Vázquez-Torres, andres.vazquez-torres@cuanschutz.edu

Sci. Adv. **12**, eaea6832 (2026)
DOI: 10.1126/sciadv.aea6832

This PDF file includes:

Tables S1 to S7
Figs. S1 to S5

Table S1. Primary screen for drugs against DksA.

Rank<br>ZINC ID<br>Score kcal/mol	Compound	Availability	Properties
1<br>63887176<br>-71.24	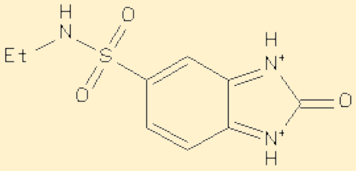	Molport:MolPort-008-431-209	Mwt: 241.272<br>xLogP: 0.46<br>Charge: 2<br>RotBond: 3<br># Protomers: 1<br>Contact: 2<br>ES: -42.22 VdW: -10.06<br>Desolv: p=-19.44, ap=0.48
2<br>39962616<br>-65.54	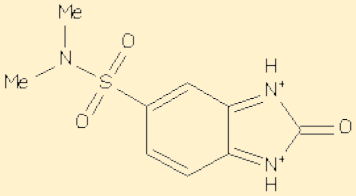	Molport:MolPort-002-257-396	Mwt: 241.272<br>xLogP: 0.33<br>Charge: 2<br>RotBond: 2<br># Protomers: 1<br>Contact: 2<br>ES: -39.40 VdW: -11.85<br>Desolv: p=-14.67, ap=0.39
3<br>86864968<br>-40.01	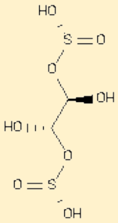	FineTech:FT-0626795	Mwt: 222.196<br>xLogP: -5.03<br>Charge: 0<br>RotBond: 5<br># Protomers: 1<br>Contact: 5<br>ES: -43.10 VdW: -5.14<br>Desolv: p=5.73, ap=2.49
4<br>35499758<br>-38	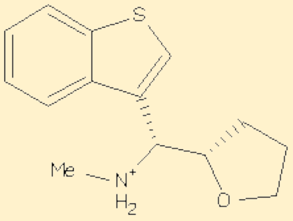	AKOS (make-on-demand):AKOS005838099<br>UORSY BB Make-on-demand:BBV-24892223<br>Enamine BB Make on Demand:BBV-24892223	Mwt: 248.371<br>xLogP: 2.95<br>Charge: 1<br>RotBond: 3<br># Protomers: 2<br>Contact: 2<br>ES: -45.50 VdW: -7.00<br>Desolv: p=15.75, ap=-1.25
5<br>37414328<br>-37.46	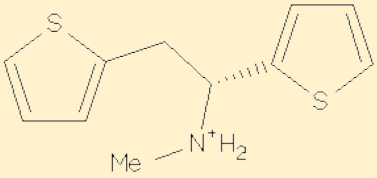	Enamine BB Make on Demand:BBV-27229043	Mwt: 224.374<br>xLogP: 3.28<br>Charge: 1<br>RotBond: 4<br># Protomers: 2<br>Contact: 2<br>ES: -42.22 VdW: -5.75<br>Desolv: p=11.61, ap=-1.10

6
22029086
-37.26

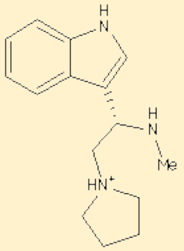

BioSynth:Q-102926
Fluorochem:011783
ChemMol:44020230
Ark Pharm Building Blocks:AK-41131
Chembo Pharma:KB-63686

Mwt: 244.362
xLogP: 2.26
Charge: 1
RotBond: 4
Protomers: 2
Contact: 2
ES: -43.44 VdW: -6.08
Desolv: p=12.83, ap=-0.57

7
78712051
-37.08

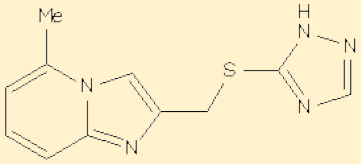

Enamine-REAL:Z277049612

Mwt: 245.311
xLogP: 1.34
Charge: 0
RotBond: 3
Protomers: 4
Contact: 2
ES: -30.61 VdW: -14.06
Desolv: p=9.37, ap=-1.78

8
71791704
-37

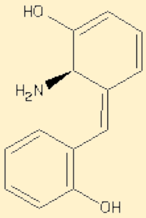

Tractus:TRA0033437

Mwt: 216.26
xLogP: 1.79
Charge: 1
RotBond: 1
Protomers: 5
Contact: 3
ES: -41.63 VdW: -11.87
Desolv: p=15.61, ap=0.89

9
86864967
-36.97

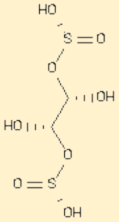

FineTech:FT-0626795

Mwt: 222.196
xLogP: -5.03
Charge: 0
RotBond: 5
Protomers: 1
Contact: 5
ES: -40.84 VdW: -5.48
Desolv: p=6.82, ap=2.52

10
63784927
-36.58

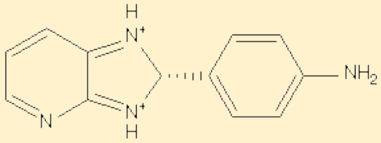

Molport:MolPort-008-319-228

Mwt: 213.264
xLogP: 1.54
Charge: 3
RotBond: 1
Protomers: 2
Contact: 1
ES: -24.65 VdW: -3.83
Desolv: p=-8.29, ap=0.18

11
63784929
-36.56

H

Mwt: 213.264
xLogP: 1.54
Charge: 3

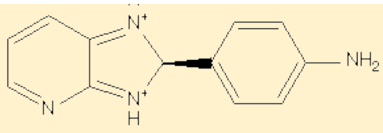

Molport:MolPort-008-319-228

RotBond: 1
Protomers: 2
Contact: 1
ES: -24.70 VdW: -3.78
Desolv: p=-8.29, ap=0.21

12
74535703
-36.55

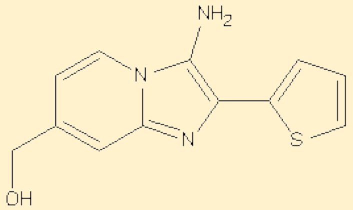

Not for sale

Mwt: 245.307
xLogP: 1.65
Charge: 0
RotBond: 2
Protomers: 2
Contact: 3
ES: -26.59 VdW: -16.31
Desolv: p=7.30, ap=-0.95

13
82961298
-36.37

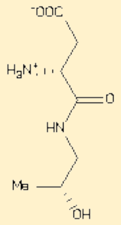

UORSY BB Make-on-demand:BBV-39239665
Enamine BB Make on Demand:BBV-39239665

Mwt: 190.199
xLogP: -2.47
Charge: 0
RotBond: 5
Protomers: 2
Contact: 2
ES: -41.18 VdW: -4.50
Desolv: p=7.82, ap=1.49

14
82302932
-36.29

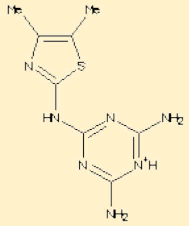

Not for sale

Mwt: 238.3
xLogP: 0.72
Charge: 1
RotBond: 2
Protomers: 2
Contact: 2
ES: -28.95 VdW: -15.93
Desolv: p=10.65, ap=-2.06

15
44608383
-36.25

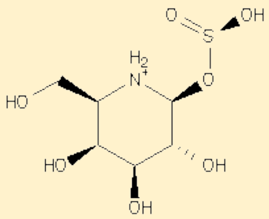

Toronto Research Chemicals:G156500

Mwt: 244.245
xLogP: -4.60
Charge: 1
RotBond: 3
Protomers: 2
Contact: 6
ES: -43.71 VdW: -6.68
Desolv: p=10.06, ap=4.07

16
82306654
-36.2

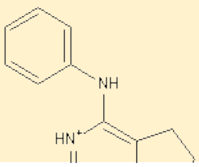

Not for sale

Mwt: 242.306
xLogP: 2.34
Charge: 1
RotBond: 2
Protomers: 3
Contact: 2

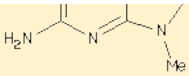

ES: -30.86 VdW: -11.97
Desolv: p=8.06, ap=-1.44

17
76078915
-35.98

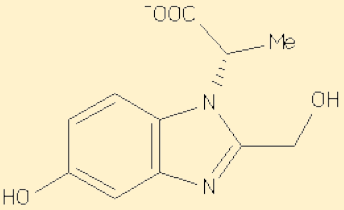

Not for sale

Mwt: 235.219
xLogP: 0.05
Charge: -1
RotBond: 3
Protomers: 3
Contact: 4
ES: -29.23 VdW: -12.53
Desolv: p=5.95, ap=-0.17

18
76079798
-35.89

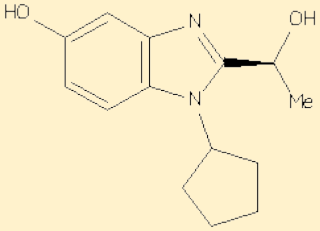

Not for sale

Mwt: 246.31
xLogP: 2.06
Charge: 0
RotBond: 2
Protomers: 3
Contact: 4
ES: -31.61 VdW: -11.67
Desolv: p=7.39, ap=0.01

19
22927770
-35.88

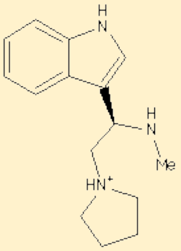

BioSynth:Q-102926
Fluorochem:011783
ChemMol:44020230
Ark Pharm Building Blocks:AK-41131
Chembo Pharma:KB-63686

Mwt: 244.362
xLogP: 2.26
Charge: 1
RotBond: 4
Protomers: 2
Contact: 2
ES: -35.48 VdW: -8.79
Desolv: p=9.52, ap=-1.13

20
83341505
-35.88

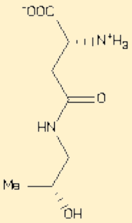

Not for sale

Mwt: 190.199
xLogP: -2.70
Charge: 0
RotBond: 5
Protomers: 2
Contact: 2
ES: -40.61 VdW: -3.69
Desolv: p=7.22, ap=1.20

21
82961297
-35.81

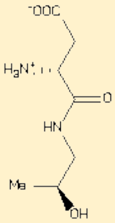

UORSY BB Make-on-demand:BBV-39239665
Enamine BB Make on Demand:BBV-39239665

Mwt: 190.199
xLogP: -2.47
Charge: 0
RotBond: 5
Protomers: 2
Contact: 2
ES: -40.07 VdW: -5.11
Desolv: p=8.02, ap=1.35

22
83072925
-35.73

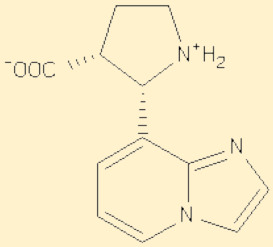

Not for sale

Mwt: 231.255
xLogP: 0.74
Charge: 0
RotBond: 2
Protomers: 3
Contact: 2
ES: -39.65 VdW: -10.76
Desolv: p=15.93, ap=-1.25

23
41121431
-35.72

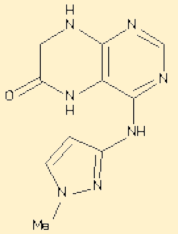

Molport:MolPort-019-848-285
eMolecules:30314169
Princeton BioMolecular Research:OSSL_317569

Mwt: 245.246
xLogP: 0.24
Charge: 0
RotBond: 2
Protomers: 2
Contact: 2
ES: -32.51 VdW: -14.59
Desolv: p=11.91, ap=-0.53

24
76079608
-35.69

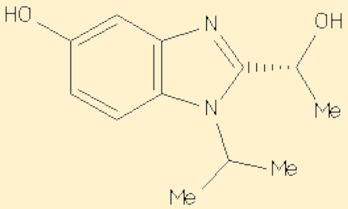

Not for sale

Mwt: 220.272
xLogP: 1.40
Charge: 0
RotBond: 2
Protomers: 3
Contact: 3
ES: -33.20 VdW: -9.49
Desolv: p=6.97, ap=0.03

25
44608384
-35.67

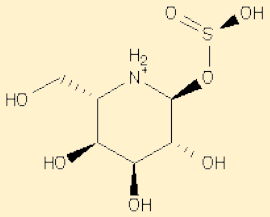

Toronto Research Chemicals:G156500

Mwt: 243.237
xLogP: -4.60
Charge: 0
RotBond: 3
Protomers: 2
Contact: 6
ES: -47.42 VdW: -2.87
Desolv: p=12.63, ap=1.98

26
83040986
-35.64

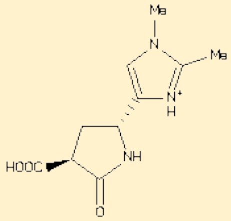

Not for sale

Mwt: 223.232
xLogP: -0.87
Charge: 0
RotBond: 2
Protomers: 3
Contact: 2
ES: -36.78 VdW: -9.28
Desolv: p=10.66, ap=-0.23

27
348750
-35.6

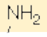

ChemBridge:5856520
eMolecules:2376429
Molport:MolPort-002-172-579

Mwt: 225.275
xLogP: 3.20
Charge: 1

	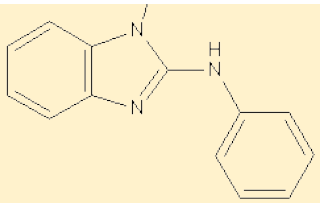	Mcule Make-on-demand:MCULE-1830980531<br>Mcule:MCULE-1830980531	RotBond: 2<br># Protomers: 2<br>Contact: 2<br>ES: -25.76 VdW: -16.02<br>Desolv: p=8.15, ap=-1.97
28<br>86864969<br>-35.6	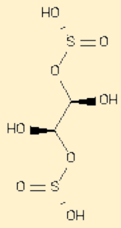	FineTech:FT-0626795	Mwt: 222.196<br>xLogP: -5.03<br>Charge: 0<br>RotBond: 5<br># Protomers: 1<br>Contact: 5<br>ES: -38.27 VdW: -4.69<br>Desolv: p=5.02, ap=2.34
29<br>20052852<br>-35.57	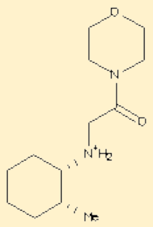	UORSY BB Make-on-demand:BBV-150185<br>Enamine BB Make on Demand:BBV-150185	Mwt: 241.355<br>xLogP: 1.44<br>Charge: 1<br>RotBond: 3<br># Protomers: 2<br>Contact: 2<br>ES: -42.76 VdW: -6.03<br>Desolv: p=13.86, ap=-0.63
30<br>40707504<br>-35.57	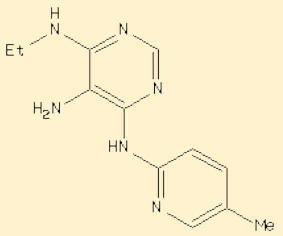	Innovapharm Make-on-Demand:VT-00319973	Mwt: 244.302<br>xLogP: 1.67<br>Charge: 0<br>RotBond: 4<br># Protomers: 4<br>Contact: 3<br>ES: -29.89 VdW: -13.72<br>Desolv: p=8.72, ap=-0.69
31<br>76137439<br>-35.54	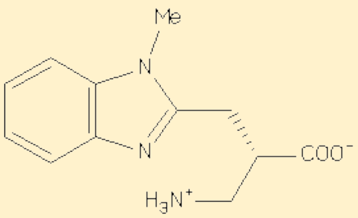	Not for sale	Mwt: 233.271<br>xLogP: 0.20<br>Charge: 0<br>RotBond: 4<br># Protomers: 3<br>Contact: 2<br>ES: -38.43 VdW: -13.69<br>Desolv: p=17.68, ap=-1.10
32<br>82309287<br>-35.51	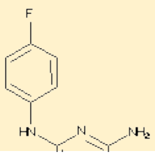	Not for sale	Mwt: 245.217<br>xLogP: 1.44<br>Charge: 0<br>RotBond: 2<br># Protomers: 3<br>Contact: 2

	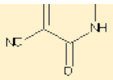		ES: -27.07 VdW: -12.26<br>Desolv: p=5.00, ap=-1.17
33<br>82961226<br>-35.51	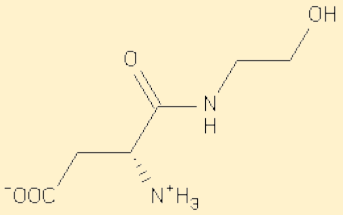	UORSY BB Make-on-demand:BBV-39239631<br>Enamine BB Make on Demand:BBV-39239631	Mwt: 175.164<br>xLogP: -2.83<br>Charge: -1<br>RotBond: 5<br># Protomers: 2<br>Contact: 2<br>ES: -40.25 VdW: -4.65<br>Desolv: p=8.04, ap=1.35
34<br>76137595<br>-35.5	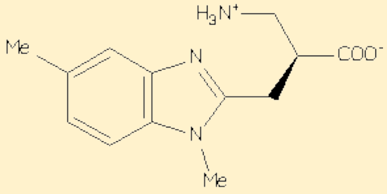	Not for sale	Mwt: 247.298<br>xLogP: 0.63<br>Charge: 0<br>RotBond: 4<br># Protomers: 3<br>Contact: 2<br>ES: -37.34 VdW: -13.15<br>Desolv: p=16.50, ap=-1.51
35<br>82293745<br>-35.43	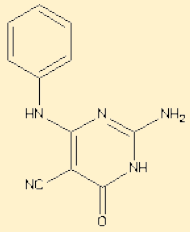	Not for sale	Mwt: 227.227<br>xLogP: 1.28<br>Charge: 0<br>RotBond: 2<br># Protomers: 3<br>Contact: 2<br>ES: -25.86 VdW: -12.69<br>Desolv: p=4.32, ap=-1.20
36<br>82373583<br>-35.34	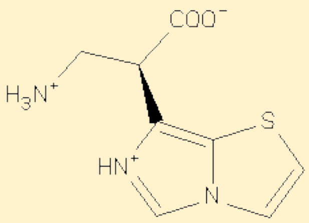	Not for sale	Mwt: 212.254<br>xLogP: -0.90<br>Charge: 1<br>RotBond: 3<br># Protomers: 4<br>Contact: 2<br>ES: -41.62 VdW: -8.79<br>Desolv: p=16.34, ap=-1.26
37<br>205965<br>-35.31	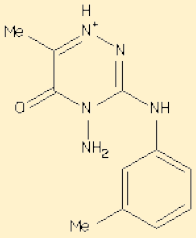	Asinex:BAS06970481<br>eMolecules:1489382<br>Innovapharm:STT-00108872<br>TimTec:ST50102139<br>Innovapharm Building Blocks:BBS-00014350<br>Molport BB:MolPort-000-466-922<br>Mcule Make-on-demand:MCULE-4238477832<br>ChemDiv:6404-0167	Mwt: 232.267<br>xLogP: 1.56<br>Charge: 1<br>RotBond: 2<br># Protomers: 2<br>Contact: 2<br>ES: -28.15 VdW: -17.00<br>Desolv: p=11.25, ap=-1.41

...

38
3830252
-35.31

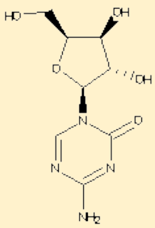

Scientific Exchange (make on demand):F-137758
AKOS:AKOS003382387
Element Store BB:b2star-320-67-2
Tractus:TRA0051143
Ambinter:Amb19654023
Lab Seeker Building Blocks:SC-60636
StruChem BB make-on-demand:SC-60636
Mcule:MCULE-7974162026
...

Mwt: 244.207
xLogP: -2.34
Charge: 0
RotBond: 2
Protomers: 2
Contact: 2
ES: -46.21 VdW: -8.85
Desolv: p=19.25, ap=0.50

39
36243234
-35.31

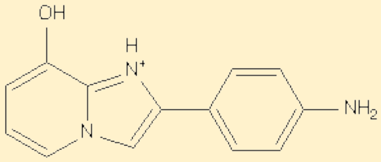

Not for sale

Mwt: 226.259
xLogP: 1.78
Charge: 1
RotBond: 1
Protomers: 3
Contact: 3
ES: -28.31 VdW: -14.45
Desolv: p=8.68, ap=-1.22

40
1706315
-35.3

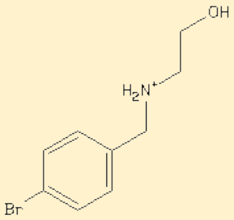

Fluorochem:085817
ChemBridge BuildingBlocks:7732645
Innovapharm BB Make on Demand:BBV-00041808
ChemBridge:7732645
Princeton BioMolecular Research:OSSK_618556
Mcule Make-on-demand:MCULE-1782578881
eMolecules:1787757
Oakwood Chemical:085817
...

Mwt: 231.113
xLogP: 1.67
Charge: 1
RotBond: 4
Protomers: 1
Contact: 2
ES: -43.19 VdW: -5.81
Desolv: p=14.57, ap=-0.87

41
36243233
-35.27

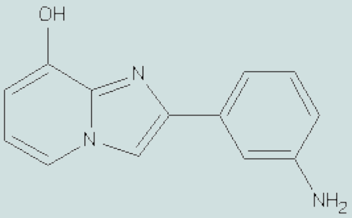

Not for sale

Mwt: 225.251
xLogP: 1.76
Charge: 0
RotBond: 1
Protomers: 3
Contact: 3
ES: -28.89 VdW: -14.14
Desolv: p=8.83, ap=-1.08

42
13353968
-35.25

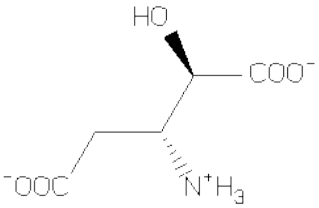

BOC Sciences BB:174320-28-6

Mwt: 162.121
xLogP: -2.71
Charge: -1
RotBond: 4
Protomers: 1
Contact: 2
ES: -30.83 VdW: -5.12
Desolv: p=-0.57, ap=1.27

43
39236016
-35.21

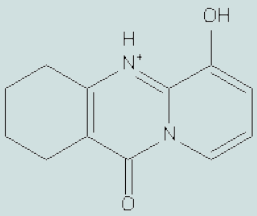

ChemBridge:9200099
eMolecules:32356083
TimTec:ST45374528
Mcule:MCULE-6942596542
Mcule Make-on-demand:MCULE-6942596542
Princeton BioMolecular Research:OSSL_789532

Mwt: 217.248
xLogP: 1.96
Charge: 1
RotBond: 0
Protomers: 2
Contact: 2
ES: -28.47 VdW: -13.28
Desolv: p=7.60, ap=-1.06

44
36427679
-35.13

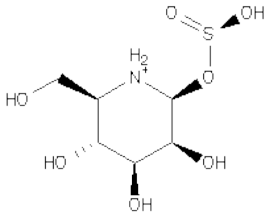

Tractus:TRA0013915

Mwt: 244.245
xLogP: -4.60
Charge: 1
RotBond: 3
Protomers: 2
Contact: 6
ES: -43.92 VdW: -6.00
Desolv: p=12.24, ap=2.55

45
4969134
-35.09

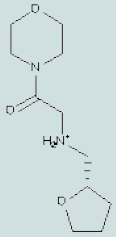

Asinex:ASN08253370
UORSY BB Make-on-demand:BBV-151544
Enamine BB Make on Demand:BBV-151544
Aronis BB Make-on-demand:BLOCK45693
Ambinter:Amb9748897
TimTec:ST50314061
AKOS:AKOS000257630

Mwt: 229.3
xLogP: -0.68
Charge: 1
RotBond: 4
Protomers: 1
Contact: 2
ES: -43.15 VdW: -4.39
Desolv: p=12.61, ap=-0.16

46
82378167
-35.09

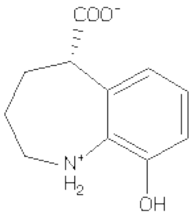

Not for sale

Mwt: 206.221
xLogP: 1.10
Charge: -1
RotBond: 1
Protomers: 2
Contact: 2
ES: -29.18 VdW: -9.34
Desolv: p=3.85, ap=-0.43

47
44995994
-35.03

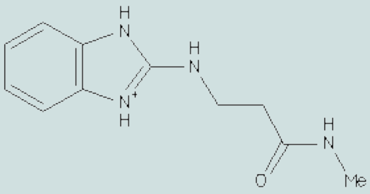

UORSY BB Make-on-demand:BBV-36635499
Enamine BB Make on Demand:BBV-36635499

Mwt: 219.268
xLogP: 0.98
Charge: 1
RotBond: 4
Protomers: 2
Contact: 2
ES: -31.20 VdW: -13.00
Desolv: p=10.05, ap=-0.87

48
82296922
-35

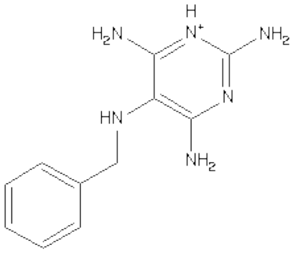

Not for sale

Mwt: 231.283
xLogP: 0.43
Charge: 1
RotBond: 3
Protomers: 2
Contact: 2
ES: -26.33 VdW: -17.51
Desolv: p=9.32, ap=-0.48

49
76137545
-34.99

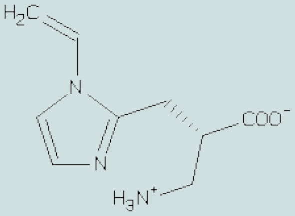

Not for sale

Mwt: 195.222
xLogP: -1.00
Charge: 0
RotBond: 5
Protomers: 3
Contact: 3
ES: -45.16 VdW: -7.75
Desolv: p=18.48, ap=-0.55

50
26421837
-34.97

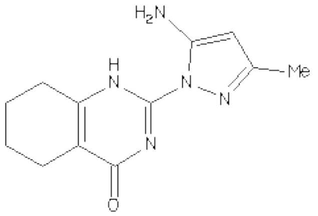

Innovapharm BB Make on Demand:BBV-00051867
Life Chemicals:F2135-0896
Molport BB:MolPort-007-995-370
Molport:MolPort-007-995-370
Life Chemicals Building Blocks:F2135-0896

Mwt: 246.294
xLogP: 1.15
Charge: 1
RotBond: 1
Protomers: 6
Contact: 2
ES: -29.38 VdW: -16.06
Desolv: p=11.86, ap=-1.40

51
5387040
-34.95

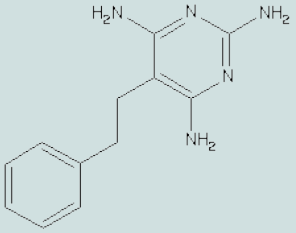

NCI Plated 2007:337828

Mwt: 229.287
xLogP: 1.16
Charge: 0
RotBond: 3
Protomers: 2
Contact: 2
ES: -26.41 VdW: -17.11
Desolv: p=9.73, ap=-1.17

52
75853001
-34.93

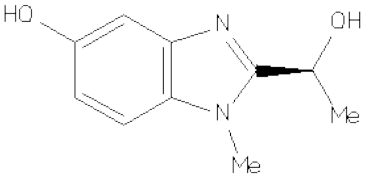

Not for sale

Mwt: 192.218
xLogP: 0.66
Charge: 0
RotBond: 1
Protomers: 3
Contact: 3
ES: -40.33 VdW: -1.52
Desolv: p=6.25, ap=0.68

53
82293753

Mwt: 227.231
xLogP: 0.27

-34.93	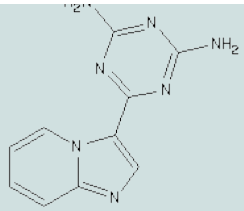	Not for sale	Charge: 0<br>RotBond: 1<br># Protomers: 2<br>Contact: 2<br>ES: -25.79 VdW: -17.07<br>Desolv: p=9.81, ap=-1.88
54<br>40685291<br>-34.86	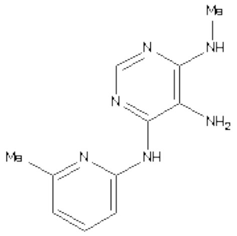	Innovapharm Make-on-Demand:VT-00313730	Mwt: 230.275<br>xLogP: 0.89<br>Charge: 0<br>RotBond: 3<br># Protomers: 4<br>Contact: 3<br>ES: -27.58 VdW: -12.56<br>Desolv: p=6.50, ap=-1.22
55<br>19903635<br>-34.85	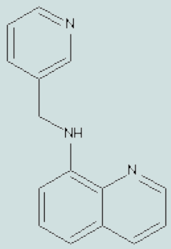	Ambinter:Amb6689120<br>UORSY BB Make-on-demand:BBV-123400<br>Enamine BB Make on Demand:BBV-123400	Mwt: 235.29<br>xLogP: 1.91<br>Charge: 0<br>RotBond: 3<br># Protomers: 3<br>Contact: 2<br>ES: -28.31 VdW: -16.65<br>Desolv: p=11.47, ap=-1.37
56<br>13532478<br>-34.84	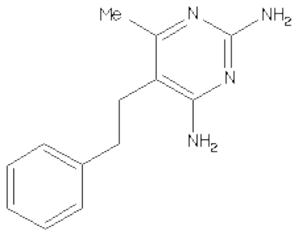	Molport:MolPort-019-786-819	Mwt: 228.299<br>xLogP: 1.77<br>Charge: 0<br>RotBond: 3<br># Protomers: 3<br>Contact: 2<br>ES: -26.48 VdW: -16.42<br>Desolv: p=9.48, ap=-1.42
57<br>76137601<br>-34.8	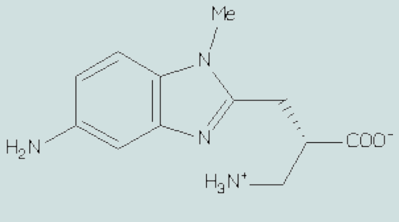	Not for sale	Mwt: 248.286<br>xLogP: -0.75<br>Charge: 0<br>RotBond: 4<br># Protomers: 3<br>Contact: 2<br>ES: -34.71 VdW: -15.14<br>Desolv: p=15.97, ap=-0.92
58<br>13637018<br>-34.79	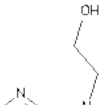	Scientific Exchange (make on demand):F-211488<br>Vitas-M:STK397903<br>Princeton BioMolecular Research:OSSL_150375<br>eMolecules:14275704	Mwt: 243.266<br>xLogP: 1.20<br>Charge: 0<br>RotBond: 2

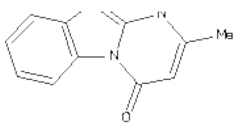

Chemical Block:A4442/0189773
Pharmeks:PHAR235581
IBScreen:STOCK6S-73326
Molport:MolPort-005-310-215
...

Protomers: 2
Contact: 2
ES: -29.76 VdW: -14.30
Desolv: p=10.71, ap=-1.44

59
38017795

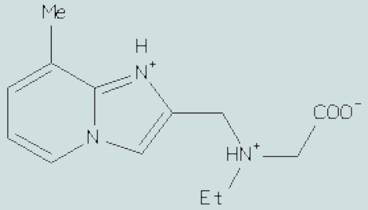

UORSY BB Make-on-demand:BBV-32288478
Enamine BB Make on Demand:BBV-32288478

Mwt: 248.306
xLogP: 0.45
Charge: 1
RotBond: 5
Protomers: 2
Contact: 2
ES: -34.41 VdW: -11.59
Desolv: p=12.91, ap=-1.69

60
40723501
-34.75

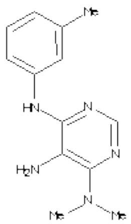

Innovapharm Make-on-Demand:VT-00321625

Mwt: 243.314
xLogP: 2.41
Charge: 0
RotBond: 3
Protomers: 2
Contact: 3
ES: -27.81 VdW: -14.81
Desolv: p=9.57, ap=-1.70

61
40701240
-34.66

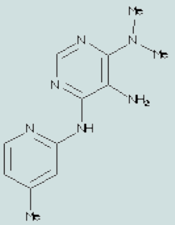

Innovapharm Make-on-Demand:VT-00316574

Mwt: 244.302
xLogP: 1.48
Charge: 0
RotBond: 3
Protomers: 4
Contact: 3
ES: -29.10 VdW: -12.56
Desolv: p=8.60, ap=-1.60

62
32004970
-34.64

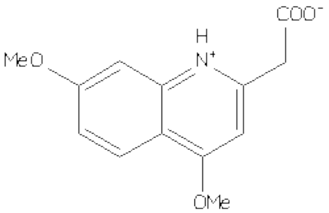

Not for sale

Mwt: 247.25
xLogP: 2.15
Charge: 0
RotBond: 4
Protomers: 2
Contact: 3
ES: -22.36 VdW: -14.46
Desolv: p=3.40, ap=-1.23

63
40707312

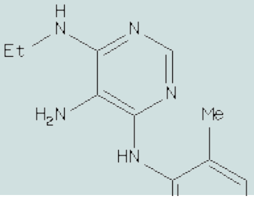

Innovapharm Make-on-Demand:VT-00319877

Mwt: 244.302
xLogP: 1.62
Charge: 0
RotBond: 4
Protomers: 4
Contact: 3
ES: -27.94 VdW: -12.66

	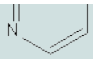		Desolv: p=7.66, ap=-1.69
64<br>38017794<br>-34.62	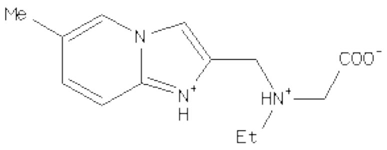	UORSY BB Make-on-demand:BBV-32288477<br>Enamine BB Make on Demand:BBV-32288477	Mwt: 248.306<br>xLogP: 0.45<br>Charge: 1<br>RotBond: 5<br># Protomers: 2<br>Contact: 2<br>ES: -33.78 VdW: -10.70<br>Desolv: p=11.32, ap=-1.46
65<br>76079609<br>-34.6	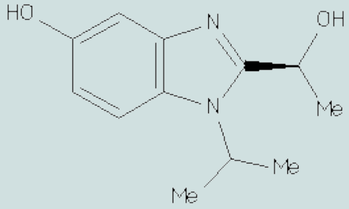	Not for sale	Mwt: 220.272<br>xLogP: 1.40<br>Charge: 0<br>RotBond: 2<br># Protomers: 3<br>Contact: 3<br>ES: -32.16 VdW: -9.23<br>Desolv: p=6.42, ap=0.37
66<br>89866717<br>-34.6	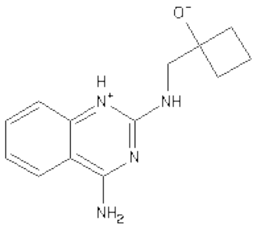	Molport:MolPort-027-670-022<br>Enamine:Z1513514898<br>Mcule:MCULE-3361811644<br>eMolecules:45688671<br>Mcule Make-on-demand:MCULE-3361811644	Mwt: 245.306<br>xLogP: 1.84<br>Charge: 1<br>RotBond: 3<br># Protomers: 3<br>Contact: 2<br>ES: -24.29 VdW: -13.25<br>Desolv: p=4.01, ap=-1.07
67<br>40549269	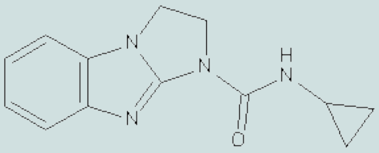	Mcule:MCULE-7053905801<br>Mcule Make-on-demand:MCULE-7053905801	Mwt: 242.282<br>xLogP: 1.80<br>Charge: 0<br>RotBond: 1<br># Protomers: 2<br>Contact: 2<br>ES: -26.97 VdW: -14.13<br>Desolv: p=8.75, ap=-2.24
68<br>82297844<br>-34.59	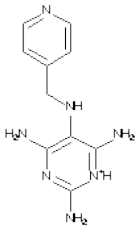	Not for sale	Mwt: 232.271<br>xLogP: -0.86<br>Charge: 1<br>RotBond: 3<br># Protomers: 3<br>Contact: 2<br>ES: -26.83 VdW: -17.65<br>Desolv: p=9.96, ap=-0.07

69
42572031
-34.55

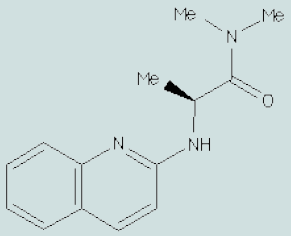

UORSY BB Make-on-demand:BBV-32906083
Enamine BB Make on Demand:BBV-32906083

Mwt: 243.31
xLogP: 1.38
Charge: 0
RotBond: 3
Protomers: 2
Contact: 2
ES: -24.08 VdW: -18.10
Desolv: p=8.84, ap=-1.20

70
74535613
-34.55

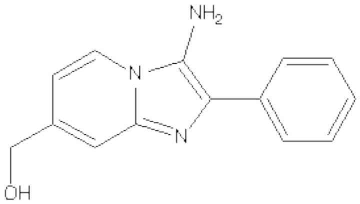

Not for sale

Mwt: 239.278
xLogP: 1.87
Charge: 0
RotBond: 2
Protomers: 2
Contact: 3
ES: -27.08 VdW: -13.88
Desolv: p=7.29, ap=-0.88

71
37756968
-34.51

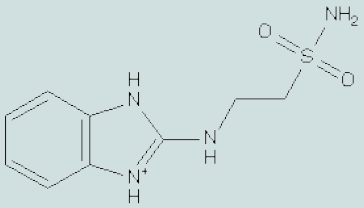

UORSY BB Make-on-demand:BBV-36630260
Enamine BB Make on Demand:BBV-36630260

Mwt: 241.296
xLogP: 0.39
Charge: 1
RotBond: 4
Protomers: 1
Contact: 2
ES: -29.41 VdW: -14.83
Desolv: p=10.19, ap=-0.46

72
36274047
-34.49

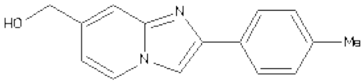

Not for sale

Mwt: 238.29
xLogP: 2.71
Charge: 0
RotBond: 2
Protomers: 2
Contact: 3
ES: -24.52 VdW: -15.49
Desolv: p=6.70, ap=-1.18

73
96963138
-34.48

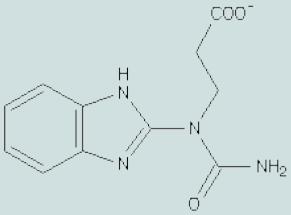

Not for sale

Mwt: 247.234
xLogP: 0.64
Charge: -1
RotBond: 4
Protomers: 2
Contact: 2
ES: -21.27 VdW: -16.29
Desolv: p=3.86, ap=-0.78

74

Molport:MolPort-019-848-329

Mwt: 245.246

41121585
-34.44

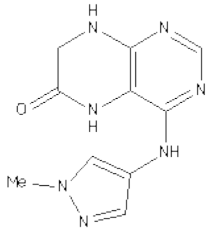

eMolecules:30314257
Princeton BioMolecular Research:OSSL_317614

xLogP: 0.04
Charge: 0
RotBond: 2
Protomers: 2
Contact: 2
ES: -31.75 VdW: -14.22
Desolv: p=11.87, ap=-0.34

75
50070279
-34.44

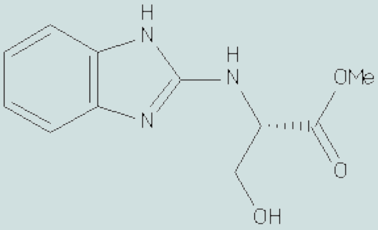

UORSY BB Make-on-demand:BBV-36665525
Enamine BB Make on Demand:BBV-36665525

Mwt: 236.251
xLogP: 0.82
Charge: 1
RotBond: 5
Protomers: 2
Contact: 2
ES: -26.45 VdW: -15.84
Desolv: p=9.12, ap=-1.27

76
13228203
-34.42

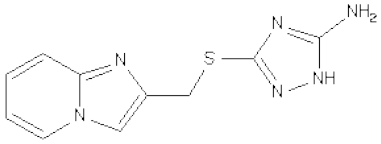

eMolecules:33296923
UORSY BB Make-on-demand:BBV-14845499
Enamine:Z18356137
Mcule:MCULE-8963991570
Enamine BB Make on Demand:BBV-14845499
Mcule Make-on-demand:MCULE-8963991570

Mwt: 247.307
xLogP: 1.31
Charge: 1
RotBond: 3
Protomers: 2
Contact: 2
ES: -29.58 VdW: -13.09
Desolv: p=10.23, ap=-1.98

77
49467322
-34.4

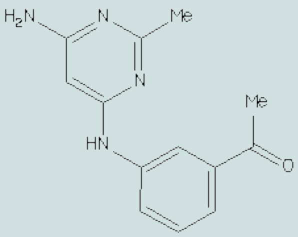

Zylexa Pharma BB:ZP-BB-CD012254
ChemDiv BB Make-on-demand:BB52-2460

Mwt: 243.29
xLogP: 1.92
Charge: 1
RotBond: 3
Protomers: 2
Contact: 2
ES: -28.80 VdW: -15.24
Desolv: p=10.27, ap=-0.63

78
72115186
-34.38

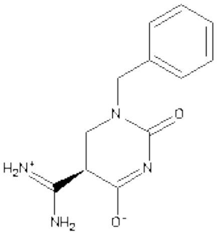

KeyOrganics:11E-928
AKOS (make-on-demand):AKOS015991846

Mwt: 247.278
xLogP: 0.59
Charge: 1
RotBond: 3
Protomers: 2
Contact: 3
ES: -29.80 VdW: -12.69
Desolv: p=8.47, ap=-0.36

79
9927129
-34.36

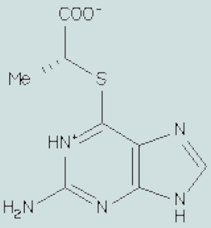

Sigma Aldrich (Building Blocks):A3129|SIGMA
BOC Sciences BB:102082-90-6

Mwt: 237.244
xLogP: -0.04
Charge: -2
RotBond: 3
Protomers: 2
Contact: 2
ES: -25.84 VdW: -11.86
Desolv: p=4.31, ap=-0.97

80
74296762
-34.33

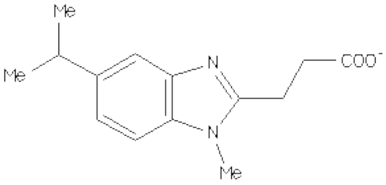

Not for sale

Mwt: 245.302
xLogP: 2.79
Charge: -1
RotBond: 4
Protomers: 2
Contact: 3
ES: -22.72 VdW: -14.09
Desolv: p=4.55, ap=-2.08

81
22264018
-34.32

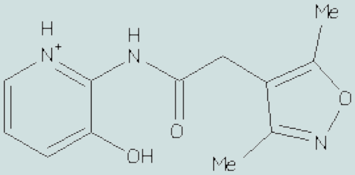

eMolecules:31861936
Enamine:Z185400478

Mwt: 248.262
xLogP: 0.61
Charge: 1
RotBond: 3
Protomers: 4
Contact: 2
ES: -33.37 VdW: -13.14
Desolv: p=12.30, ap=-0.12

82
36780675
-34.32

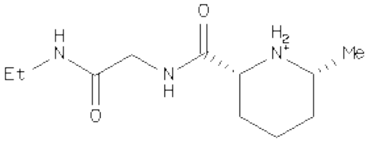

UORSY BB Make-on-demand:BBV-25194676
Enamine BB Make on Demand:BBV-25194676

Mwt: 228.316
xLogP: 0.41
Charge: 1
RotBond: 4
Protomers: 2
Contact: 2
ES: -39.85 VdW: -3.89
Desolv: p=9.39, ap=0.03

83
13821046
-34.31

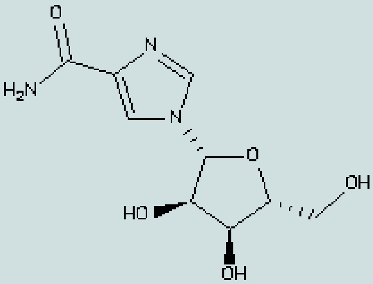

BioSynth:Q-201670

Mwt: 243.219
xLogP: -2.10
Charge: 0
RotBond: 3
Protomers: 2
Contact: 5
ES: -42.09 VdW: -7.28
Desolv: p=13.87, ap=1.17

84

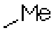

UORSY BB Make-on-demand:BBV-15871824

Mwt: 215.256

37207053
-34.31

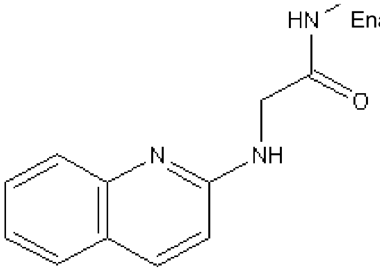

Enamine BB Make on Demand:BBV-15871824

xLogP: 1.75
Charge: 0
RotBond: 3
Protomers: 2
Contact: 2
ES: -26.27 VdW: -17.49
Desolv: p=10.25, ap=-0.80

85
62621300
-34.31

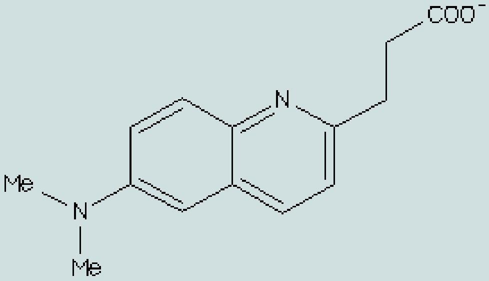

Not for sale

Mwt: 244.294
xLogP: 1.79
Charge: 0
RotBond: 4
Protomers: 2
Contact: 3
ES: -22.53 VdW: -15.51
Desolv: p=5.44, ap=-1.71

86
83285204
-34.31

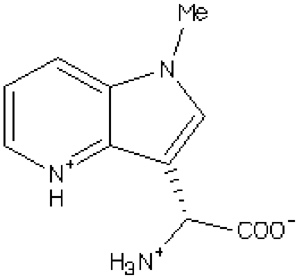

Not for sale

Mwt: 206.225
xLogP: -2.01
Charge: 1
RotBond: 2
Protomers: 3
Contact: 2
ES: -41.92 VdW: -7.09
Desolv: p=15.58, ap=-0.88

87
36274069
-34.3

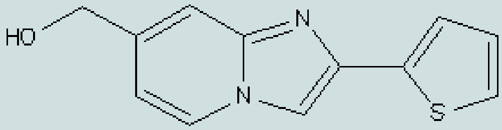

Not for sale

Mwt: 230.292
xLogP: 2.05
Charge: 0
RotBond: 2
Protomers: 2
Contact: 3
ES: -26.52 VdW: -14.36
Desolv: p=7.98, ap=-1.40

88
18286095
-34.29

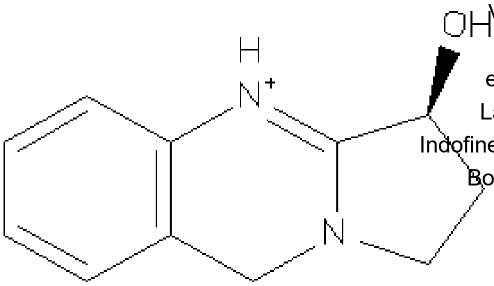

Vitas-M BB:BBL005530
Vitas-M:STK030150
Indofine:NR-012
eMolecules:4368780
Labotest:LT02196378
Indofine Natural Products:NR-012
Bosche Scientific:P6480
Toslab:807657
...

Mwt: 188.23
xLogP: 1.04
Charge: 0
RotBond: 0
Protomers: 3
Contact: 2
ES: -28.16 VdW: -14.75
Desolv: p=9.63, ap=-1.02

89
5685894
-34.28

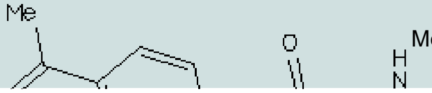

Asinex:BAS04080754
TimTec:ST50274148
Labotest:LT00012293
Molport:MolPort-001-995-052
AKOS:AKOS000625090

Mwt: 230.227
xLogP: 0.29
Charge: 0
RotBond: 3
Protomers: 3

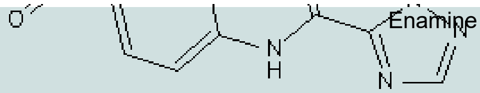

Enamine BB Make on Demand:BBV-43217521

Contact: 2
ES: -24.08 VdW: -11.00
Desolv: p=1.52, ap=-0.72

90
82607742
-34.28

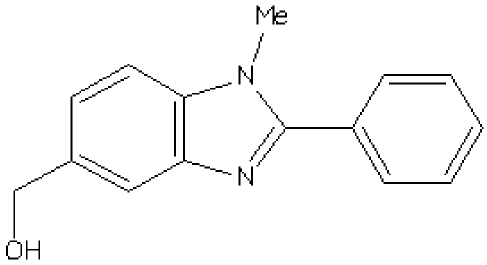

Not for sale

Mwt: 238.29
xLogP: 2.93
Charge: 0
RotBond: 2
Protomers: 2
Contact: 3
ES: -24.87 VdW: -16.08
Desolv: p=7.71, ap=-1.05

PDB

91
44263725
-34.26

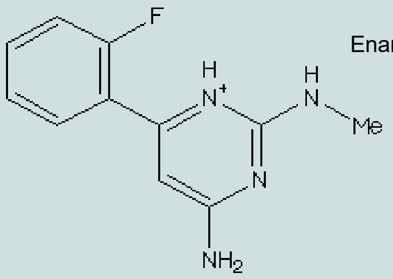

UORSY BB Make-on-demand:BBV-33453408
Enamine BB Make on Demand:BBV-33453408

Mwt: 219.243
xLogP: 2.22
Charge: 1
RotBond: 2
Protomers: 2
Contact: 3
ES: -22.87 VdW: -17.66
Desolv: p=7.70, ap=-1.43

92
96327349
-34.23

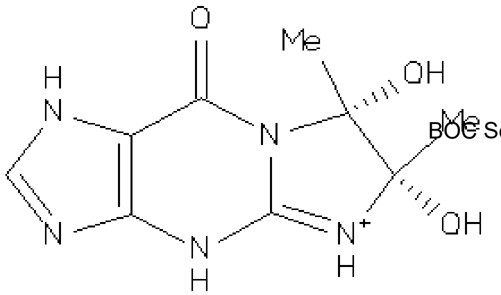

BOC Sciences BB:171813-07-3

Mwt: 238.227
xLogP: 0.08
Charge: 1
RotBond: 0
Protomers: 4
Contact: 3
ES: -34.51 VdW: -12.43
Desolv: p=11.89, ap=0.82

93
40706948
-34.2

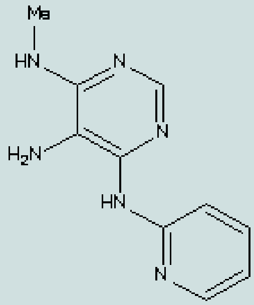

Innovapharm Make-on-Demand:VT-00319699

Mwt: 216.248
xLogP: 0.84
Charge: 0
RotBond: 3
Protomers: 4
Contact: 3
ES: -26.57 VdW: -13.33
Desolv: p=6.98, ap=-1.28

94
40707371
-34.17

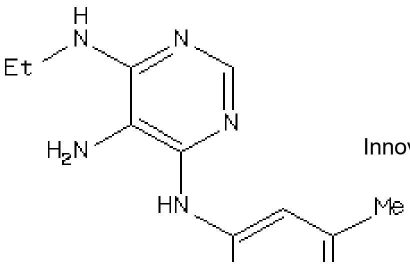

Innovapharm Make-on-Demand:VT-00319906

Mwt: 243.314
xLogP: 2.54
Charge: 0
RotBond: 4
Protomers: 2
Contact: 3
ES: -28.08 VdW: -14.33
Desolv: p=8.85, ap=-0.61

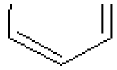

95
83303318
-34.17

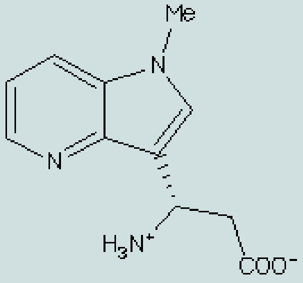

Not for sale

Mwt: 219.244
xLogP: -1.74
Charge: 0
RotBond: 3
Protomers: 3
Contact: 2
ES: -40.23 VdW: -10.16
Desolv: p=17.29, ap=-1.07

96
39292170
-34.16

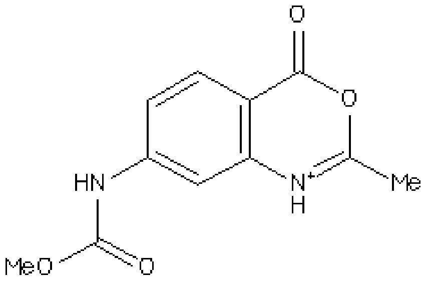

AKOS (make-on-demand):AKOS000279597

Mwt: 235.219
xLogP: 1.19
Charge: 1
RotBond: 2
Protomers: 1
Contact: 2
ES: -32.19 VdW: -12.31
Desolv: p=10.42, ap=-0.08

97
36242920
-34.15

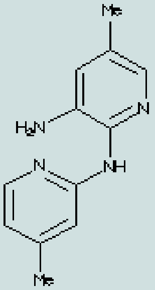

Not for sale

Mwt: 214.272
xLogP: 2.40
Charge: 0
RotBond: 2
Protomers: 4
Contact: 2
ES: -26.16 VdW: -13.52
Desolv: p=6.32, ap=-0.79

98
19775847
-34.13

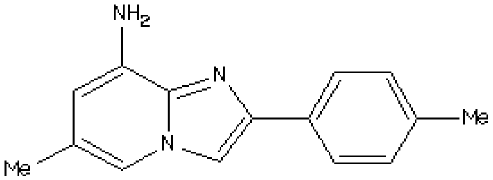

Not for sale

Mwt: 237.306
xLogP: 3.23
Charge: 0
RotBond: 1
Protomers: 2
Contact: 1
ES: -23.24 VdW: -16.91
Desolv: p=7.98, ap=-1.95

99
14451068
-34.11

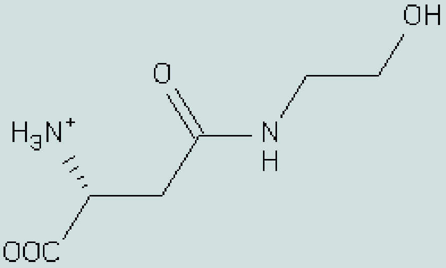

Not for sale

Mwt: 176.172
xLogP: -3.07
Charge: 0
RotBond: 5
Protomers: 1
Contact: 2
ES: -37.87 VdW: -4.85
Desolv: p=7.16, ap=1.44

100
37996520
-34.11

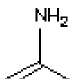

Mwt: 243.287
xLogP: -1.06
Charge: 1

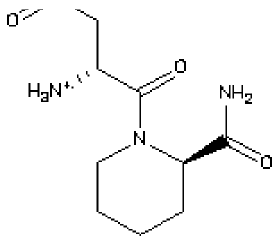

Enamine BB Make on Demand:BBV-32274627

RotBond: 4
Protomers: 2
Contact: 2
ES: -45.02 VdW: -10.06
Desolv: p=19.30, ap=1.68

☐ 101
75947792
-34.09

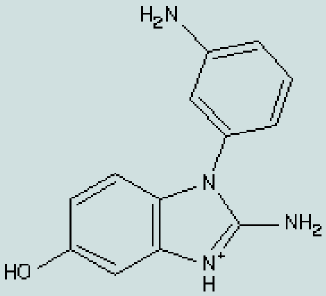

Not for sale

Mwt: 241.274
xLogP: 1.34
Charge: 1
RotBond: 1
Protomers: 3
Contact: 3
ES: -22.23 VdW: -17.18
Desolv: p=6.73, ap=-1.40

☐ 102
82778444
-34.09

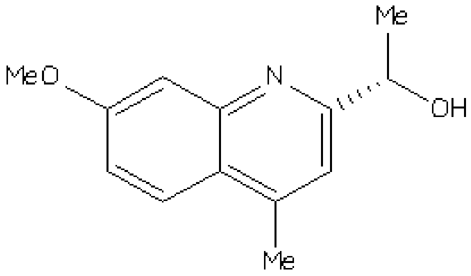

Not for sale

Mwt: 217.268
xLogP: 2.68
Charge: 0
RotBond: 2
Protomers: 2
Contact: 3
ES: -27.30 VdW: -13.81
Desolv: p=7.45, ap=-0.43

☐ 103
41287742
-34.08

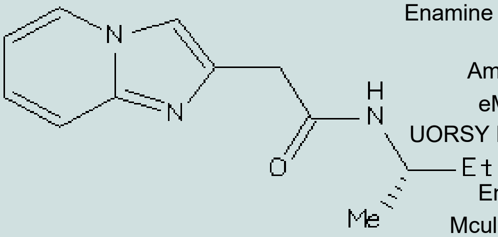

Molport:MolPort-008-928-220
Enamine BB Make on Demand:BBV-39269827
Ambinter:Amb16858700
eMolecules:35691860
UORSY BB Make-on-demand:BBV-39269827
Enamine:Z246711276
Mcule:MCULE-2921400739
Mcule Make-on-demand:MCULE-2921400739
...

Mwt: 231.299
xLogP: 2.03
Charge: 0
RotBond: 4
Protomers: 2
Contact: 2
ES: -29.72 VdW: -14.15
Desolv: p=11.31, ap=-1.52

☐ 104
19903743
-34.07

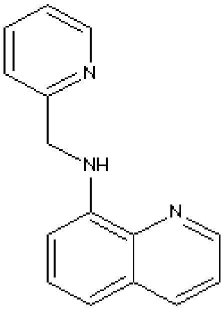

UORSY BB Make-on-demand:BBV-123489
Enamine BB Make on Demand:BBV-123489

Mwt: 235.29
xLogP: 1.98
Charge: 0
RotBond: 3
Protomers: 3
Contact: 2
ES: -27.81 VdW: -16.07
Desolv: p=11.45, ap=-1.64

☐ 105
83283010
-34.07

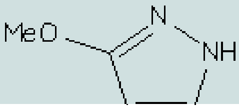

Mwt: 199.21
xLogP: -2.35
Charge: 0
RotBond: 4

	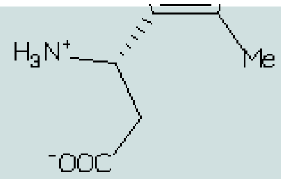	Not for sale	# Protomers: 2<br>Contact: 3<br>ES: -33.22 VdW: -8.49<br>Desolv: p=6.97, ap=0.67
106<br>77018239<br>-34.04	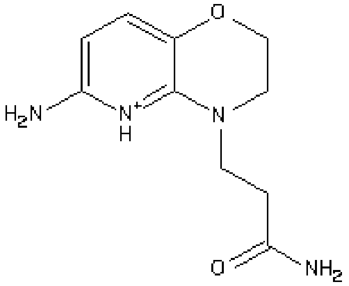	Not for sale	Mwt: 223.256<br>xLogP: -0.16<br>Charge: 1<br>RotBond: 3<br># Protomers: 2<br>Contact: 2<br>ES: -31.34 VdW: -12.92<br>Desolv: p=10.31, ap=-0.08
107<br>82732309<br>-34.03	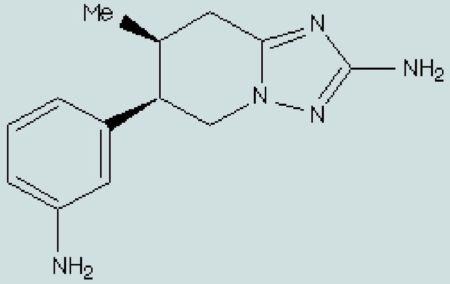	Not for sale	Mwt: 243.314<br>xLogP: 1.27<br>Charge: 0<br>RotBond: 1<br># Protomers: 2<br>Contact: 2<br>ES: -29.77 VdW: -11.86<br>Desolv: p=8.22, ap=-0.62
108<br>8656770<br>-34.02	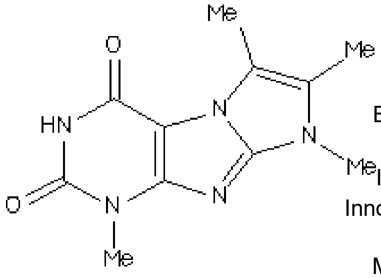	IBScreen:STOCK5S-34277<br>Pharmeks:PHAR143845<br>eMolecules:30346487<br>Princeton BioMolecular BuildingBlocks:PBMR111971<br>eMolecules:4672336<br>Innovapharm:STT-00377636<br>Innovapharm Building Blocks:BBS-00017607<br>Molport:MolPort-002-639-017<br>...	Mwt: 248.266<br>xLogP: -0.14<br>Charge: 1<br>RotBond: 0<br># Protomers: 2<br>Contact: 1<br>ES: -26.02 VdW: -15.83<br>Desolv: p=9.40, ap=-1.57
109<br>5729255<br>-33.98	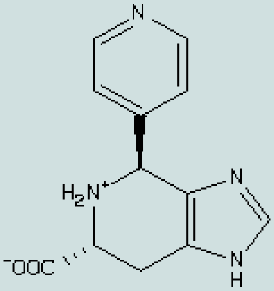	Bosche Scientific:P16649<br>Tyger Building Blocks:P60047<br>Ambinter:Amb2700793<br>Chembo Pharma:KB-243108	Mwt: 244.254<br>xLogP: -1.73<br>Charge: 0<br>RotBond: 2<br># Protomers: 4<br>Contact: 1<br>ES: -27.67 VdW: -11.88<br>Desolv: p=6.23, ap=-0.66
110<br>5927844<br>-33.98	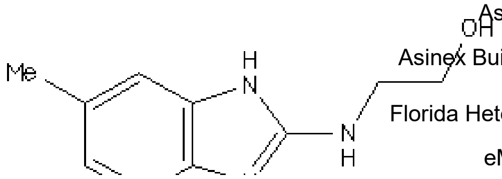	Matrix Scientific:018417<br>ChemBridge BuildingBlocks:5522535<br>Asinex:BAS00619334<br>Asinex Building Blocks:BAS00619334<br>Florida Heterocyclic Compounds:18095<br>eMolecules:1249885	Mwt: 191.234<br>xLogP: 1.40<br>Charge: 0<br>RotBond: 3<br># Protomers: 4<br>Contact: 2

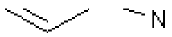

Princeton BioMolecular BuildingBlocks:PBMR023257
Innovapharm BB Make on Demand:BBV-00033241
...

ES: -28.77 VdW: -11.36
Desolv: p=6.72, ap=-0.58

111
13814215
-33.98

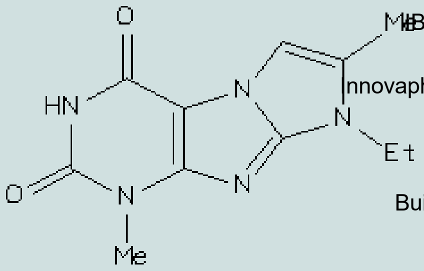

Pharmeks:PHAR128626
IBScreen:STOCK5S-32279
ChemBridge:9074657
Innovapharm BB Make on Demand:BBV-00062540
UORSY:PB237453550
Princeton BioMolecular BuildingBlocks:PBMR111658
eMolecules:30357681
eMolecules:2555907
...

Mwt: 247.258
xLogP: 0.01
Charge: 0
RotBond: 1
Protomers: 2
Contact: 1
ES: -25.44 VdW: -16.50
Desolv: p=9.75, ap=-1.79

112
35203925
-33.97

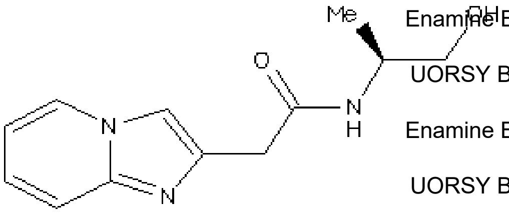

AKOS (make-on-demand):AKOS005832201
Enamine BB Make on Demand:BBV-24880679
UORSY BB Make-on-demand:BBV-24880679
Enamine BB Make on Demand:BBV-40738789
UORSY BB Make-on-demand:BBV-40738789

Mwt: 233.271
xLogP: 0.52
Charge: 0
RotBond: 4
Protomers: 2
Contact: 2
ES: -28.43 VdW: -15.22
Desolv: p=10.64, ap=-0.96

113
74318921
-33.97

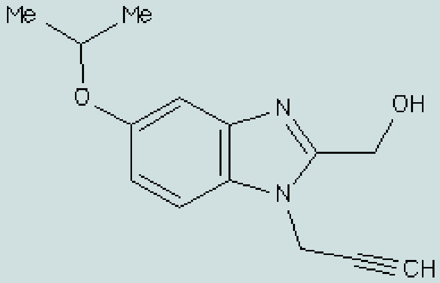

Not for sale

Mwt: 245.302
xLogP: 1.87
Charge: 1
RotBond: 4
Protomers: 2
Contact: 4
ES: -27.84 VdW: -14.85
Desolv: p=9.34, ap=-0.61

114
12541056
-33.96

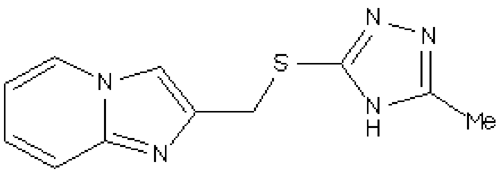

Molport:MolPort-005-594-067
Enamine:Z228112014

Mwt: 246.319
xLogP: 1.59
Charge: 1
RotBond: 3
Protomers: 2
Contact: 2
ES: -29.35 VdW: -14.00
Desolv: p=11.37, ap=-1.98

115
21046778
-33.96

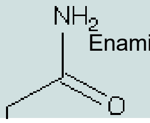

UORSY BB Make-on-demand:BBR-039407
Enamine BB Make on Demand:BBR-039407

Mwt: 201.229
xLogP: 0.87
Charge: 0

	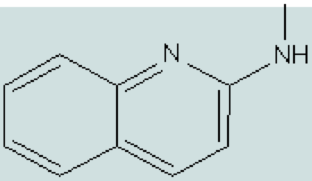		RotBond: 3<br># Protomers: 2<br>Contact: 2<br>ES: -26.76 VdW: -14.73<br>Desolv: p=8.69, ap=-1.17
116<br>77092645<br>-33.95	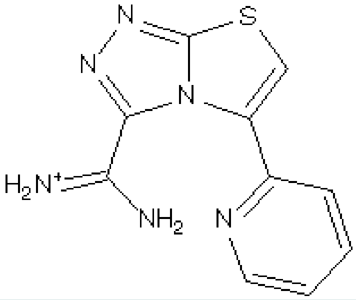	Not for sale	Mwt: 245.291<br>xLogP: -0.48<br>Charge: 1<br>RotBond: 2<br># Protomers: 1<br>Contact: 2<br>ES: -30.42 VdW: -12.48<br>Desolv: p=9.84, ap=-0.89
117<br>5012914<br>-33.93	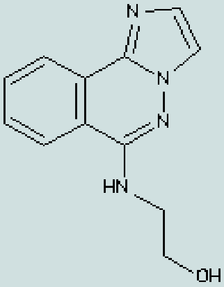	NCI Plated 2007:186385	Mwt: 228.255<br>xLogP: 1.25<br>Charge: 0<br>RotBond: 3<br># Protomers: 2<br>Contact: 2<br>ES: -26.47 VdW: -15.19<br>Desolv: p=8.86, ap=-1.14
118<br>5962745<br>-33.93	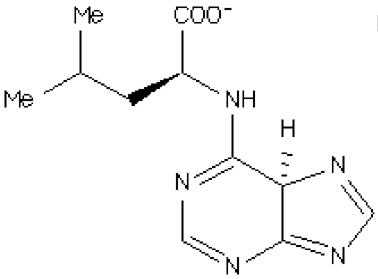	Molport:MolPort-002-507-542<br>Molport:MolPort-005-979-958	Mwt: 249.274<br>xLogP: -1.21<br>Charge: 0<br>RotBond: 5<br># Protomers: 2<br>Contact: 2<br>ES: -29.01 VdW: -9.67<br>Desolv: p=6.20, ap=-1.45
119<br>82731967<br>-33.93	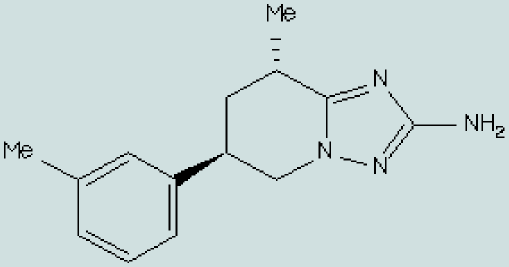	Not for sale	Mwt: 242.326<br>xLogP: 2.50<br>Charge: 0<br>RotBond: 1<br># Protomers: 2<br>Contact: 2<br>ES: -23.51 VdW: -17.59<br>Desolv: p=9.15, ap=-1.99
120<br>4897316<br>-33.92	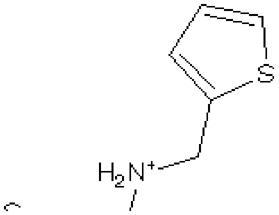	ChemBridge:9150436<br>eMolecules:1491723<br>Specs:AN-465/42246604<br>Asinex:BAS07017869<br>Vitas-M:STK289703<br>Princeton BioMolecular Research:OSSL_007413	Mwt: 224.374<br>xLogP: 2.92<br>Charge: 1<br>RotBond: 4<br># Protomers: 1<br>Contact: 2

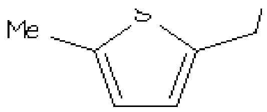

UORSY BB Make-on-demand:BBV-32182354
eMolecules:7716366
...

ES: -43.04 VdW: -4.42
Desolv: p=13.28, ap=0.26

121
57534978
-33.92

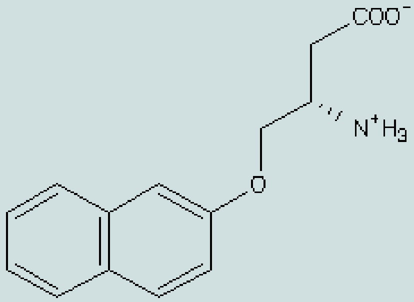

Not for sale

Mwt: 244.27
xLogP: 1.56
Charge: -1
RotBond: 5
Protomers: 2
Contact: 2
ES: -29.26 VdW: -12.44
Desolv: p=8.46, ap=-0.68

122
5830041
-33.88

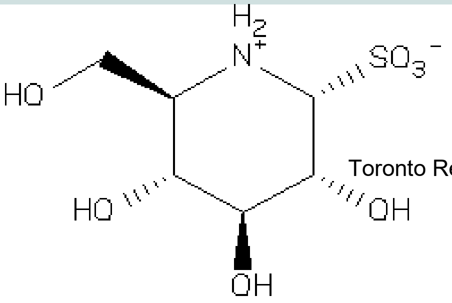

Toronto Research Chemicals:N635000

Mwt: 243.237
xLogP: -4.85
Charge: 0
RotBond: 2
Protomers: 2
Contact: 5
ES: -34.63 VdW: -6.48
Desolv: p=5.12, ap=2.12

123
36730058
-33.88

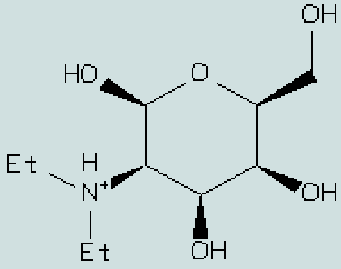

Scientific Exchange (make on demand):F-393651
TimTec:ST45025433
Ambinter:Amb9871388
Princeton BioMolecular BuildingBlocks:PBMR194046
Molport BB:MolPort-006-328-857

Mwt: 236.288
xLogP: -1.45
Charge: 1
RotBond: 4
Protomers: 2
Contact: 7
ES: -34.92 VdW: -6.22
Desolv: p=7.34, ap=-0.08

124
82344891
-33.88

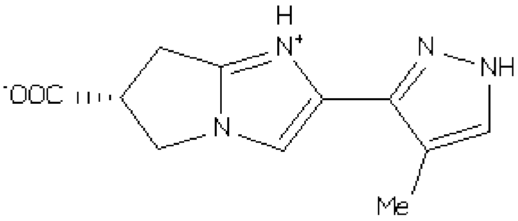

Not for sale

Mwt: 232.243
xLogP: 0.06
Charge: 0
RotBond: 2
Protomers: 4
Contact: 2
ES: -28.79 VdW: -9.48
Desolv: p=5.43, ap=-1.04

125
82948991
-33.88

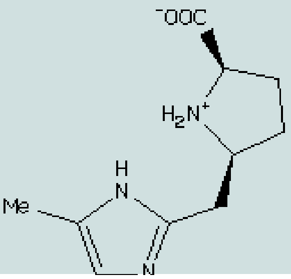

Not for sale

Mwt: 210.257
xLogP: -1.62
Charge: 1
RotBond: 3
Protomers: 2
Contact: 3
ES: -29.55 VdW: -9.34
Desolv: p=5.82, ap=-0.81

PDB

126
76079834

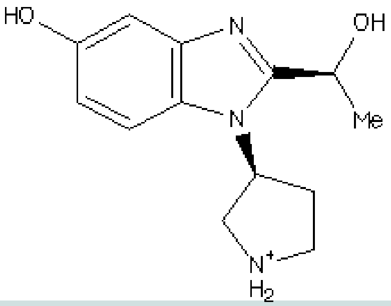

Not for sale

Mwt: 248.306
xLogP: 0.57
Charge: 1
RotBond: 2
Protomers: 4
Contact: 3
ES: -44.89 VdW: -9.55
Desolv: p=19.55, ap=1.01

PDB

127
83341504
-33.87

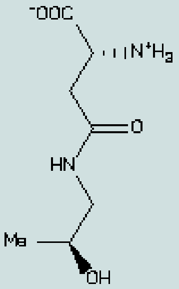

Not for sale

Mwt: 189.191
xLogP: -2.70
Charge: -1
RotBond: 5
Protomers: 2
Contact: 2
ES: -37.59 VdW: -4.33
Desolv: p=6.82, ap=1.22

PDB

128
97096519
-33.86

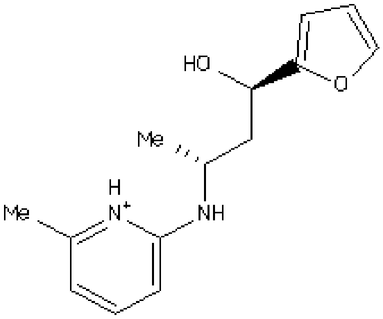

Not for sale

Mwt: 247.318
xLogP: 1.62
Charge: 1
RotBond: 5
Protomers: 2
Contact: 2
ES: -30.29 VdW: -13.22
Desolv: p=10.24, ap=-0.60

PDB

129
4089298
-33.84

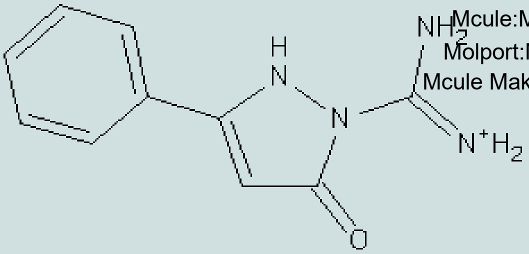

KeyOrganics:1X-0220
Mcule:MCULE-6244941181
Molport:MolPort-002-860-974
Mcule Make-on-demand:MCULE-6244941181

Mwt: 203.225
xLogP: 0.58
Charge: 1
RotBond: 2
Protomers: 3
Contact: 1
ES: -27.37 VdW: -12.87
Desolv: p=7.71, ap=-1.31

PDB

130
36222597
-33.83

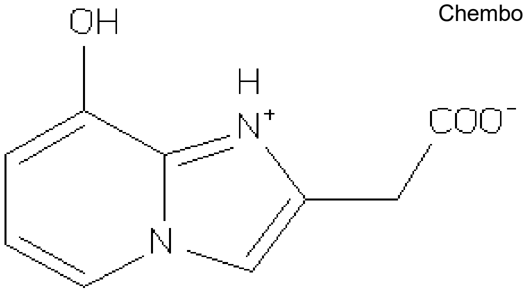

Sphinx Make-on-demand:IM-28-0044
Chembo Pharma:KB-273215

Mwt: 192.174
xLogP: 0.70
Charge: 0
RotBond: 2
Protomers: 2
Contact: 3
ES: -25.98 VdW: -13.92
Desolv: p=7.16, ap=-1.09

PDB

131
76079833
-33.83

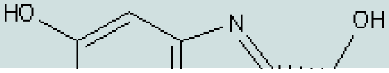

Mwt: 248.306
xLogP: 0.57
Charge: 1

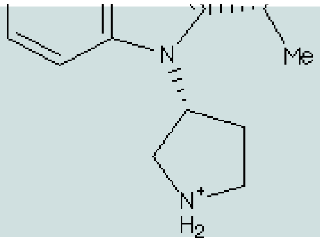

Not for sale

RotBond: 2
Protomers: 4
Contact: 3
ES: -37.87 VdW: -9.06
Desolv: p=13.04, ap=0.07

PDB

132
6007228
-33.82

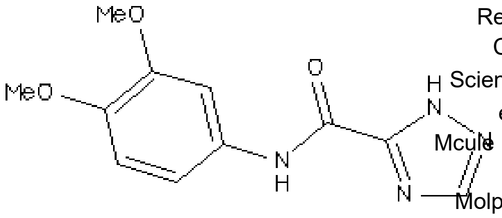

Chemical Block:A3249/0138037
Princeton BioMolecular Research:OSSL_299555
ChemBridge:7964469
Scientific Exchange:M-172004
eMolecules:2046032
Mcule Make-on-demand:MCULE-3280382285
Molport:MolPort-001-572-916
Scientific Exchange (make on demand):M-172004
...

Mwt: 247.234
xLogP: 0.03
Charge: -1
RotBond: 4
Protomers: 3
Contact: 4
ES: -23.62 VdW: -11.26
Desolv: p=1.51, ap=-0.45

PDB

133
83364580
-33.82

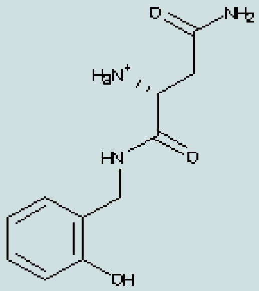

Not for sale

Mwt: 236.251
xLogP: -0.15
Charge: -1
RotBond: 5
Protomers: 3
Contact: 3
ES: -34.34 VdW: -10.58
Desolv: p=8.87, ap=2.23

PDB

134
82222640
-33.79

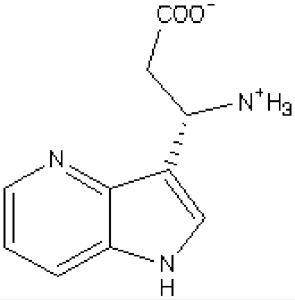

Not for sale

Mwt: 206.225
xLogP: -1.81
Charge: 1
RotBond: 3
Protomers: 3
Contact: 2
ES: -41.08 VdW: -10.12
Desolv: p=17.88, ap=-0.47

PDB

135
82299630
-33.79

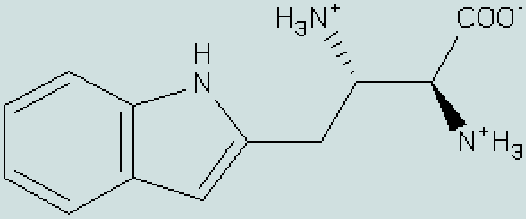

Not for sale

Mwt: 234.279
xLogP: -1.91
Charge: 1
RotBond: 4
Protomers: 4
Contact: 2
ES: -29.96 VdW: -10.67
Desolv: p=6.82, ap=0.03

PDB

136
37815452
-33.77

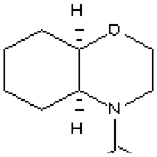

UORSY BB Make-on-demand:BBV-32159404
Enamine BB Make on Demand:BBV-32159404

Mwt: 241.355
xLogP: 1.36
Charge: 1
RotBond: 3

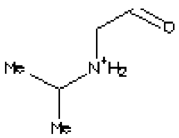

Protomers: 2
Contact: 2
ES: -42.44 VdW: -5.85
Desolv: p=15.45, ap=-0.94

PDB

137
36896069
-33.76

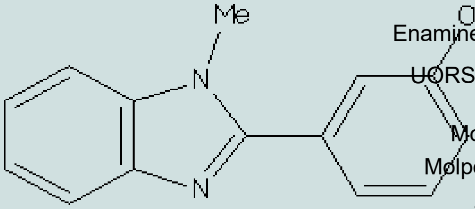

eMolecules:32442031
Molport:MolPort-011-526-629
Enamine Building Blocks:EN300-68590
UORSY BB Make-on-demand:BBV-2085254
Mcule:MCULE-6233860570
Molport BB:MolPort-011-526-629
Ambinter:Amb8836674
Enamine BB Make on Demand:BBV-2085254
...

Mwt: 224.263
xLogP: 3.11
Charge: 0
RotBond: 1
Protomers: 2
Contact: 3
ES: -26.44 VdW: -13.31
Desolv: p=6.93, ap=-0.94

PDB

138
42195959
-33.76

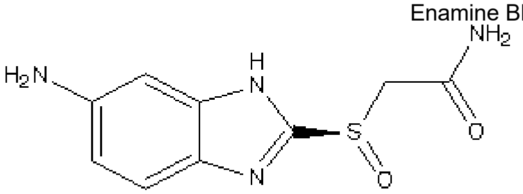

UORSY BB Make-on-demand:BBV-32796090
Enamine BB Make on Demand:BBV-32796090

Mwt: 238.272
xLogP: -1.46
Charge: 0
RotBond: 3
Protomers: 1
Contact: 2
ES: -25.93 VdW: -14.79
Desolv: p=6.32, ap=0.65

PDB

139
42561224
-33.75

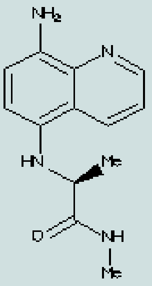

UORSY BB Make-on-demand:BBV-32905344
Enamine BB Make on Demand:BBV-32905344

Mwt: 244.298
xLogP: 1.59
Charge: 0
RotBond: 3
Protomers: 2
Contact: 2
ES: -28.40 VdW: -15.15
Desolv: p=10.14, ap=-0.34

PDB

140
37434689
-33.74

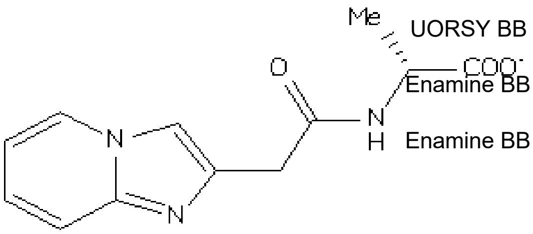

UORSY BB Make-on-demand:BBV-33206790
UORSY BB Make-on-demand:BBV-5750157
Enamine BB Make on Demand:BBV-5750157
Enamine BB Make on Demand:BBV-33206790

Mwt: 246.246
xLogP: -1.06
Charge: -1
RotBond: 4
Protomers: 2
Contact: 2
ES: -26.21 VdW: -14.41
Desolv: p=8.24, ap=-1.36

PDB

141

Mwt: 235.267

76680625
-33.72

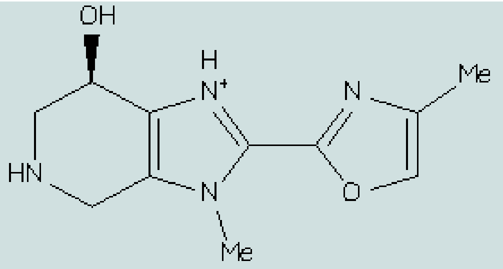

Not for sale

xLogP: -0.17
Charge: 1
RotBond: 1
Protomers: 3
Contact: 3
ES: -28.93 VdW: -13.16
Desolv: p=8.20, ap=0.17

PDB

142
19593947
-33.71

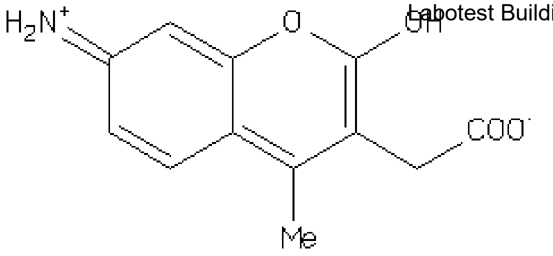

Labotest:LT00645544
Labotest Building Blocks:LT03510469

Mwt: 233.223
xLogP: 0.40
Charge: 0
RotBond: 2
Protomers: 1
Contact: 3
ES: -25.32 VdW: -12.49
Desolv: p=4.87, ap=-0.76

PDB

143
33852720
-33.71

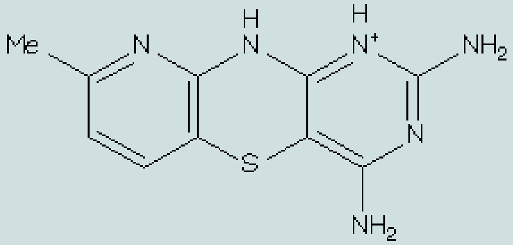

Not for sale

Mwt: 247.307
xLogP: 0.97
Charge: 1
RotBond: 0
Protomers: 3
Contact: 1
ES: -26.30 VdW: -15.94
Desolv: p=9.35, ap=-0.81

PDB

144
75947820
-33.71

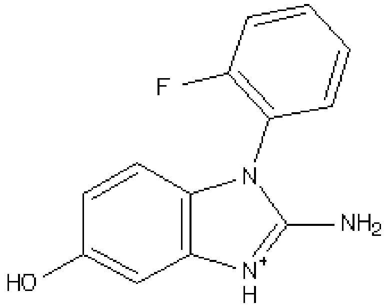

Not for sale

Mwt: 244.249
xLogP: 2.40
Charge: 1
RotBond: 1
Protomers: 3
Contact: 3
ES: -23.55 VdW: -16.34
Desolv: p=7.71, ap=-1.53

PDB

145
83346988
-33.71

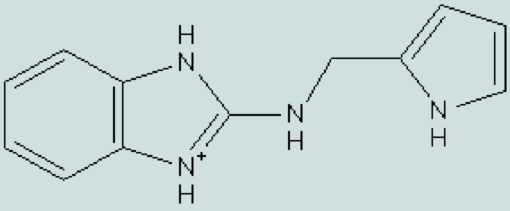

Not for sale

Mwt: 213.264
xLogP: 2.16
Charge: 1
RotBond: 3
Protomers: 2
Contact: 2
ES: -27.16 VdW: -13.90
Desolv: p=8.69, ap=-1.33

PDB

146
95923362
-33.71

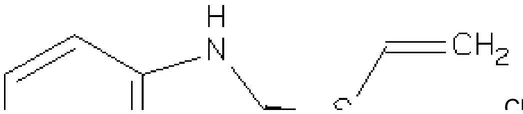

Chemstep:12250

Mwt: 193.251
xLogP: 1.06
Charge: 1
RotBond: 2
Protomers: 2

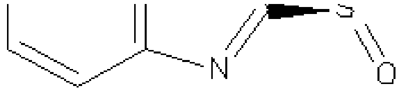

[illegible]

Contact: 2
ES: -29.93 VdW: -14.00
Desolv: p=11.06, ap=-0.84

PDB
147
1395785
-33.7

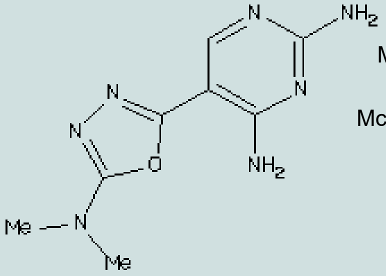

KeyOrganics:7B-071
Mcule:MCULE-2674464689
Molport:MolPort-002-876-167
Ambinter:Amb2433215
Mcule Make-on-demand:MCULE-2674464689

Mwt: 221.224
xLogP: -0.34
Charge: 0
RotBond: 2
Protomers: 3
Contact: 2
ES: -28.31 VdW: -16.16
Desolv: p=9.58, ap=1.19

PDB
148
35200954
-33.7

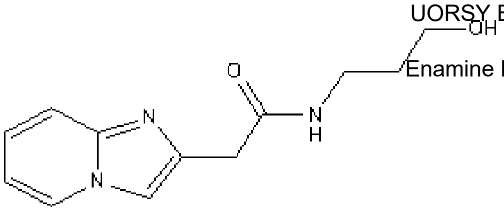

AKOS (make-on-demand):AKOS005832374
UORSY BB Make-on-demand:BBV-24879629
Enamine BB Make on Demand:BBV-24879629

Mwt: 233.271
xLogP: 0.46
Charge: 0
RotBond: 5
Protomers: 2
Contact: 2
ES: -31.40 VdW: -13.32
Desolv: p=11.62, ap=-0.59

PDB
149
71781362
-33.7

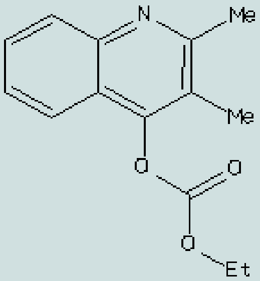

Not for sale

Mwt: 245.278
xLogP: 3.27
Charge: 0
RotBond: 4
Protomers: 2
Contact: 2
ES: -23.12 VdW: -15.80
Desolv: p=7.21, ap=-1.99

PDB
150
74461004
-33.68

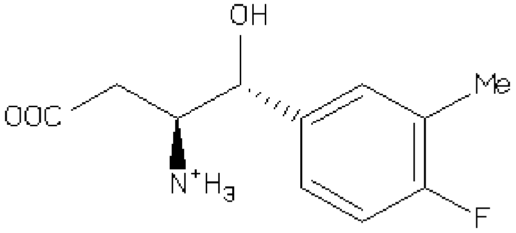

Not for sale

Mwt: 227.235
xLogP: 0.05
Charge: 0
RotBond: 4
Protomers: 2
Contact: 2
ES: -34.43 VdW: -9.20
Desolv: p=10.02, ap=-0.07

PDB
151
82974756
-33.68

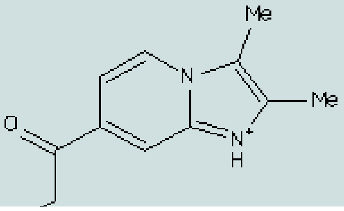

Not for sale

Mwt: 218.28
xLogP: 1.00
Charge: 1
RotBond: 3
Protomers: 3
Contact: 2
ES: -35.61 VdW: -9.69

PDB	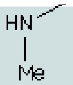		Desolv: p=12.32, ap=-0.71
152<br>40779100<br>-33.65<br>PDB	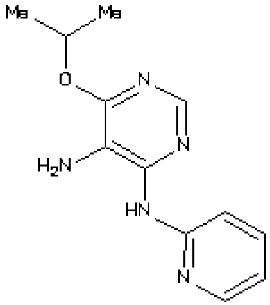	Innovapharm Make-on-Demand:VT-00331684	Mwt: 245.286<br>xLogP: 1.78<br>Charge: 0<br>RotBond: 4<br># Protomers: 3<br>Contact: 2<br>ES: -25.30 VdW: -17.82<br>Desolv: p=10.38, ap=-0.92
153<br>50864502<br>-33.65<br>PDB	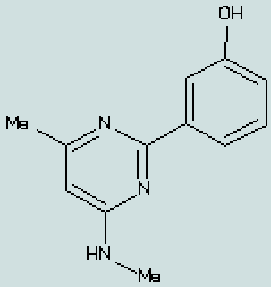	Enamine BB Make on Demand:BBV-34303435	Mwt: 215.256<br>xLogP: 2.04<br>Charge: 0<br>RotBond: 2<br># Protomers: 2<br>Contact: 3<br>ES: -25.88 VdW: -15.09<br>Desolv: p=7.63, ap=-0.31
154<br>75870483<br>-33.64<br>PDB	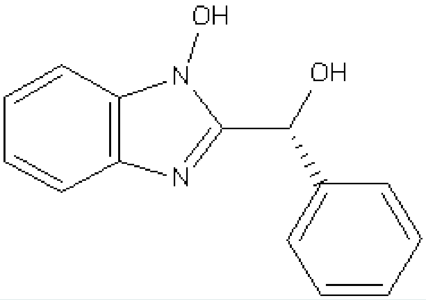	Not for sale	Mwt: 240.262<br>xLogP: 2.21<br>Charge: 0<br>RotBond: 2<br># Protomers: 3<br>Contact: 3<br>ES: -29.91 VdW: -14.10<br>Desolv: p=11.43, ap=-1.07
155<br>83051224<br>-33.64<br>PDB	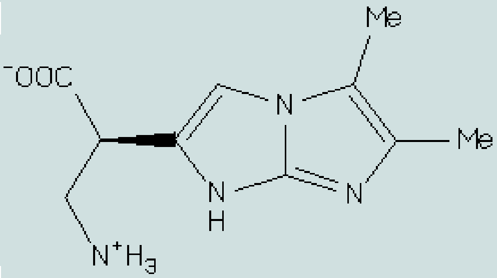	Not for sale	Mwt: 221.24<br>xLogP: -1.01<br>Charge: -1<br>RotBond: 3<br># Protomers: 3<br>Contact: 2<br>ES: -30.62 VdW: -13.07<br>Desolv: p=11.63, ap=-1.58
156<br>20052857<br>-33.61<br>PDB	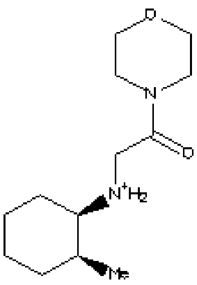	UORSY BB Make-on-demand:BBV-150185<br>Enamine BB Make on Demand:BBV-150185	Mwt: 241.355<br>xLogP: 1.44<br>Charge: 1<br>RotBond: 3<br># Protomers: 2<br>Contact: 2<br>ES: -42.52 VdW: -3.69<br>Desolv: p=13.06, ap=-0.46

157
76079799
-33.61

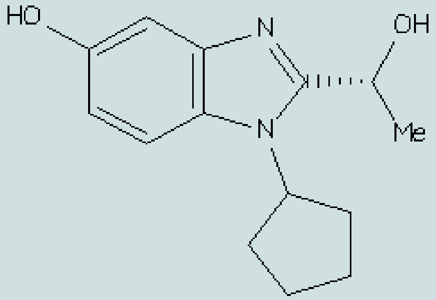

Not for sale

Mwt: 246.31
xLogP: 2.06
Charge: 0
RotBond: 2
Protomers: 3
Contact: 3
ES: -31.41 VdW: -9.83
Desolv: p=7.73, ap=-0.10

PDB

158
97813733
-33.61

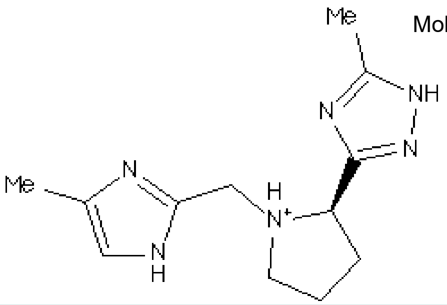

ChemBridge:96148732
Molport:MolPort-030-064-032

Mwt: 247.326
xLogP: -0.31
Charge: 1
RotBond: 3
Protomers: 4
Contact: 3
ES: -36.90 VdW: -7.35
Desolv: p=11.30, ap=-0.67

PDB

159
52006029
-33.58

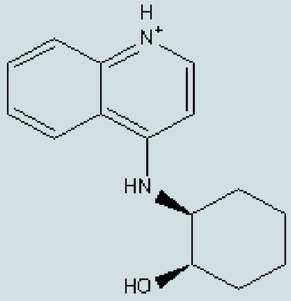

UORSY BB Make-on-demand:BBV-34243694
Enamine BB Make on Demand:BBV-34243694

Mwt: 243.33
xLogP: 3.45
Charge: 1
RotBond: 2
Protomers: 1
Contact: 2
ES: -25.77 VdW: -13.48
Desolv: p=6.30, ap=-0.63

PDB

160
76079832
-33.58

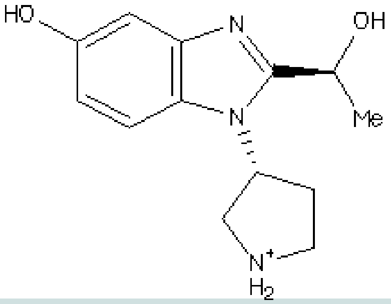

Not for sale

Mwt: 248.306
xLogP: 0.57
Charge: 1
RotBond: 2
Protomers: 4
Contact: 4
ES: -37.67 VdW: -5.07
Desolv: p=8.51, ap=0.66

PDB

161
2326910
-33.57

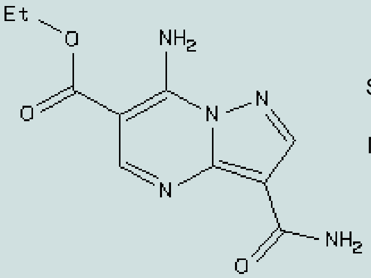

Scientific Exchange:R-099491
eMolecules:2520446
ChemBridge:7983899
Scientific Exchange (make on demand):R-099491
Molport:MolPort-001-495-440
Vitas-M:STK851352
IBScreen:STOCK5S-48253
Vitas-M:STL312474
...

Mwt: 249.23
xLogP: -2.14
Charge: 0
RotBond: 4
Protomers: 2
Contact: 4
ES: -32.11 VdW: -5.36
Desolv: p=3.67, ap=0.22

PDB

162
41147482

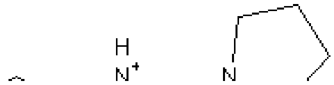

Princeton BioMolecular Research:OSSL_323099
Mcule:MCULE-9200057031

Mwt: 243.334
xLogP: 3.26

-33.57

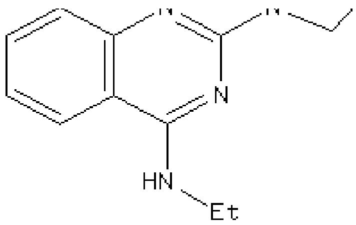

Charge: 1
RotBond: 3
Protomers: 2
Contact: 1
ES: -21.17 VdW: -16.60
Desolv: p=6.57, ap=-2.38

PDB

163
13353957
-33.56

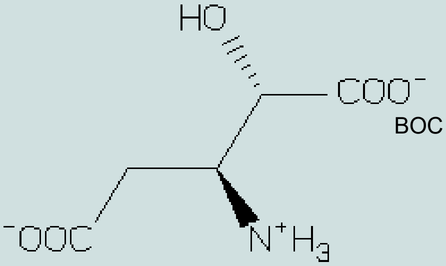

BOC Sciences BB:174320-28-6

Mwt: 162.121
xLogP: -2.71
Charge: -1
RotBond: 4
Protomers: 1
Contact: 2
ES: -31.37 VdW: -2.59
Desolv: p=-0.76, ap=1.17

PDB

164
26474093
-33.54

Innovapharm BB Make on Demand:BBV-00033271
Molport BB:MolPort-007-985-442
Ambinter:Amb9835344

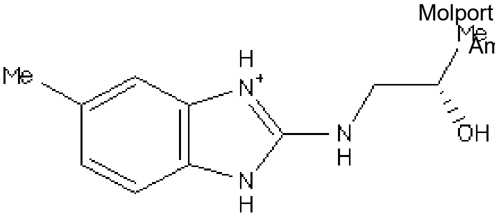

Mwt: 205.261
xLogP: 1.76
Charge: 0
RotBond: 3
Protomers: 2
Contact: 2
ES: -28.92 VdW: -11.22
Desolv: p=7.26, ap=-0.66

PDB

165
72465443
-33.54

Innovapharm Make-on-Demand:VT-00673260
Mcule:MCULE-9183369145

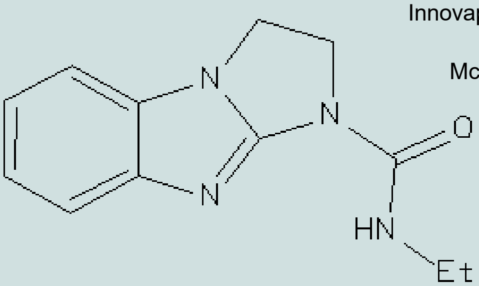

Mwt: 230.271
xLogP: 1.81
Charge: 0
RotBond: 1
Protomers: 2
Contact: 2
ES: -25.01 VdW: -13.91
Desolv: p=6.98, ap=-1.59

PDB

166
82895774
-33.54

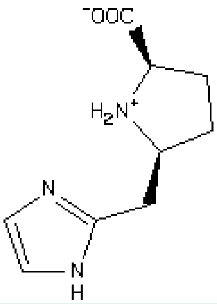

Not for sale

Mwt: 195.222
xLogP: -1.84
Charge: 0
RotBond: 3
Protomers: 3
Contact: 3
ES: -30.90 VdW: -8.07
Desolv: p=6.18, ap=-0.74

PDB

167
36782679
-33.53

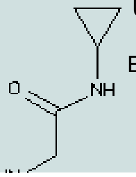

UORSY BB Make-on-demand:BBV-25196116
Enamine BB Make on Demand:BBV-25196116

Mwt: 240.327
xLogP: 0.40
Charge: 1
RotBond: 4

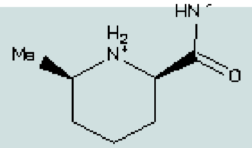

Protomers: 2
Contact: 2
ES: -37.31 VdW: -5.17
Desolv: p=9.27, ap=-0.33

PDB
168
83014145
-33.53

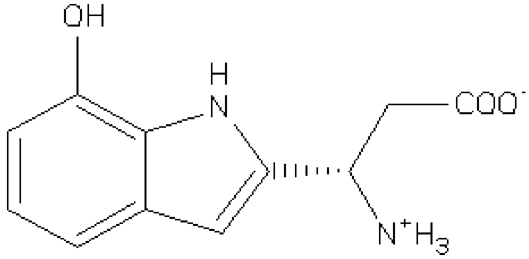

Not for sale

Mwt: 219.22
xLogP: -0.86
Charge: -1
RotBond: 3
Protomers: 2
Contact: 3
ES: -31.06 VdW: -11.55
Desolv: p=8.70, ap=0.37

PDB
169
5595144
-33.51

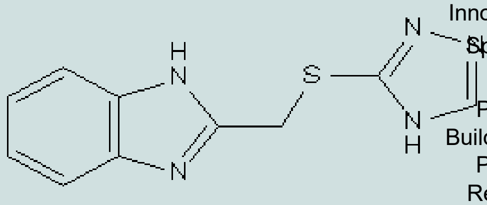

Innovapharm BB Make on Demand:BBV-00026240
Innovapharm:STT-00342504
Specs:AG-690/14006203
Vitas-M:STK395301
Princeton BioMolecular BuildingBlocks:PBMR009167
Princeton BioMolecular Research:OSSL_148324
Molport:MolPort-002-335-677
ZereneX Building Blocks:E/9120295
...

Mwt: 231.284
xLogP: 1.50
Charge: 0
RotBond: 3
Protomers: 1
Contact: 2
ES: -21.11 VdW: -14.83
Desolv: p=3.51, ap=-1.08

PDB
170
12732849
-33.51

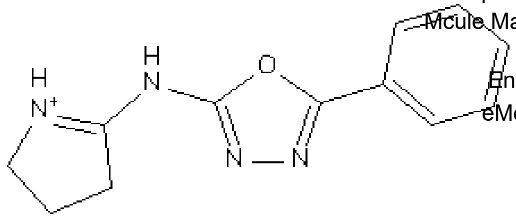

Mcule:MCULE-7626724151
Molport:MolPort-005-857-675
Mcule Make-on-demand:MCULE-7626724151
Enamine:Z55928352
eMolecules:45570008

Mwt: 229.263
xLogP: 2.12
Charge: 1
RotBond: 3
Protomers: 1
Contact: 2
ES: -25.95 VdW: -16.61
Desolv: p=9.62, ap=-0.57

PDB
171
83076120
-33.51

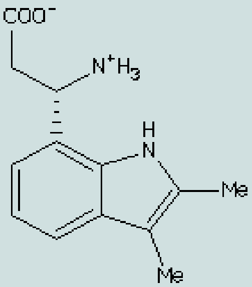

Not for sale

Mwt: 232.283
xLogP: -0.28
Charge: 0
RotBond: 3
Protomers: 1
Contact: 2
ES: -28.52 VdW: -11.73
Desolv: p=7.63, ap=-0.89

PDB
172
35498029
-33.5

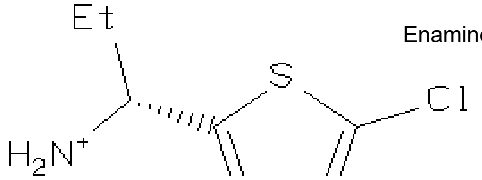

UORSY BB Make-on-demand:BBV-36629118
Enamine BB Make on Demand:BBV-36629118

Mwt: 190.719
xLogP: 3.26
Charge: 1
RotBond: 3
Protomers: 2

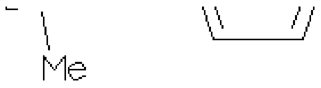

Contact: 2
ES: -43.23 VdW: -2.93
Desolv: p=13.21, ap=-0.55

PDB

173
76163661
-33.48

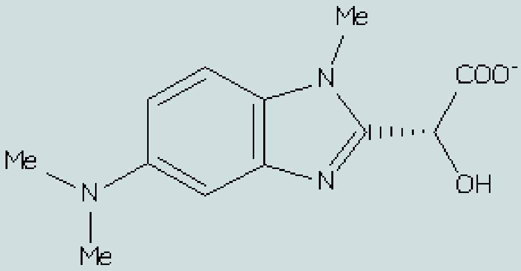

Not for sale

Mwt: 248.262
xLogP: 0.11
Charge: -1
RotBond: 3
Protomers: 2
Contact: 2
ES: -22.84 VdW: -13.45
Desolv: p=3.34, ap=-0.53

PDB

174
89201718
-33.48

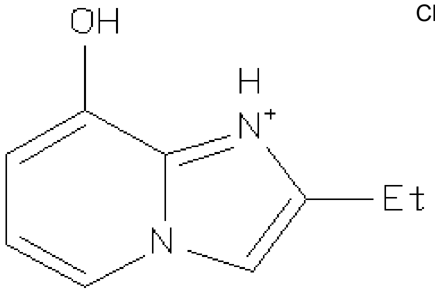

Sphinx Make-on-demand:IM-28-0010
Chembo Pharma:KB-272871

Mwt: 162.192
xLogP: 1.83
Charge: 0
RotBond: 1
Protomers: 2
Contact: 2
ES: -28.71 VdW: -12.78
Desolv: p=9.09, ap=-1.08

PDB

175
76079774
-33.47

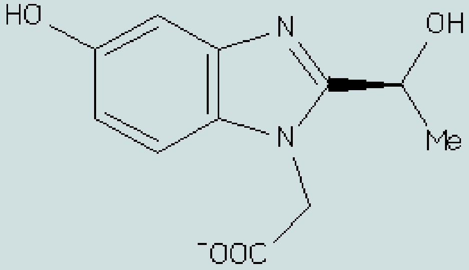

Not for sale

Mwt: 235.219
xLogP: -0.09
Charge: -1
RotBond: 3
Protomers: 3
Contact: 4
ES: -27.10 VdW: -12.16
Desolv: p=5.49, ap=0.29

176

82549114
-33.46

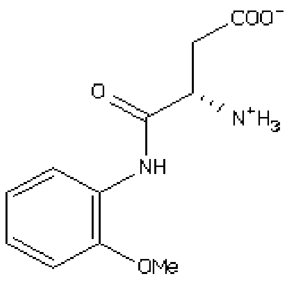

UORSY BB Make-on-demand:BBV-39452543
Enamine BB Make on Demand:BBV-39452543

Mwt: 238.243

xLogP: -0.50

Charge: 0
RotBond: 5
Protomers: 2
Contact: 3
ES: -27.93 VdW: -14.11
Desolv: p=8.08, ap=0.50

177
83014139
-33.46

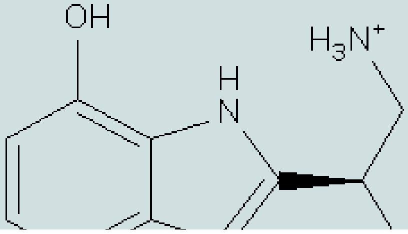

Not for sale

Mwt: 220.228
xLogP: 0.15
Charge: 0
RotBond: 3
Protomers: 2
Contact: 3
ES: -32.52 VdW: -11.15
Desolv: p=9.51, ap=0.69

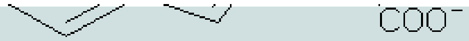

178
75912749
-33.45

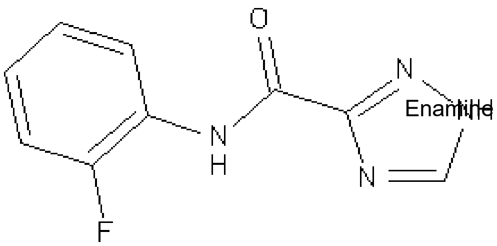

Enamine BB Make on Demand:BBV-42937000

Mwt: 206.18
xLogP: 0.50
Charge: 0
RotBond: 2
Protomers: 3
Contact: 2
ES: -23.33 VdW: -10.36
Desolv: p=0.96, ap=-0.73

179
82342354
-33.45

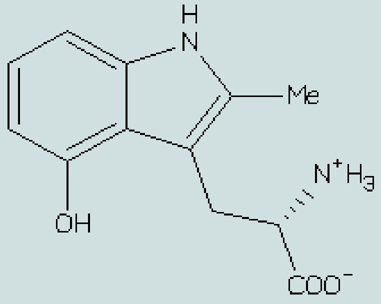

Not for sale

Mwt: 234.255
xLogP: -1.13
Charge: 0
RotBond: 3
Protomers: 2
Contact: 3
ES: -30.19 VdW: -12.18
Desolv: p=8.59, ap=0.33

180
19903722
-33.43

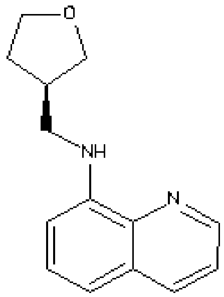

UORSY BB Make-on-demand:BBV-123469
Enamine BB Make on Demand:BBV-123469

Mwt: 228.295
xLogP: 2.24
Charge: 0
RotBond: 3
Protomers: 2
Contact: 2
ES: -29.12 VdW: -15.31
Desolv: p=12.31, ap=-1.31

181
76644971
-33.43

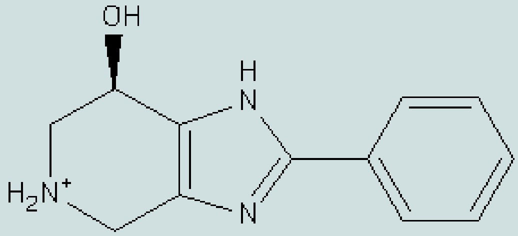

Not for sale

Mwt: 216.264
xLogP: 1.13
Charge: 1
RotBond: 1
Protomers: 5
Contact: 2
ES: -27.65 VdW: -13.29
Desolv: p=8.14, ap=-0.63

182
76647243
-33.42

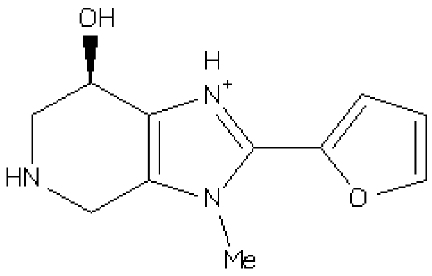

Not for sale

Mwt: 220.252
xLogP: 0.34
Charge: 1
RotBond: 1
Protomers: 3
Contact: 3
ES: -26.85 VdW: -14.01
Desolv: p=8.00, ap=-0.55

183

Enamine BB Make on Demand:BBV-25194362

Mwt: 244.298

36780211
-33.41

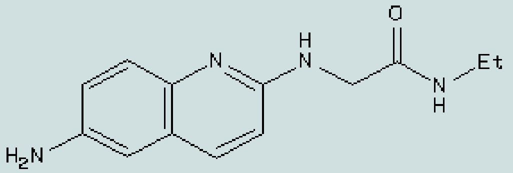

UORSY BB Make-on-demand:BBV-25194362

xLogP: 1.18
Charge: 0
RotBond: 4
Protomers: 2
Contact: 2
ES: -29.57 VdW: -12.75
Desolv: p=9.39, ap=-0.48

184
37756948
-33.41

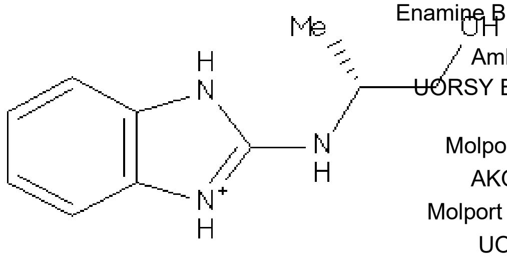

Mcule:MCULE-4196168355
Enamine Building Blocks:EN300-72055
Ambinter:Amb18999259
UORSY BB Make-on-demand:BBV-36630255
Molport:MolPort-014-469-099
AKOS:AKOS008143774
Molport BB:MolPort-014-469-099
UORSY:PB608484478
...

Mwt: 192.242
xLogP: 1.31
Charge: 1
RotBond: 3
Protomers: 1
Contact: 2
ES: -26.47 VdW: -14.77
Desolv: p=8.57, ap=-0.75

185
51921058
-33.41

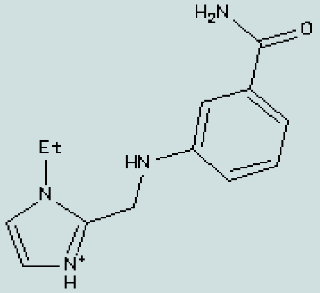

UORSY BB Make-on-demand:BBV-34201388
Enamine BB Make on Demand:BBV-34201388

Mwt: 244.298
xLogP: 0.53
Charge: 0
RotBond: 5
Protomers: 2
Contact: 3
ES: -26.01 VdW: -17.56
Desolv: p=10.35, ap=-0.18

186
82343477
-33.41

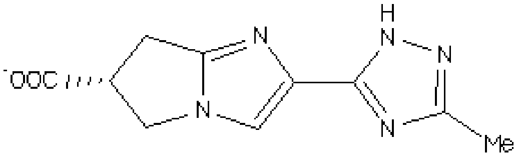

Not for sale

Mwt: 232.223
xLogP: -0.71
Charge: -1
RotBond: 2
Protomers: 4
Contact: 3
ES: -28.13 VdW: -10.69
Desolv: p=6.45, ap=-1.03

187
188311
-33.4

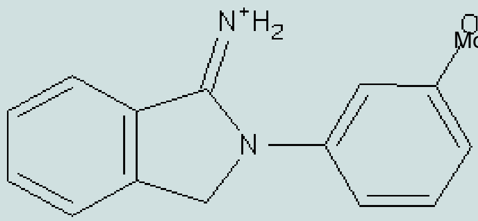

eMolecules:6177132
Mcule Make-on-demand:MCULE-3929478455
Mcule:MCULE-3929478455

Mwt: 225.271
xLogP: 1.72
Charge: 1
RotBond: 1
Protomers: 1
Contact: 2
ES: -28.35 VdW: -13.47
Desolv: p=9.71, ap=-1.28

188
5808044

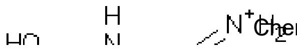

Vitas-M:STK246890
Chemical Block:A1934/0081388

Mwt: 220.208
xLogP: -0.83

-33.4

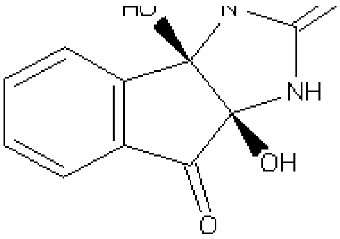

Princeton BioMolecular Research:OSSK_784158

Charge: 1
RotBond: 0
Protomers: 1
Contact: 3
ES: -33.84 VdW: -11.99
Desolv: p=10.52, ap=1.91

189
82741378
-33.4

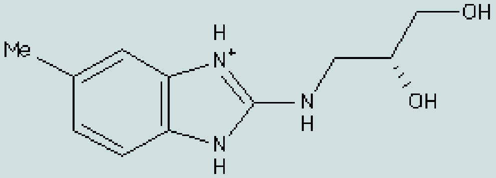

Not for sale

Mwt: 221.26
xLogP: 0.76
Charge: 0
RotBond: 4
Protomers: 3
Contact: 2
ES: -30.00 VdW: -10.83
Desolv: p=8.02, ap=-0.59

PDB

190
8700522
-33.37

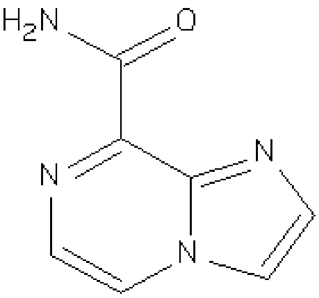

CiVentiChem:CV-4109
Ryan Scientific BB:048-07741

Mwt: 162.152
xLogP: -0.54
Charge: 0
RotBond: 1
Protomers: 2
Contact: 2
ES: -34.97 VdW: -9.20
Desolv: p=11.19, ap=-0.39

191
4250891
-33.36

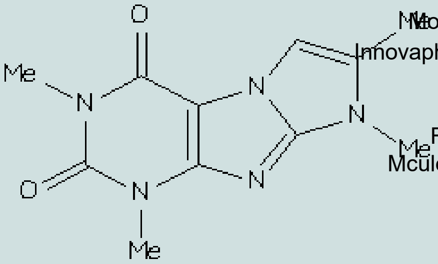

Mcule:MCULE-4465022929
Molport:MolPort-003-133-090
Innovapharm BB Make on Demand:BBV-00069744
Princeton BioMolecular Research:OSSL_719621
Mcule Make-on-demand:MCULE-4465022929

Mwt: 247.258
xLogP: 0.65
Charge: 0
RotBond: 0
Protomers: 2
Contact: 1
ES: -26.78 VdW: -14.77
Desolv: p=10.50, ap=-2.31

192
42571916
-33.36

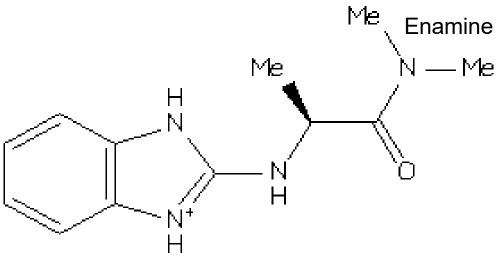

UORSY BB Make-on-demand:BBV-36663267
Enamine BB Make on Demand:BBV-36663267

Mwt: 233.295
xLogP: 0.85
Charge: 1
RotBond: 3
Protomers: 1
Contact: 2
ES: -23.59 VdW: -16.30
Desolv: p=7.83, ap=-1.30

193
82754283
-33.36

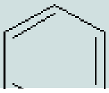

Mwt: 235.29
xLogP: 3.22
Charge: 0

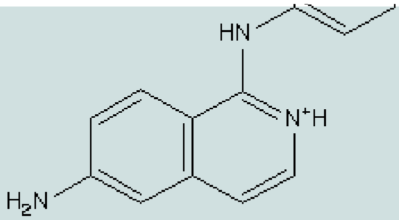

Not for sale

RotBond: 2
Protomers: 2
Contact: 2
ES: -24.29 VdW: -14.99
Desolv: p=7.25, ap=-1.33

194
22153931
-33.35

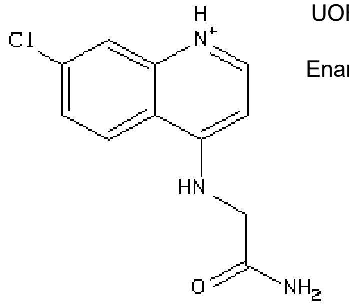

UORSY BB Make-on-demand:BBR-063715
Enamine BB Make on Demand:BBR-063715

Mwt: 236.682
xLogP: 1.42
Charge: 1
RotBond: 3
Protomers: 2
Contact: 2
ES: -28.67 VdW: -13.11
Desolv: p=8.63, ap=-0.21

195
82303307
-33.35

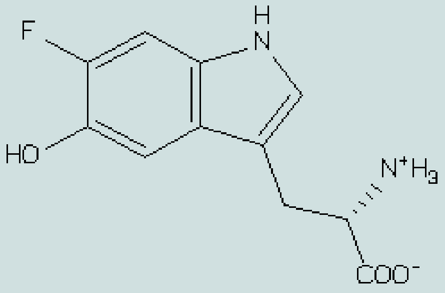

Not for sale

Mwt: 238.218
xLogP: -1.23
Charge: 0
RotBond: 3
Protomers: 3
Contact: 3
ES: -30.22 VdW: -8.81
Desolv: p=5.67, ap=0.01

196
82304771
-33.35

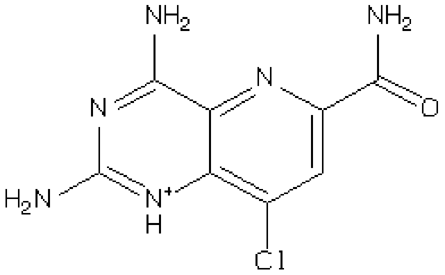

Not for sale

Mwt: 239.646
xLogP: -0.07
Charge: 1
RotBond: 1
Protomers: 1
Contact: 1
ES: -30.39 VdW: -13.35
Desolv: p=10.15, ap=0.25

197
15906891
-33.34

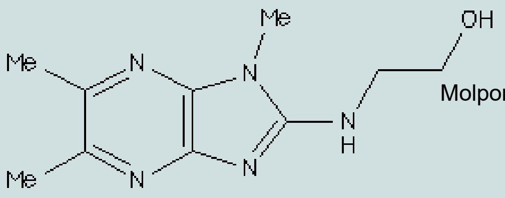

Molport:MolPort-002-514-308

Mwt: 221.264
xLogP: 0.08
Charge: 0
RotBond: 3
Protomers: 2
Contact: 2
ES: -27.84 VdW: -14.03
Desolv: p=9.59, ap=-1.06

198
19951144
-33.34

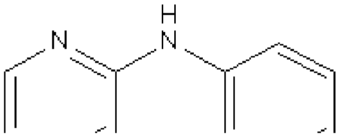

Not for sale

Mwt: 215.256
xLogP: 2.84
Charge: 0
RotBond: 2
Protomers: 2
Contact: 2

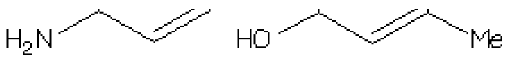

ES: -27.07 VdW: -11.93
Desolv: p=6.68, ap=-1.02

199
40661733
-33.34

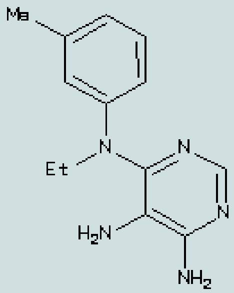

Innovapharm Make-on-Demand:VT-00310738

Mwt: 243.314
xLogP: 1.97
Charge: 0
RotBond: 3
Protomers: 2
Contact: 2
ES: -28.80 VdW: -12.92
Desolv: p=8.92, ap=-0.54

200
21948393
-33.33

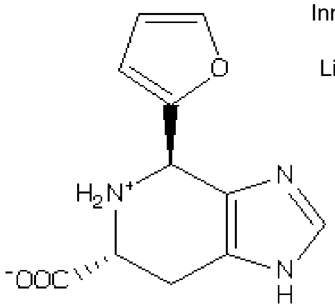

Innovapharm BB Make on Demand:BBV-00051621
Life Chemicals Building Blocks:F2135-0429
Ambinter:Amb3633857

Mwt: 233.227
xLogP: -1.18
Charge: 0
RotBond: 2
Protomers: 1
Contact: 2
ES: -28.13 VdW: -11.02
Desolv: p=6.48, ap=-0.65

Table S2. Drugs tested in initial screen for anti-*Salmonella* activity

	Molport ID	Name of compound
1	MolPort-001-572-916	N-(3,4-dimethoxyphenyl)-1H-1,2,4-triazole-3carboxamide
2	MolPort-001-995-052	N-(4-acetylphenyl)-1H-1,2,4-triazole-3-carboxamide
3	MolPort-030-064-032	3-methyl-5-{1-[(4-methyl-1H-imidazol-2yl)methyl]pyrrolidin-2-yl}-1H-1,2,4-triazole
4	MolPort-001-495-440	ethyl 7-amino-3-carbamoylpyrazolo[1,5-a]pyrimidine-6carboxylate
5	MolPort-008-431-209	N-ethyl-2-oxo-2,3-dihydro-1H-1,3-benzodiazole-5sulfonamide
6	MolPort-002-639-017	1,6,7,8-tetramethyl-1H,2H,3H,4H,8H-imidazo[1,2g]purine-2,4-dione
7	MolPort-005-594-067	3-[({imidazo[1,2-a]pyridin-2-yl}methyl)sulfanyl]-5-methyl-4H-1,2,4-triazole
8	MolPort-005-857-675	5-phenyl-N-[(2E)-pyrrolidin-2-ylidene]-1,3,4-oxadiazol-2amine
9	MolPort-008-928-220	N-(butan-2-yl)-2-{imidazo[1,2-a]pyridin-2-yl}acetamide
10	MolPort-011-526-629	3-(1-methyl-1H-1,3-benzodiazol-2-yl)phenol
11	MolPort-027-670-022	1-{[(4-aminoquinazolin-2-yl)amino]methyl}cyclobutan-1-ol
12	MolPort-008-319-228	4-{3H-imidazo[4,5-b]pyridin-2-yl}aniline
13	MolPort-002-860-974	5-oxo-3-phenyl-2,5-dihydro-1H-pyrazole-1carboximidamide
14	MolPort-002-876-167	5-[5-(dimethylamino)-1,3,4-oxadiazol-2-yl]pyrimidine-2,4diamine
15	MolPort-007-995-370	2-(5-amino-3-methyl-1H-pyrazol-1-yl)-3,4,5,6,7,8hexahydroquinazolin-4-one
16	MolPort-003-133-090	1,3,7,8-tetramethyl-1H,2H,3H,4H,8H-imidazo[1,2g]purine-2,4-dione
17	MolPort-019-848-329	4-[(1-methyl-1H-pyrazol-4-yl)amino]-5,6,7,8tetrahydropteridin-6-one
18	MolPort-019-848-285	4-[(1-methyl-1H-pyrazol-3-yl)amino]-5,6,7,8tetrahydropteridin-6-one
19	MolPort-002-514-308	2-({trimethyl-1H-imidazo[4,5-b]pyrazin-2-yl}amino)ethan1-ol
20	MolPort-019-786-819	6-methyl-5-(2-phenylethyl)pyrimidine-2,4-diamine
21	MolPort-014-469-099	2-[(1H-1,3-benzodiazol-2-yl)amino]propan-1-ol
22	MolPort-002-257-396	N,N-dimethyl-2-oxo-2,3-dihydro-1H-1,3-benzodiazole-5sulfonamide
23	MolPort-002-335-677	2-[(1H-1,2,4-triazol-5-ylsulfanyl)methyl]-1H-1,3benzodiazole
24	MolPort-005-310-215	10-(2-hydroxyethyl)-11-methyl-1,8,10-

		triazatricyclo[7.4.0.0^{2},^{7}]trideca-2,4,6,8,11-pentaen-13-one
25	MolPort-002-507-542	(2S)-4-methyl-2-[(9H-purin-6-yl)amino]pentanoic acid

Table S3. Screen of anti-DksA drugs

name	structure	formula	MW	MIC (μg/ml)	IVT (livJ) (μM)
VKT-17-P4-6	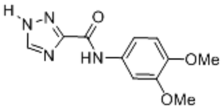	$C_{11}H_{12}N_4O_2$	248.24	32	<1
VKT-17-P4-8	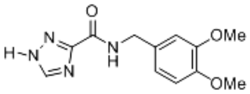	$C_{12}H_{14}N_4O_3$	262.26	>32	>1
VKT-17-P4-9	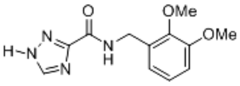	$C_{12}H_{14}N_4O_3$	262.26	32	<0.1
VKT-17-P4-10	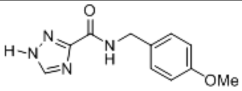	$C_{11}H_{12}N_4O_2$	232.24	8	>1
VKT-17-P4-11	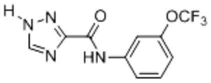	$C_{10}H_7F_3N_4O_2$	272.18	>32	<0.1
VKT-17-P4-12	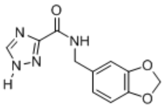	$C_{11}H_{10}N_4O_3$	246.22	8	>1
VKT-17-P4-13	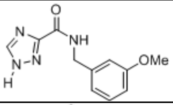	$C_{11}H_{12}N_4O_2$	232.24	8	>1
VKT-17-P4-14	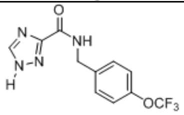	$C_{11}H_9F_3N_4O_2$	286.21	>32	<0.1
VKT-17-P4-15	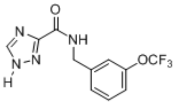	$C_{11}H_9F_3N_4O_2$	286.21	>32	<0.1
VKT-17-P4-16	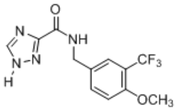	$C_{12}H_{11}F_3N_4O_2$	300.24	>32	<1
VKT-17-P4-17	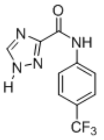	$C_{10}H_9F_3N_4O_2$	256.18	>32	<0.1
VKT-17-P4-18	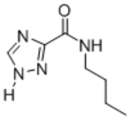	$C_7H_{12}N_4O$	168.20	8	<0.1
VKT-17-P4-19	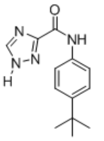	$C_{13}H_{16}N_4O$	244.29	>32	<0.1

VKT-17-P4-21	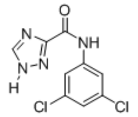	$C_9H_6Cl_2N_4O$	257.08	8	<0.1
VKT-17-P4-22	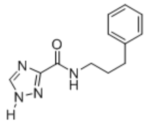	$C_{12}H_{14}N_4O$	230.27	16	<0.1
VKT-17-P4-23	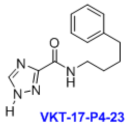 VKT-17-P4-23	$C_{11}H_9F_3N_4O_2$	244.29	16	<0.1
VKT-17-P4-24	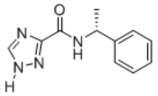	$C_{12}H_9F_3N_4O_2$	216.24	>32	<0.1
VKT-17-P4-25	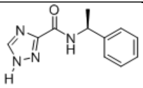	$C_{10}H_9F_3N_4O_2$	216.24	>32	<0.1
VKT-17-P4-27	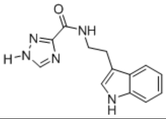	$C_7H_{12}N_4O$	255.28	16	<0.1
VKT-17-P4-28	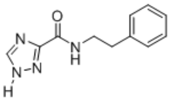	$C_{13}H_{16}N_4O$	216.24	8	<0.1
VKT-17-P4-29	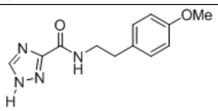	$C_9H_6Cl_2N_4O$	246.27	32	<0.1
VKT-17-P4-32	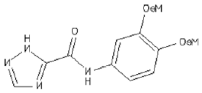	$C_{11}H_9F_3N_4O_2$	246.27	>32	<0.1

Table S4. MIC values

**Strain name**	**VKT-17-P4-21 (μg/ml)**	**VKT-17-P4-23 (μg/ml)**
Serratia odorifera	16,16, 8	32, 16, 16
S. enterica serovar Typhimurium	8, 8, 8	16, 32, 16
Salmonella Newport	4, 8, 8	32, 32, 16
Salmonella Enteritidis	64, 64, 32	>128, >128, >128
Proteus mirabilis	64, 128,128	>128, >128, >128
Proteus hauseri	8, 8, 8	>128, >128, >128
Klebsiella pneumoniae	16,16,16	64, 64, 64
Klebsiella oxytoca	64, 64, 128	32, 32, 32
Escherichia coli	16, 16,8	64, 64, 64
Enterobacter cloacae	64, 64, 128	>128, >128, >128

Table S5. Bacterial strains.

ID	Strain	Source
14028s	*S. enterica* serovar Typhimurium	ATCC
AV07265	S. Typhimurium Δ*dksA::Cm*	Ref 37
AV0201	S. Typhimurium Δ*spiC::km*	Ref 42
AV25001	*Serratia odorifera*	This study
AV25002	*Salmonella* Typhimurium	This study
AV25003	*Salmonella* Newport	This study
AV25004	*Salmonella* Enteritidis	This study
AV25005	*Proteus mirabilis*	This study
AV25006	*Proteus hauseri*	This study
AV25007	*Klebsiella pneumoniae*	This study
AV25008	*Klebsiella oxytoca*	This study
AV25009	*Escherichia coli*	This study
AV25010	*Enterobacter cloacae*	This study
AV25011	*Acinetobacter baumannii*	This study
AV25012	*Acinetobacter radioresistens*	This study
AV10314	*E.coli* W3110	
AV11276	P_{sifA}::luc aph (WT14028s)	This study
BL21(DE3)	F^- *ompT hsd*S_B(r_B^- m_B^-) *gal dcm* (DE3)	Invitrogen
AV25098	BL21(DE3) (pET22b::*dksA*)	This study
AV21188	BL21(DE3) (pET14b::*greA*)	Ref 36
AV21189	BL21(DE3) (pET14b::*greA*)	Ref 36

Table S6. Plasmids used in this study.

Plasmid	Relevant characteristics	Reference
pTIM	bla *rrnB* & *rpoC* term pBluescript	Ref 37
pTIM-*livJ*	pTim + 1.34-kb DNA containing P*livJ* (-240) and *livJ*	Ref 35
pTIM-*rpsM*	pTim + 0.56-kb DNA containing P*rpsM* (-203) and *rpsM*	This study
pTIM-*hisG*	pTim + 0.80-kb DNA containing P*hisG* (-352) and *hisG*	This study
pET22b(+)	*ori* pBR322, C-terminal His·Taq fusion vector, Pn^r	Novagen
pET-*dksA*	pET22b + 0.46-kb DNA containing *dksA*, Pn^r	This study
pET14b	*ori* pBR322, N-terminal His·Taq fusion vector, Pn^r	Novagen
pET-*greA*	pET22b + 0.47-kb DNA containing *dksA*, Pn^r	Ref 36
pET-*greB*	pET22b + 0.47-kb DNA containing *dksA*, Pn^r	Ref 36

Table S7. Oligonucleotides used in this study.

	Primer Sequence (5’ → 3’)
qRT-PCR	
rpsM	F: AGTTGCCAAATTTGTCGTTG
	R: TACGAGCGTTGGTCTTGGTA
	Probe: 6-FAM-TGAAATCAGCATGAGCATCAA -3BHQ-1
livJ	F: CGCAGGGCTGAAAACCCA
	R: CACACGAATGCGCCGCTA
	Probe: 6-FAM-TCAGCGGAAGGCTTACTGGTC-3BHQ-1
hisG	F: CAGGCCGTTTAAGCGATGATTCACGAG
	R: AATACCGAGATCGACCACGCCATCC
	Probe: 6-FAM-ATCGGCATGTTTTCCGCCATCGCAATCAGG-3BHQ-1
**Plasmid**	
pTIM-*hisG*	F: ACTGAATTCTCATACGGGCCGCTTCCAC (EcoRI)*
	R: ACTCTGCAGCAGCCGCCGAAGTCAAGAC (PstI)
	F: CATATGCAAGAAGGGCAAAACCGT (NdeI)
	R: CTCGAGACCCGCCATCTGTTTTTCGCG (XhoI)

*Restriction sites are underlined.

Supplementary Figures:

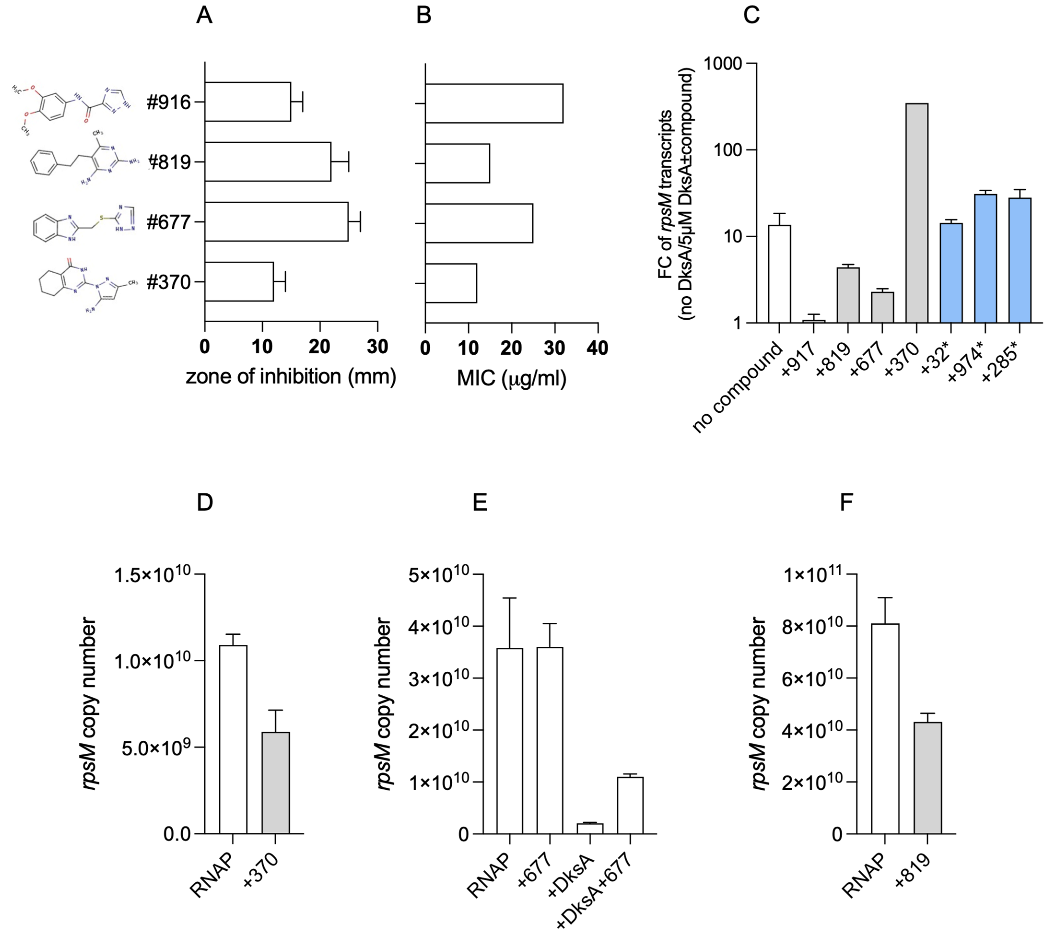

Figure S1. Effects of compounds on *Salmonella* growth and *rpsM* transcription. Antimicrobial effects of the indicated drugs as measured by (A) zone of inhibition and (B) MIC values. MIC assays were performed using overnight cultures plated on M9 minimal agar. (C) To identify potential inhibitors of DksA-dependent transcription, candidate compounds were screened for their effects on *rpsM* expression using qRT-PCR. The transcriptional activity of *rpsM* was assessed *in vitro* with 10 μM compound in the presence or absence of 5 μM DksA. The white bar represents fold *rpsM* repression by DksA alone in the absence of any compound. Gray bars indicate significant differences from the control (*917, 819, 677,* and *370*), whereas blue bars represent compounds (32, 974, 285) that showed no significant differences. Among the tested compounds, 370 and 819 directly inhibited *rpsM* transcription (D and F). In contrast, compound 677 did not repress *rpsM* expression alone but exhibited minor derepression in the presence of DksA (E). RNAP in D, E, and F was tested in the absence of DksA and compound as a control. Data are the mean ± SD from 2-3 independent experiments.

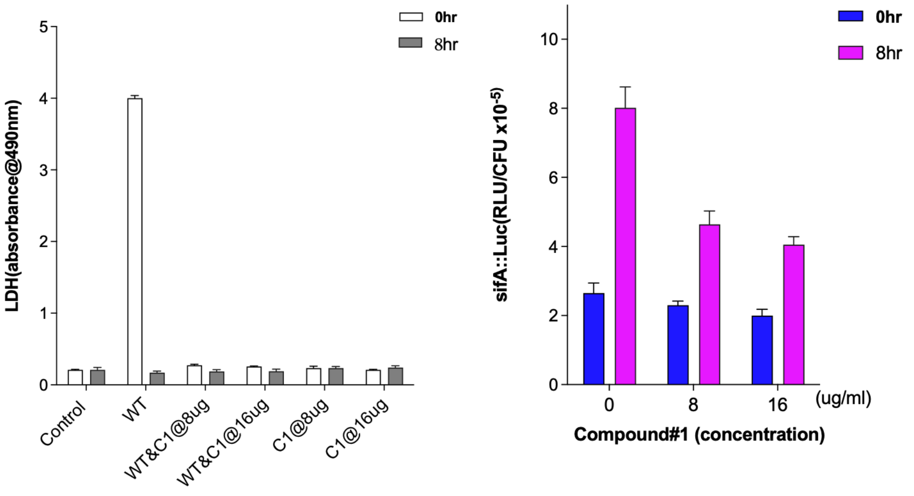

Figure S2. Effects of compound #916 on cytotoxicity and SPI-2 expression. (A) Lactate dehydrogenase, as determined using a Roche cytotoxicity detection kit, from J774 infected with *sifA:luc*$^{+}$ *Salmonella* at MOI of 20. The specimens were treated with different contrations of compound. Data are frow two individual experiments (n=8). (B) Expression of the P_{sifA}::luc construct in J774 cells infected with *Salmonella* at MOI of 2. At the times tested, the addition of compound did not have an effect on the intracellular growth of *Salmonella* (n=8).

Figure S3: Chemical synthesis of analogs

Chemicals and Synthesis:

Anhydrous DMF (dimethylformamide), HPLC grade water, acetonitrile (ACN), dimethyl sulfoxide (DMSO), ethyl acetate (EtOAc), hexanes, methanol (MeOH), methylene chloride (DCM), anhydrous sodium sulfate (Na_2SO_4), ammonium acetate (NH_4OAC) and formic acid were purchased from Fisher Scientific (Pittsburgh, PA). 1*H*-1,2,4-Triazole-3-carboxylic acid, DMSO-d_6, 3,4-dimethoxyaniline, 1-hydroxybenzotriazole monohydrate (HOBt), EDC (*N*-(3-dimethylaminopropyl)-*N′*-ethylcarbodiimide), *N*-methylmorpholine (NMM), (3,4-dimethoxybenzyl)amine, (2,3-dimethoxybenzyl)amine, (4-methoxyphenyl)methanamine, 3-(trifluoromethoxy)aniline, piperonylamine, 4-(trifluoromethoxy)benzylamine, 3-methoxy-benzylamine, 3-(trifluoromethoxy)benzylamine, 4-methoxy-3-(trifluoromethyl)benzylamine, 4-(trifluoromethyl)aniline, butan-1-amine, 4-(*tert*-butyl)aniline, 3,5-dichloroaniline, 3-phenylpropan-1-amine, 4-phenylbutan-1-amine, (R)-(+)-α-methyl benzyl amine, (S)-(α)-methyl benzyl amine, 2-(1*H*-indol-3-yl)ethanamine, 2-phenylethanamine, 2-(4-methoxyphenyl) ethanamine, (R)-(+)-4-Methoxy-α-methylbenzylamine were procured from Sigma-Aldrich Chemical Company (St. Louis, MO).

To a stirred reaction of 1*H*-1,2,4-triazole-3-carboxylic acid (1, 226 mg, 2.0 mmol) and amine (2, 2.1 mmol) in dry DMF (10 mL) was added (HOBt; 337 mg, 2.2 mmol) and EDC (422 mg, 2.2 mmol) followed by NMM (0.88 mL, 8.0 mmol) via syringe. The mixture was stirred at room temperature (RT) under nitrogen (N_2) and the solids were gradually dissolved. The contents were stirred at RT (24 h), and then slowly diluted into ice cold water and extracted with DCM (50 mL × 2). The DCM phase was washed with cold water (100 mL × 2). The DCM phase was dried over anhydrous Na_2SO_4, filtered, concentrated under reduced pressure, and then chromatographed on silica gel to get the desired analog.

Chemical synthesis reactions were monitored via silica gel IB2-F thin layer chromatography (TLC) plates from J.T. Baker (Phillipsburg, NJ). Silica Gel 60 Å 40-63 μm was purchased from Sorbent Technologies (Norcross, GA). The ^{1}H (proton) and ^{19}F NMR (Nuclear Magnetic Resonance) spectra were recorded using a 400 MHz Bruker NMR, Avance III 400. Chemical shifts are reported in ppm. An Applied Biosystems Sciex 4000 (Applied Biosystems; Foster City, CA) was equipped with a Shimadzu HPLC (Shimadzu Scientific Instruments, Inc.; Columbia, MD) and Leap auto-sampler (LEAP Technologies; Carrboro, NC) was used to perform the LC/MS-MS. Nitrogen gas was procured from AirGas® (Denver, CO). Reverse phase chromatography was performed using a Zorbax extended C18 (50 x 46 mm, 5-micron column) with a column guard at 40°C at a flow rate of 0.4 mL/min. Solvent A: HPLC water, 10 mM NH_4OAc, 0.1% Formic acid; Solvent B: Methanol:Acetonitrile (1:1). Gradient conditions were used for the compounds and denoted for each compound (below) and compounds monitored via electro-spray ionization positive mode (ESI+) at 450°C, curtain gas set at 10, collisional activated dissociation (CAD, set at 12 with N_2 as the gas), ion source one (GS1) and two (GS2) were set at 30 with an entrance potential set at 10V. Quadruple one (Q1) and Q3 were set to unit resolution with a dwell time of 200 msec. Samples (10 μL) were analysed by LC/MS-MS methods.

VKT-17-P4-6

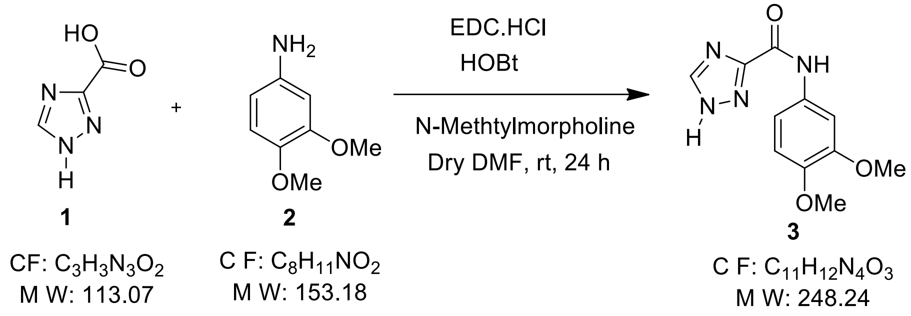

To a reaction of 1*H*-1,2,4-triazole-3-carboxylic acid (**1**, 226 mg, 2.0 mmol) and 3,4-dimethoxyaniline (**2**, 322 mg, 2.1 mmol) in dry DMF (10 mL) was added 1-hydroxybenzotriazole monohydrate (337 mg, 2.2 mmol) and EDC (N-(3-dimethylaminopropyl)-N′-ethylcarbodiimide) (422 mg, 2.2 mmol) followed by N-methylmorpholine (0.88 mL, 8.0 mmol via syringe). The mixture was stirred at room temperature under nitrogen and the solids were gradually dissolved. The contents were stirred at room temperature for 24.0 h, and then slowly diluted into ice cold water and extracted with DCM (50 mL × 2). The DCM phase was washed with cold water (100 mL × 2). The DCM phase was dried over anhydrous Na_2SO_4, filtered and concentrated under reduced pressure and then chromatographed on silica gel using EtOAc as eluent to get the desired amide. ESI(+) MH^+ = 249 → 153 *m/z*; t_R = 3.0 min.

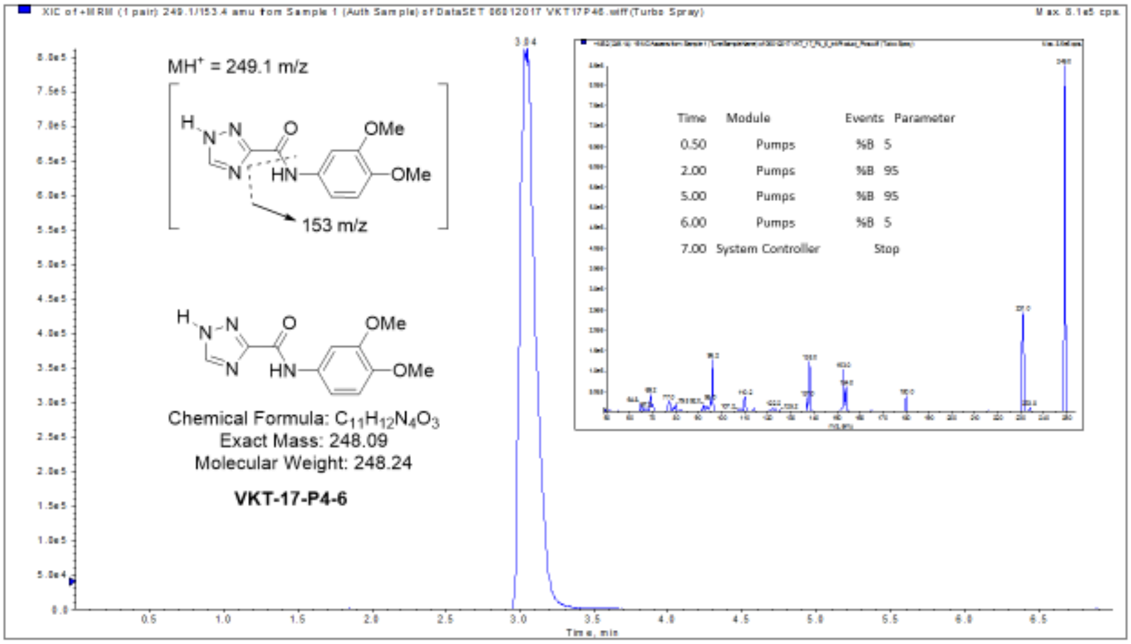

VKT-17-P4-8

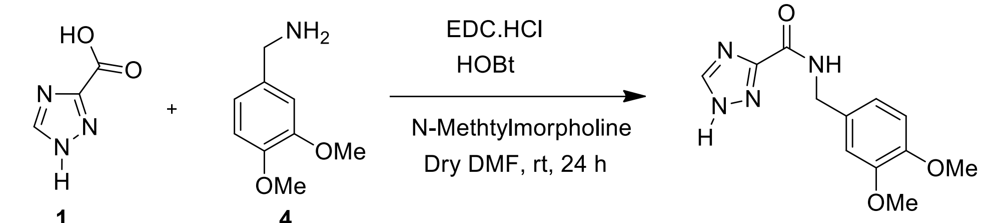

1
CF: $C_3H_3N_3O_2$
M W: 113.07

4
C F: $C_9H_{13}NO_2$
M W: 167.21

5
C F: $C_{12}H_{14}N_4O_3$
M W: 262.26

To a reaction of 1*H*-1,2,4-triazole-3-carboxylic acid (**1,** 226 mg, 2.0 mmol) and (3,4-dimethoxybenzyl)amine (**4**, 0.32 mL, 2.1 mmol) in dry DMF (10 mL) was added 1-hydroxybenzotriazole monohydrate (337 mg, 2.2 mmol) and EDC (N-(3-Dimethylaminopropyl)-N′-ethylcarbodiimide) (422 mg, 2.2 mmol) followed by N-methylmorpholine (0.88 mL, 8.0 mmol via syringe). The mixture was stirred at room temperature under nitrogen and the solids were gradually dissolved. The contents were stirred at room temperature for 24 h, and then slowly diluted into iced water and extracted with DCM (50 mL × 2). The DCM phase was washed with ice cold water (2 × 100 mL). The DCM phase was dried over anhydrous Na_2SO_4, filtered and concentrated under reduced pressure and chromatographed on silica gel using EtOAc and Hexanes (70:30) as eluents to get the desired amide **5** (**VKT-17-P4-8**, 40.7 mg, 8% yield) as a white solid. ^{1}H-NMR (400 MHz, DMSO-*d*$_6$): δ14.74–14.50 (m, 1H), 9.06 (br s, 1H), 8.48–8.46 (m, 1H), 6.97 (m, 1H), 6.90–6.88 (m, 1H), 6.85–6.83 (m, 1H), 4.37 (d, *J* = 6.4 Hz, 2H), 3.73 (d, *J* = 3.6 Hz, 6H) ppm. ESI(+) MH^+ = 263→ 152 *m/z*; t_R = 3.0 min.

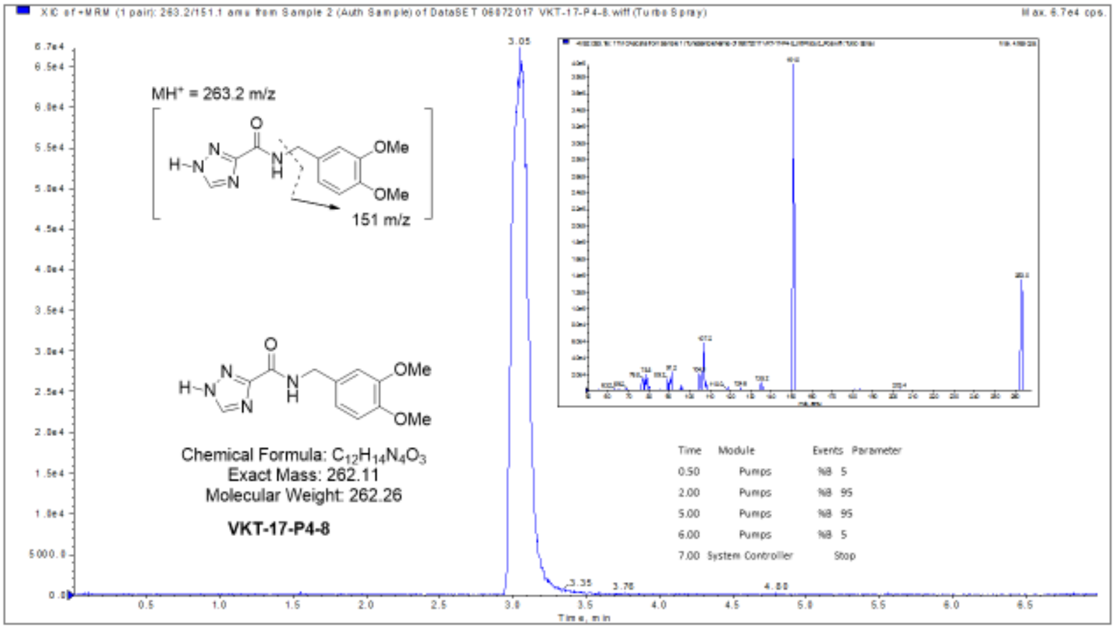

VKT-17-P4-9

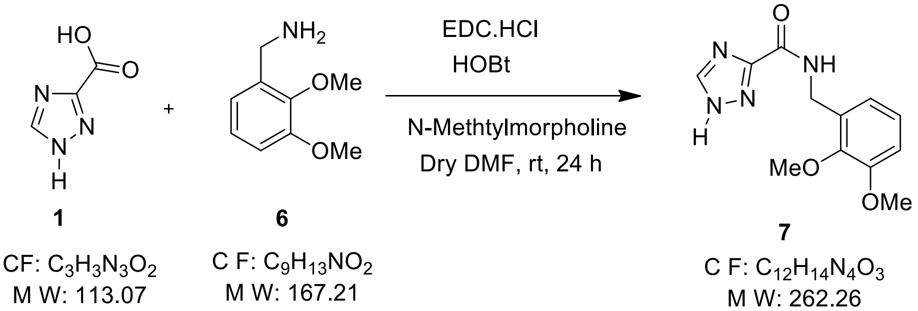

1 CF: $C_3H_3N_3O_2$ M W: 113.07

6 C F: $C_9H_{13}NO_2$ M W: 167.21

7 C F: $C_{12}H_{14}N_4O_3$ M W: 262.26

To a reaction of 1*H*-1,2,4-triazole-3-carboxylic acid (**1,** 226 mg, 2.0 mmol) and (2,3-dimethoxybenzyl)amine (**6**, 0.31 mL, 2.1 mmol) in dry DMF (10 mL) was added 1-hydroxybenzotriazole monohydrate (337 mg, 2.2 mmol) and EDC (N-(3-Dimethylaminopropyl)-N′-ethylcarbodiimide) (422 mg, 2.2 mmol) followed by N-methylmorpholine (0.88 mL, 8.0 mmol via syringe). The mixture was stirred at room temperature under nitrogen and the solids were gradually dissolved. The contents were stirred at room temperature for 24 h, and then slowly diluted into ice cold water and extracted with DCM (2 × 50 mL). The DCM phase was washed with ice cold water (2 × 100 mL). The DCM phase was dried over anhydrous Na_2SO_4, filtered and concentrated under reduced pressure and chromatographed on silica gel using EtOAc and Hexanes (9:1) as eluents to get the desired amide **7** (**VKT-17-P4-9** , 104.9 mg, 20% yield) as a white solid. ^{1}H-NMR (400 MHz, DMSO-d_6): δ14.67 (br s, 1H), 8.94 (br s, 1H), 8.49 (br s, 1H), 7.03–6.99 (m, 1H), 6.99–6.94 (m, 1H), 6.85–6.82 (m, 1H), 4.48 (d, *J* = 6.4 Hz, 2H), 3.80 (s, 3H), 3.78 (s, 3H) ppm. ESI(+) MH^+ = 263→ 151 *m/z*; t_R = 3.2 min.

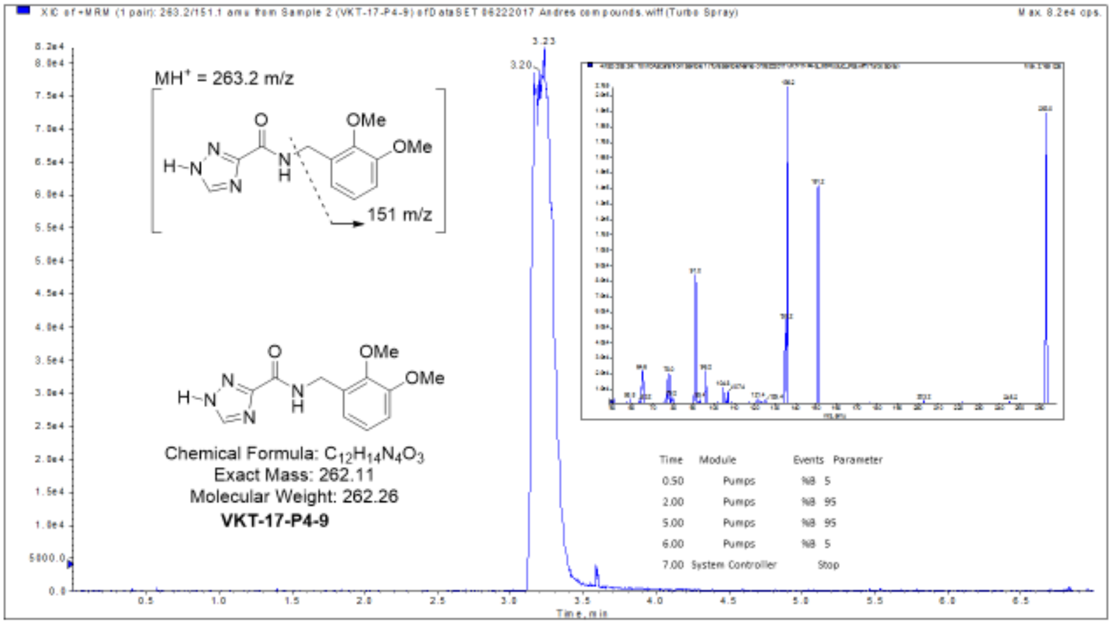

VKT-17-P4-10

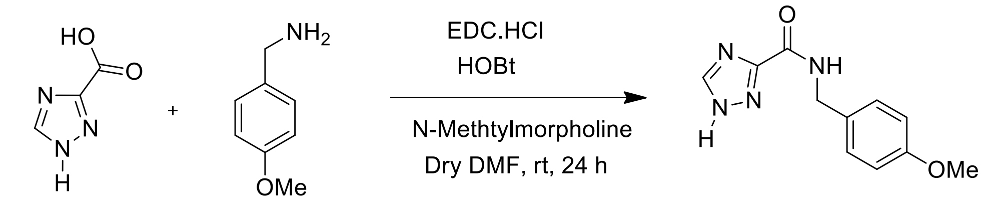

1 CF: $C_3H_3N_3O_2$ M W: 113.07

8 C F: $C_8H_{11}NO$ M W: 137.18

9 C F: $C_{11}H_{12}N_4O_2$ M W: 232.24

To a reaction of 1*H*-1,2,4-triazole-3-carboxylic acid (**1,** 226 mg, 2.0 mmol) and (4-methoxyphenyl)methanamine (**8**, 0.27 mL, 2.1 mmol) in dry DMF (10 mL) was added 1-hydroxybenzotriazole monohydrate (337 mg, 2.2 mmol) and EDC (N-(3-Dimethylaminopropyl)-N′-ethylcarbodiimide) (422 mg, 2.2 mmol) followed by N-methylmorpholine (0.88 mL, 8.0 mmol via syringe). The mixture was stirred at room temperature under nitrogen and the solids were gradually dissolved. The contents were stirred at room temperature for 24.0 h, and then slowly diluted into iced water and extracted with DCM (2 × 50 mL). The DCM phase was washed with ice cold water (2 × 100 mL). The DCM phase was dried over anhydrous Na_2SO_4, filtered and concentrated under reduced pressure and chromatographed on silica gel using MeOH/EtOAc/DCM (6:50:50, v/v/v) as eluents to get the desired amide **9** (**VKT-17-P4-10**, 137 mg, 29% yield) as a white solid. ^{1}H-NMR (400 MHz, DMSO-d_6): δ14.70–14.61 (m, 1H), 9.07 (br s, 1H), 8.45 (br s, 1H), 7.24 (d, *J* = 8.4 Hz, 2H), 6.87 (d, *J* = 8.8 Hz, 2H), 4.36 (d, *J* = 6.4 Hz, 2H), 3.72 (s, 3H) ppm. ESI(+) MH^+ = 233→ 121 *m/z*; t_R = 3.1 min.

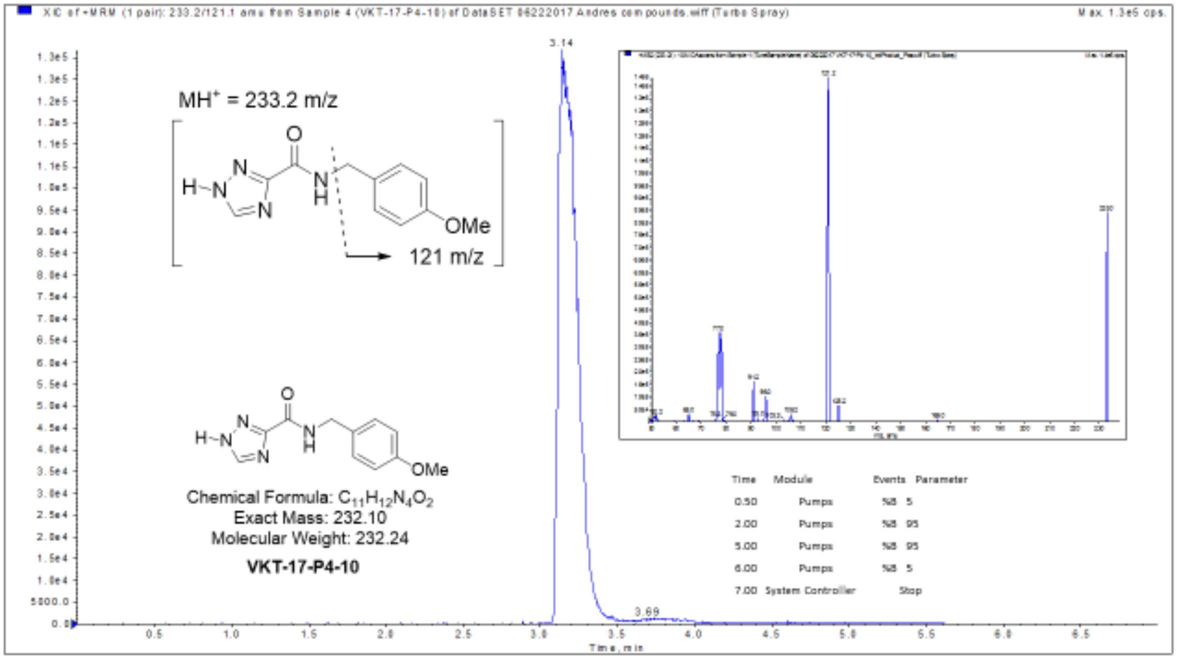

VKT-17-P4-11

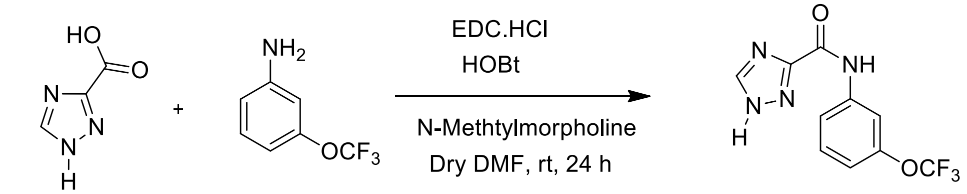

1 CF: $C_3H_3N_3O_2$ M W: 113.07

10 C F: $C_7H_6F_3NO$ M W: 177.12

11 C F: $C_{10}H_7F_3N_4O_2$ M W: 272.18

To a reaction of 1*H*-1,2,4-triazole-3-carboxylic acid (**1,** 226 mg, 2.0 mmol) and 3-(trifluoromethoxy)aniline (**10**, 0.28 mL, 2.1 mmol) in dry DMF (10 mL) was added 1-hydroxybenzotriazole monohydrate (337 mg, 2.2 mmol) and EDC (N-(3-Dimethylaminopropyl)-N′-ethylcarbodiimide) (422 mg, 2.2 mmol) followed by N-methylmorpholine (0.88 mL, 8.0 mmol via syringe). The mixture was stirred at room temperature under nitrogen and the solids were gradually dissolved. The contents were stirred at room temperature for 24.0 h, and then slowly diluted into iced water and extracted with DCM (2 × 50 mL). The DCM phase was washed with ice cold water (2 × 100 mL). The DCM phase was dried over anhydrous Na_2SO_4, concentrated under reduced pressure and chromatographed on silica gel using EtOAc and Hexanes (50:50) as eluents to get the desired amide **11** (**VKT-17-P4-11**, 123.8 mg, 23 % yield) as a white solid. ^{1}H-NMR (400 MHz, DMSO-d_6): δ14.89–14.74 (m, 1H), 10.82 (br s, 1H), 8.67 (br s, 1H), 8.00 (br s, 1H), 7.88 (d, J = 8 Hz, 1H), 7.49 (t, J = 8.2 Hz, 1H), 7.11 (d, J = 8 Hz, 1H); ^{19}F-NMR (376 MHz, DMSO-d_6): δ –56.69 ppm. ESI(+) MH$^+$ = 273→ 96 m/z; t_R = 3.5 min.

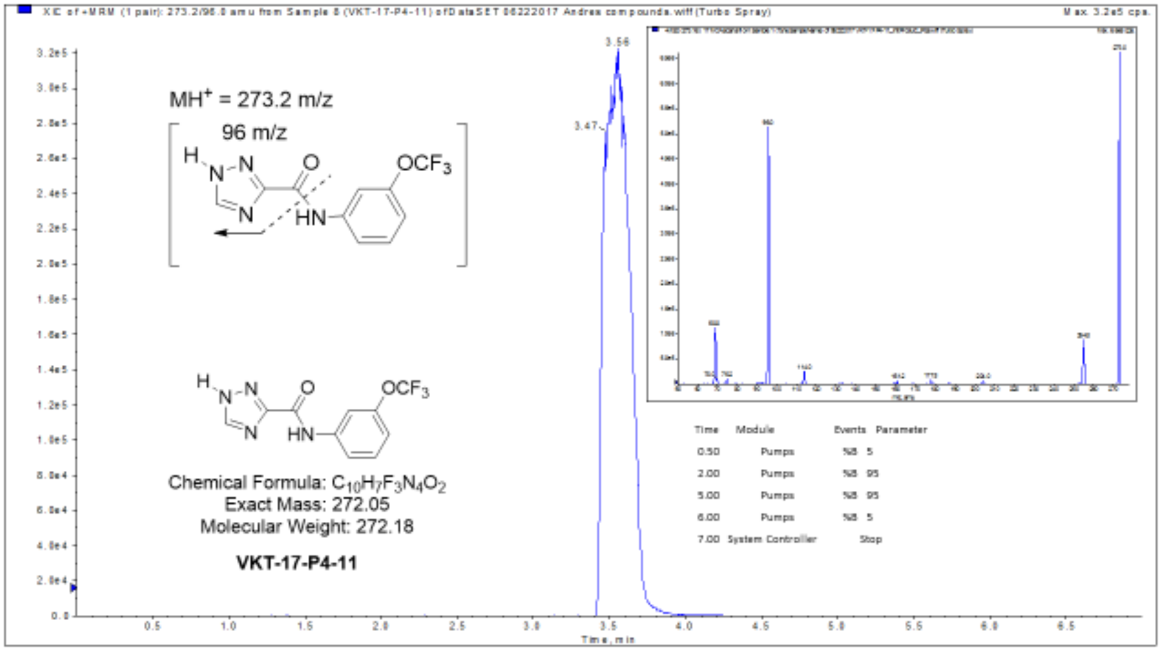

VKT-17-P4-12

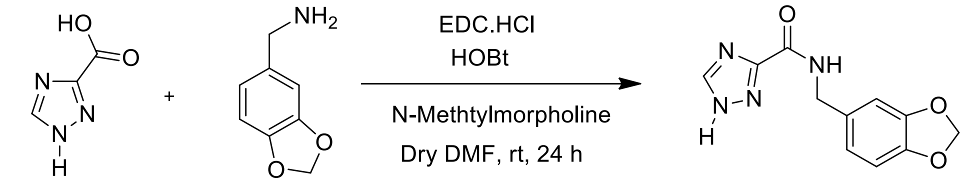

**1**	**12**	**13**
CF: $C_3H_3N_3O_2$	C F: $C_8H_9NO_2$	C F: $C_{11}H_{10}N_4O_3$
M W: 113.07	M W: 151.16	M W: 246.22

To a reaction of 1*H*-1,2,4-triazole-3-carboxylic acid (**1,** 226 mg, 2.0 mmol) and Piperonylamine (**12**, 0.26 mL, 2.1 mmol) in dry DMF (10 mL) was added 1-hydroxybenzotriazole monohydrate (337 mg, 2.2 mmol) and EDC (N-(3-Dimethylaminopropyl)-N′-ethylcarbodiimide) (422 mg, 2.2 mmol) followed by N-methylmorpholine (0.88 mL, 8.0 mmol via syringe). The mixture was stirred at room temperature under nitrogen and the solids were gradually dissolved. The contents were stirred at room temperature for 24.0 h, and then slowly diluted into iced water and extracted with DCM (2 × 50 mL). The DCM phase was washed with ice cold water (2 × 100 mL). The DCM phase was dried over anhydrous Na_2SO_4, filtered and concentrated under reduced pressure and chromatographed on silica gel using EtOAc as eluents to get the desired amide **13** (**VKT-17-P4-12**, 95 mg, 19 % yield) as a white solid. ^{1}H-NMR (400 MHz, DMSO-d_6): δ15.58–13.83 (m, 1H), 9.10 (br s, 1H), 8.47 (br s, 1H), 6.90–6.78 (m, 3H), 5.97 (s, 2H), 4.36 (d, *J* = 6.4 Hz, 2H) ppm. ESI(+) MH^+ = 247→ 135 *m/z*; t_R = 3.3 min.

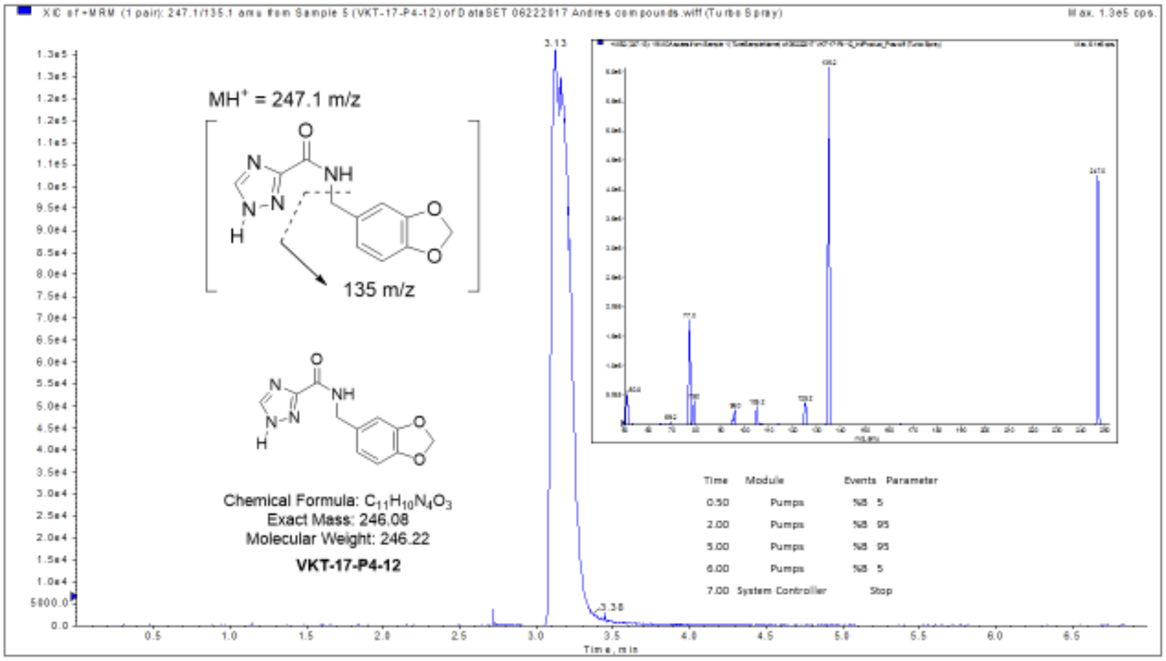

VKT-17-P4-13

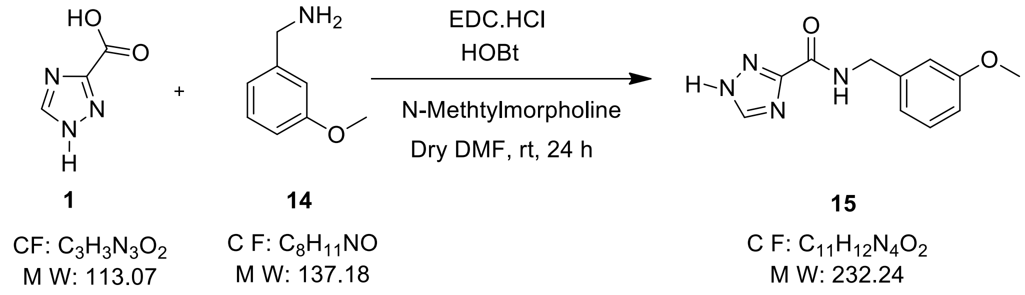

To a reaction of 1*H*-1,2,4-triazole-3-carboxylic acid (**1,** 226 mg, 2.0 mmol) and 3-methoxybenzylamine (**14**, 0.28 mL, 2.2 mmol) in dry DMF (10 mL) was added 1-hydroxybenzotriazole monohydrate (337 mg, 2.2 mmol) and EDC (N-(3-Dimethylaminopropyl)-N′-ethylcarbodiimide) (422 mg, 2.2 mmol) followed by N-methylmorpholine (0.88 mL, 8.0 mmol via syringe). The mixture was stirred at room temperature under nitrogen and the solids were gradually dissolved. The contents were stirred at room temperature for 24.0 h, and then slowly diluted into iced water and extracted with DCM (2 × 50 mL). The DCM phase was washed with ice cold water (2 × 100 mL). The DCM phase was dried over anhydrous Na_2SO_4, concentrated under reduced pressure and chromatographed on silica gel using MeOH and DCM (2:98) as eluents to get the desired amide **15** (**VKT-17-P4-13,** 170.9 mg, 37 % yield) as a white solid compound. ^{1}H-NMR (400 MHz, DMSO-d_6): δ14.76–14.65 (m, 1H), 9.15 (br s, 1H), 8.48 (br s, 1H), 7.23 (t, *J* = 8 Hz, 1H), 6.90–6.88 (m, 2H), 6.82–6.80 (m, 1H), 4.43 (d, *J* = 6 Hz, 2H), 3.73 (s, 3H) ppm. ESI(+) MH$^+$ = 233→ 121 *m/z*; t_R = 3.2 min.

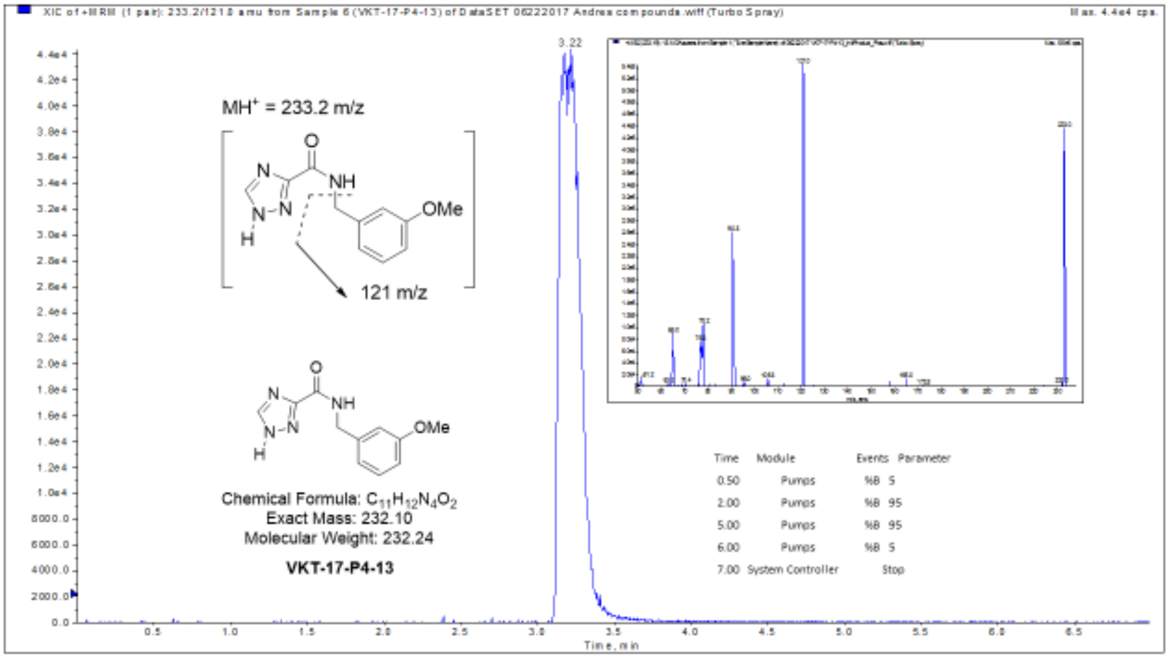

VKT-17-P4-14

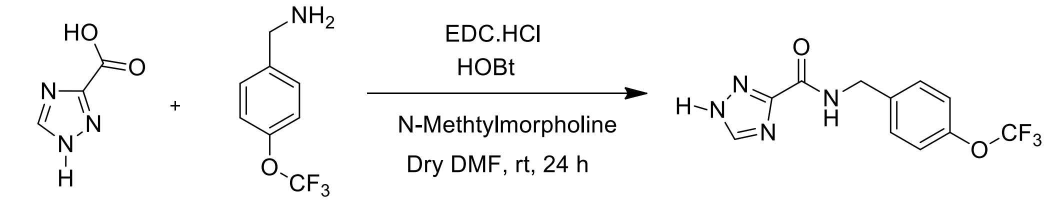

1	16	17
CF: $C_3H_3N_3O_2$	C F: $C_8H_8F_3NO$	C F: $C_{11}H_{12}N_4O_2$
M W: 113.07	M W: 191.15	M W: 232.24

To a reaction of 1*H*-1,2,4-triazole-3-carboxylic acid (**1,** 113 mg, 1.19 mmol) and 4-(trifluoromethoxy)benzylamine (**16**, 0.20 mL, 1.31 mmol) in dry DMF (10 mL) was added 1-hydroxybenzotriazole monohydrate (200 mg, 1.31 mmol) and EDC (N-(3-Dimethylaminopropyl)-N′-ethylcarbodiimide) (251 mg, 1.31 mmol) followed by N-methylmorpholine (0.52 mL, 4.76 mmol via syringe). The mixture was stirred at room temperature under nitrogen and the solids were gradually dissolved. The contents were stirred at room temperature for 24 h, and then slowly diluted into iced water and extracted with DCM (2 × 50 mL). The DCM phase was washed with ice cold water (2 × 100 mL). The DCM phase was dried over anhydrous Na_2SO_4, concentrated under reduced pressure and chromatographed on silica gel using MeOH and DCM (8:92) as eluents to get the desired amide **17** (**VKT-17-P4-14**, 305.3 mg, 90 % yield) as a white solid compound. ^{1}H-NMR (400 MHz, DMSO-d_6): δ14.80–14.01 (br s, 1H), 9.25 (br s, 1H), 8.50 (br s, 1H), 7.44 (d, *J* = 8.4 Hz, 2H), 7.32 (d, *J* = 8 Hz, 2H), 4.47 (d, *J* = 6 Hz, 2H); ^{19}F-NMR (376 MHz, DMSO-d_6): δ –56.89 ppm. ESI(+) MH^+ = 287→ 175 *m/z*; t_R = 3.5 min.

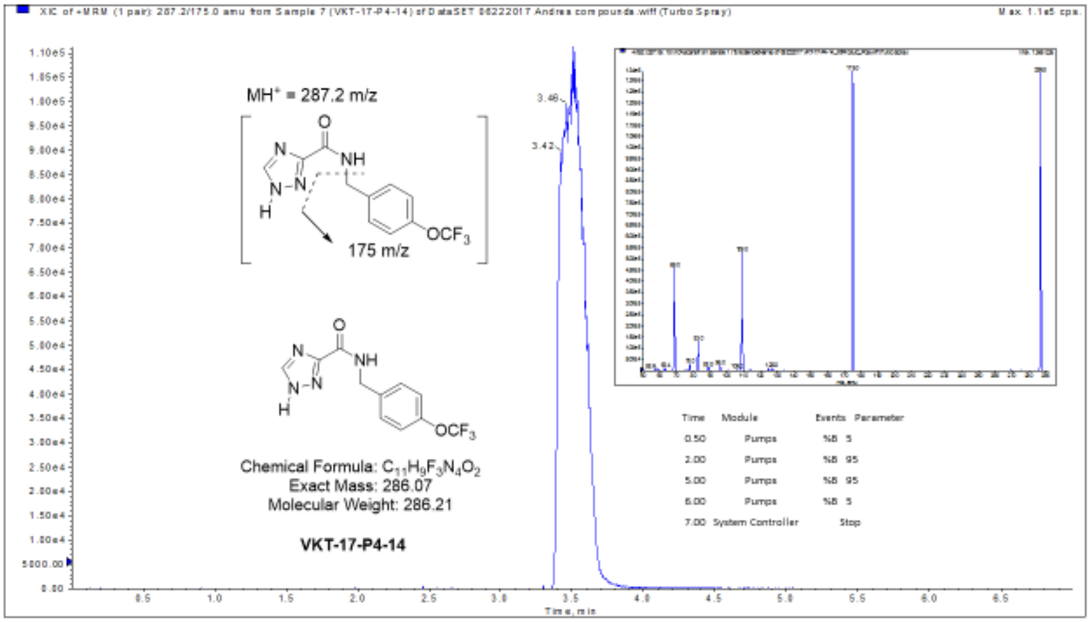

VKT-17-P4-15

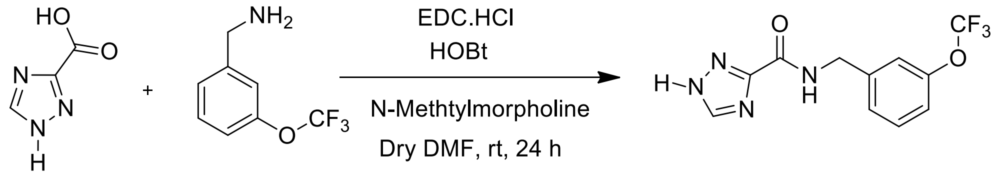

1 — CF: $C_3H_3N_3O_2$, M W: 113.07

18 — C F: $C_8H_8F_3NO$, M W: 191.15

19 — C F: $C_{11}H_{12}N_4O_2$, M W: 232.24

To a reaction of 1*H*-1,2,4-triazole-3-carboxylic acid (**1,** 113 mg, 1.19 mmol) and 3-(trifluoromethoxy)benzylamine (**18**, 250 mg, 1.31 mmol) in dry DMF (10 mL) was added 1-hydroxybenzotriazole monohydrate (200 mg, 1.31 mmol) and EDC (N-(3-Dimethylaminopropyl)-N′-ethylcarbodiimide) (251 mg, 1.31 mmol) followed by N-methylmorpholine (0.52 mL, 4.76 mmol via syringe). The mixture was stirred at room temperature under nitrogen and the solids were gradually dissolved. The contents were stirred at room temperature for 24.0 h, and then slowly diluted into iced water and extracted with DCM (2 × 50 mL). The DCM phase was washed with ice cold water (2 × 100 mL). The DCM phase was dried over anhydrous Na_2SO_4, filtered and concentrated under reduced pressure and chromatographed on silica gel using DCM and EtOAc (10:90) as eluents to get the desired amide **19** (**VKT-17-P4-15**, 108.7 mg, 32 % yield) as a white solid compound. ^{1}H-NMR (400 MHz, DMSO-*d*$_6$): δ14.67 (br s, 1H), 9.28 (br s, 1H), 8.53 (br s, 1H), 7.48–7.44 (m, 1H), 7.36–7.34 (m, 1H), 7.30 (br s, 1H), 7.24–7.22 (m, 1H), 4.50 (d, *J* = 6.4 Hz, 2H); ^{19}F-NMR (376 MHz, DMSO-*d*$_6$): δ –56.70 ppm. ESI(+) MH$^+$ = 287→ 175 *m/z*; t_R = 3.5 min.

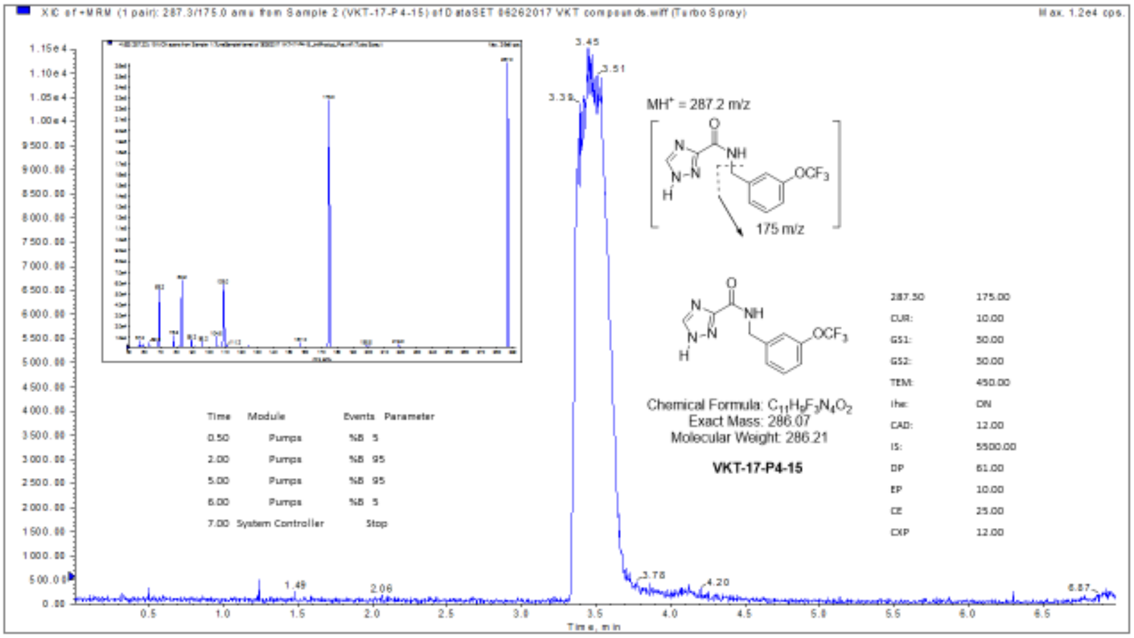

VKT-17-P4-16

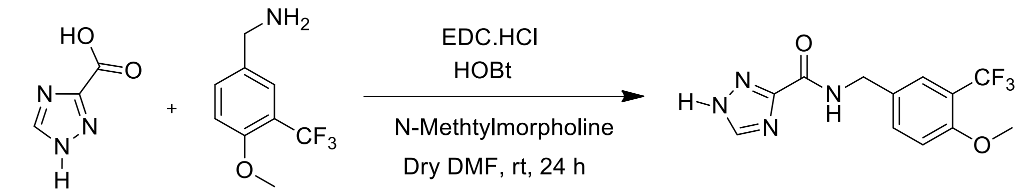

1 CF: $C_3H_3N_3O_2$ M W: 113.07

20 C F: $C_9H_{10}F_3NO$ M W: 205.18

21 C F: $C_{12}H_{11}F_3N_4O_2$ M W: 300.24

To a reaction of 1*H*-1,2,4-triazole-3-carboxylic acid (**1,** 100 mg, 0.87 mmol) and 4-Methoxy-3-(trifluoromethyl)benzylamine (**20**, 200 mg, 0.97 mmol) in dry DMF (10 mL) was added 1-hydroxybenzotriazole monohydrate (146 mg, 0.97 mmol) and EDC (N-(3-Dimethylaminopropyl)-N′-ethylcarbodiimide) (186 mg, 0.97 mmol) followed by N-methylmorpholine (0.38 mL, 3.48 mmol via syringe). The mixture was stirred at room temperature under nitrogen and the solids were gradually dissolved. The contents were stirred at room temperature for 24.0 h, and then slowly diluted into iced water and extracted with DCM (2 × 50 mL). The DCM phase was washed with ice cold water (2 × 100 mL). The DCM phase was dried over anhydrous Na_2SO_4, filtered and concentrated under reduced pressure and chromatographed on silica gel using EtOAc as eluents to get the desired amide **21** (**VKT-17-P4-16**, 62.8 mg, 24 % yield) as a white solid compound. ^{1}H-NMR (400 MHz, DMSO-*d*$_6$): δ14.66 (br s, 1H), 9.23 (br s, 1H), 8.49 (br s, 1H), 7.60–7.58 (m, 2H), 7.22 (d, *J* = 8.4 Hz, 1H), 4.43 (d, *J* = 6 Hz, 2H), 3.86 (s, 3H); ^{19}F-NMR (376 MHz, DMSO-*d*$_6$): δ –60.82 ppm. ESI(+) MH^+ = 301→ 189 *m/z*; t_R = 3.4 min.

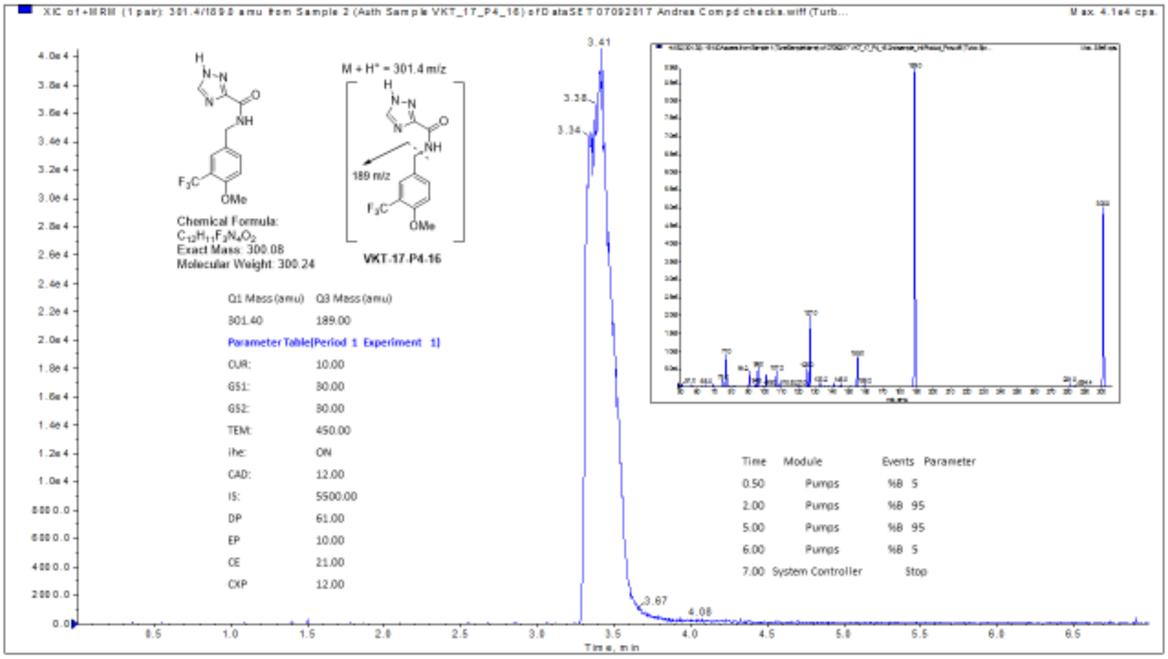

VKT-17-P4-17

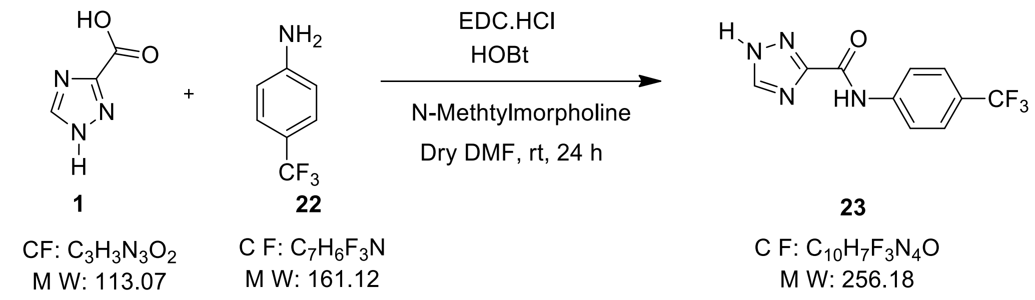

To a reaction of 1*H*-1,2,4-triazole-3-carboxylic acid (**1,** 226 mg, 2.0 mmol) and 4-(trifluoromethyl)aniline (**22**, 0.28 mL, 2.2 mmol) in dry DMF (10 mL) was added 1-hydroxybenzotriazole monohydrate (337 mg, 2.2 mmol) and EDC (N-(3-Dimethylaminopropyl)-N′-ethylcarbodiimide) (422 mg, 2.2 mmol) followed by N-methylmorpholine (0.88 mL, 8.0 mmol via syringe). The mixture was stirred at room temperature under nitrogen and the solids were gradually dissolved. The contents were stirred at room temperature for 24.0 h, and then slowly diluted into iced water and extracted with DCM (2 × 50 mL). The DCM phase was washed with ice cold water (2 × 100 mL). The DCM phase was dried over anhydrous Na_2SO_4, concentrated under reduced pressure and chromatographed on silica gel using EtOAc and Hexanes (80:20) as eluents to get the desired amide **23** (**VKT-17-P4-17**, 44.4 mg, 9 % yield) as a white solid compound. ^{1}H-NMR (400 MHz, DMSO-d_6): δ14.86 (br s, 1H), 10.90 (br s, 1H), 8.70 (br s, 1H), 8.12 (d, J = 8 Hz, 2H), 7.75 (d, J = 8 Hz, 2H); ^{19}F- NMR (376 MHz, DMSO-d_6): δ –60.46 ppm. ESI(+) MH^+ = 257→ 96 *m/z*; t_R = 3.5 min.

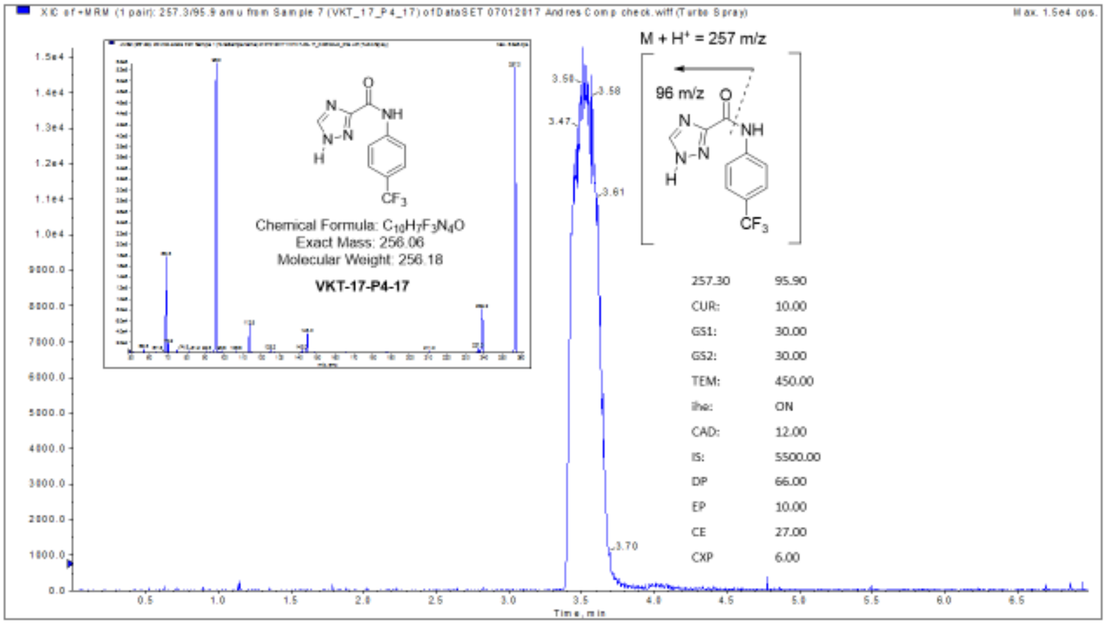

VKT-17-P4-18

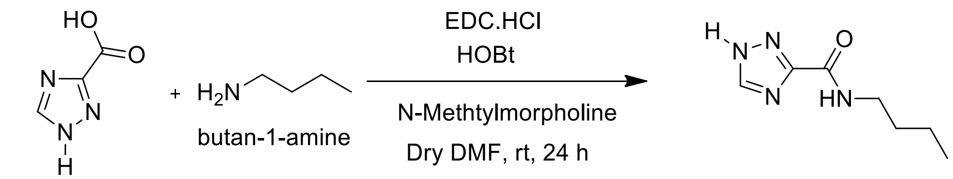

1	24	25
CF: $C_3H_3N_3O_2$<br>M W: 113.07	C F: $C_4H_{11}N$<br>M W: 73.14	C F: $C_7H_{12}N_4O$<br>M W: 168.20

To a reaction of 1*H*-1,2,4-triazole-3-carboxylic acid (**1,** 226 mg, 2.0 mmol) and butan-1-amine (**24**, 0.21 mL, 2.1 mmol) in dry DMF (10 mL) was added 1-hydroxybenzotriazole monohydrate (337 mg, 2.2 mmol) and EDC (N-(3-Dimethylaminopropyl)-N′-ethylcarbodiimide) (422 mg, 2.2 mmol) followed by N-methylmorpholine (0.88 mL, 8.0 mmol via syringe). The mixture was stirred at room temperature under nitrogen and the solids were gradually dissolved. The contents were stirred at room temperature for 24.0 h, and then slowly diluted into iced water and extracted with DCM (2 × 50 mL). The DCM phase was washed with ice cold water (2 × 100 mL). The DCM phase was dried over anhydrous Na_2SO_4, filtered and concentrated under reduced pressure and chromatographed on silica gel using EtOAc and Hexanes (80:20) as eluents to get the desired amide **25** (**VKT-17-P4-18**, 32 mg, 10 % yield) as a white solid compound. ^{1}H-NMR (400 MHz, DMSO-d_6): δ14.75–14.44 (m, 1H), 8.58 (br s, 1H), 8.41 (br s, 1H), 3.26–3.20 (m, 2H), 1.54–1.45 (m, 2H), 1.35–1.23 (m, 2H), 0.89 (t, J = 7.2 Hz, 3H) ppm. ESI(+) MH$^+$ = 169 → 96 *m/z*; t$_R$ = 3.1 min.

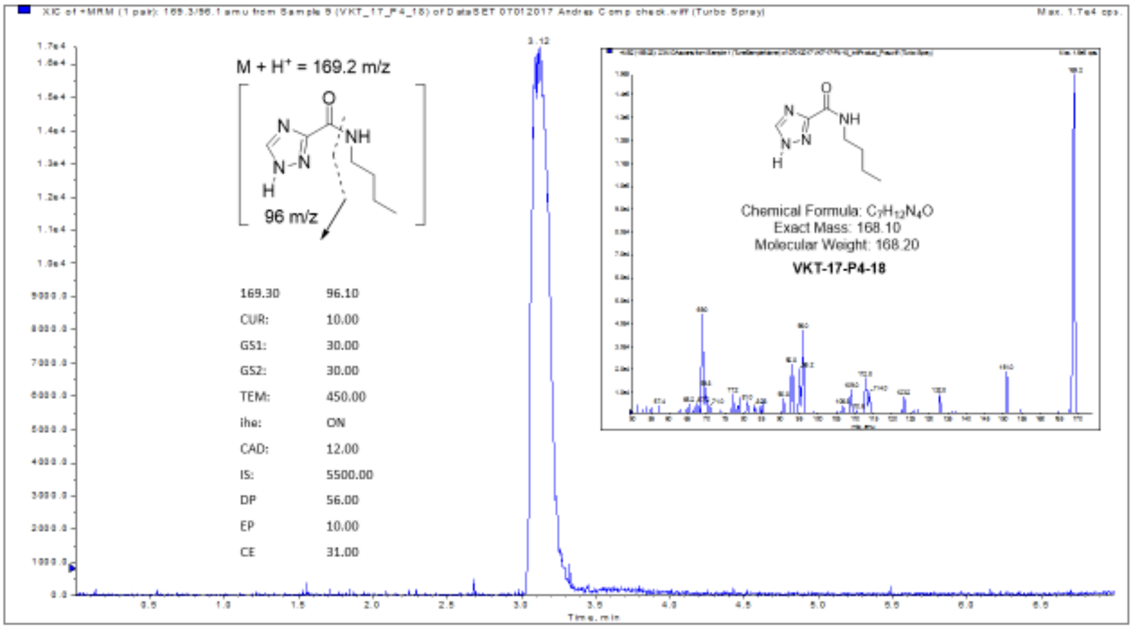

VKT-17-P4-19

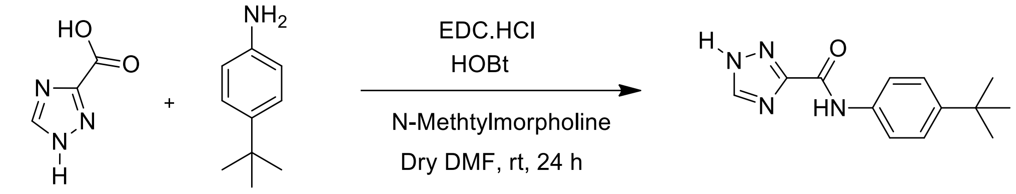

1 CF: $C_3H_3N_3O_2$ M W: 113.07

26 C F: $C_{10}H_{15}N$ M W: 149.23

27 C F: $C_{13}H_{16}N_4O$ M W: 244.29

To a reaction of 1*H*-1,2,4-triazole-3-carboxylic acid (**1,** 226 mg, 2.0 mmol) and 4-(tert-butyl)aniline (**26**, 0.33 mL, 2.1 mmol) in dry DMF (10 mL) was added 1-hydroxybenzotriazole monohydrate (337 mg, 2.2 mmol) and EDC (N-(3-Dimethylaminopropyl)-N′-ethylcarbodiimide) (422 mg, 2.2 mmol) followed by N-methylmorpholine (0.88 mL, 8.0 mmol via syringe). The mixture was stirred at room temperature under nitrogen and the solids were gradually dissolved. The contents were stirred at room temperature for 24.0 h, and then slowly diluted into iced water and extracted with DCM (2 × 50 mL). The DCM phase was washed with ice cold water (2 × 100 mL). The DCM phase was dried over anhydrous Na_2SO_4, filtered and concentrated under reduced pressure and chromatographed on silica gel using EtOAc and Hexanes (60:40) as eluents to get the desired amide **27** (**VKT-17-P4-19**, 123 mg, 25 % yield) as a white solid compound. ^{1}H-NMR (400 MHz, DMSO-d_6): δ14.84–14.72 (m, 1H), 10.42 (br s, 1H), 8.58 (br s, 1H), 7.74 (d, *J* = 8.8 Hz, 2H), 7.37 (d, *J* = 8.4 Hz, 2H), 1.28 (s, 9H) ppm. ESI(+) MH$^+$ = 245→ 96 *m/z*; t_R = 3.7 min.

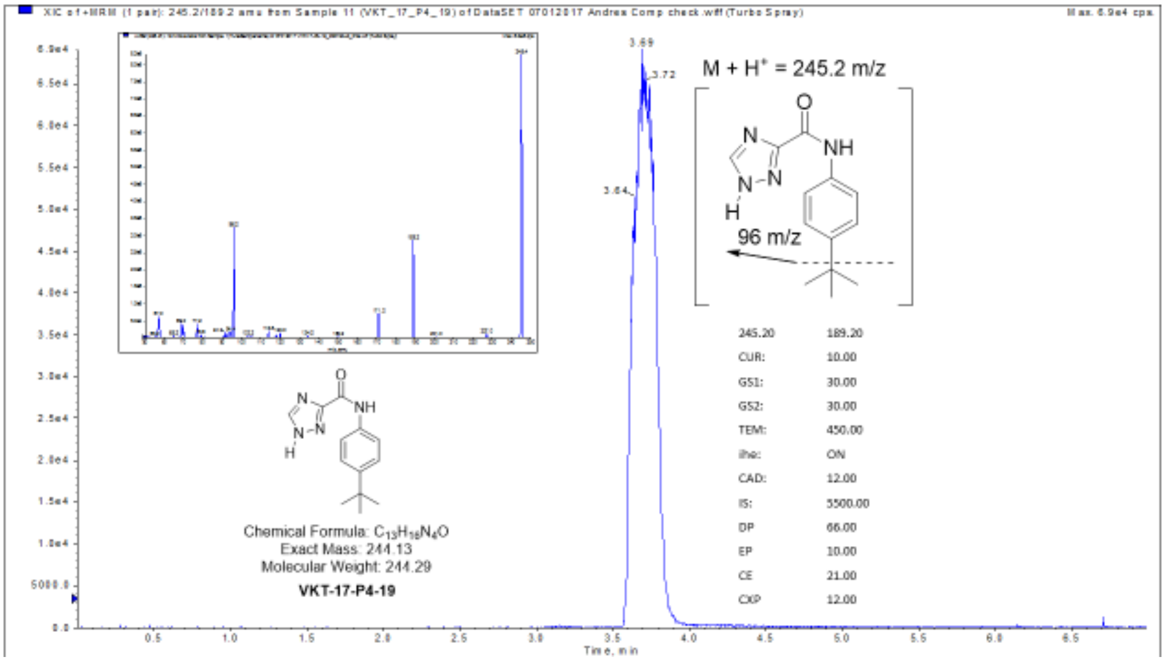

VKT-17-P4-21

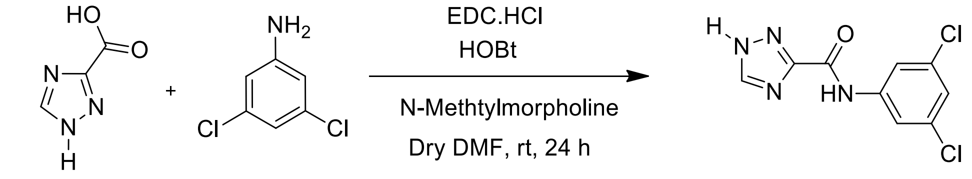

**1**	**28**	**29**
CF: $C_3H_3N_3O_2$	C F: $C_6H_5Cl_2N$	C F: $C_9H_6Cl_2N_4O$
M W: 113.07	M W: 162.02	M W: 257.08

To a reaction of 1*H*-1,2,4-triazole-3-carboxylic acid (**1,** 226 mg, 2.0 mmol) and 3,5-dichloroaniline (**28**, 340 mg, 2.1 mmol) in dry DMF (10 mL) was added 1-hydroxybenzotriazole monohydrate (337 mg, 2.2 mmol) and EDC (N-(3-Dimethylaminopropyl)-N′-ethylcarbodiimide) (422 mg, 2.2 mmol) followed by N-methylmorpholine (0.88 mL, 8.0 mmol via syringe). The mixture was stirred at room temperature under nitrogen and the solids were gradually dissolved. The contents were stirred at room temperature for 24.0 h, and then slowly diluted into iced water and extracted with DCM (2 × 50 mL). The DCM phase was washed with ice cold water (2 × 100 mL). The DCM phase was dried over anhydrous Na_2SO_4, filtered and concentrated under reduced pressure and chromatographed on silica gel using EtOAc and Hexanes (60:40) as eluents to get the desired amide **29** (**VKT-17-P4-21**, 68.6 mg, 13 % yield) as a white solid compound. ^{1}H-NMR (400 MHz, DMSO-d_6): δ14.85 (br s, 1H), 10.88 (br s, 1H), 8.69 (br s, 1H), 8.04–7.98 (m, 2H), 7.35–7.33 (m, 1H) ppm. ESI(+) MH^+ = 257→ 96 *m/z*; t_R = 3.7 min.

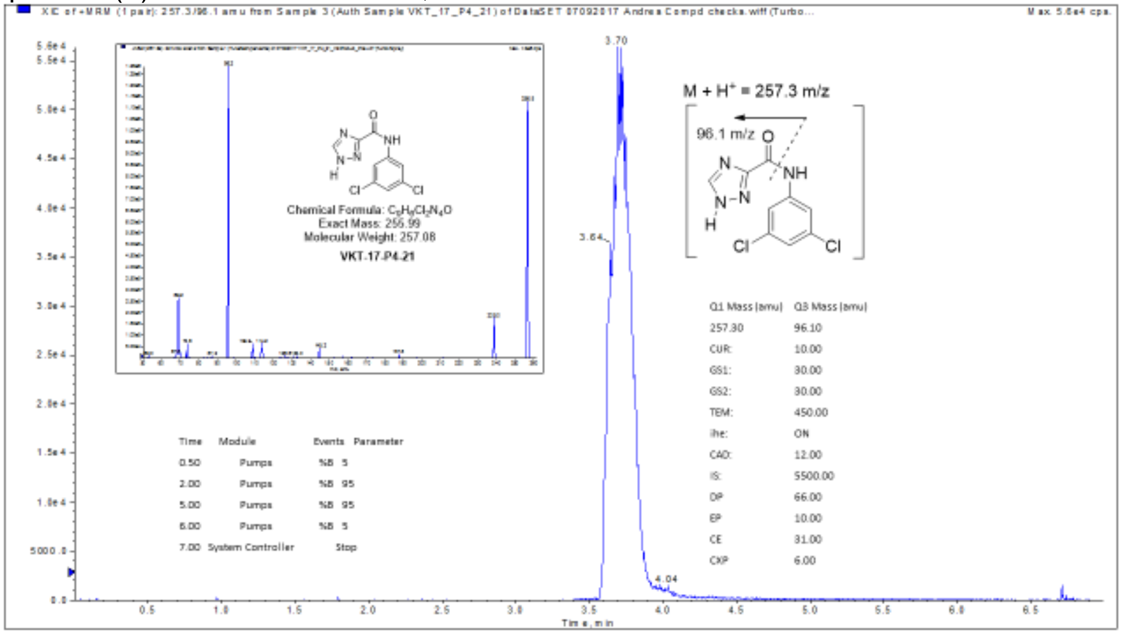

VKT-17-P4-22

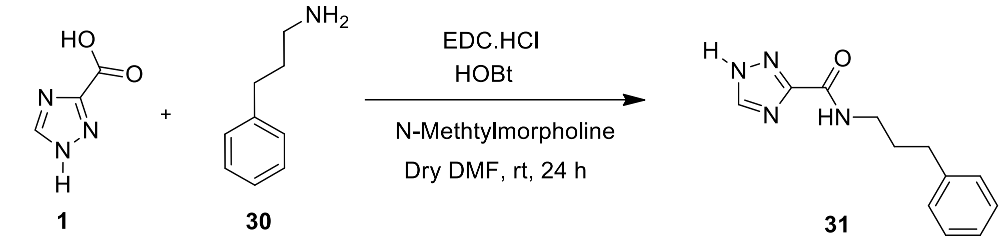

**1**	**30**	**31**
CF: $C_3H_3N_3O_2$	C F: $C_9H_{13}N$	C F: $C_{12}H_{14}N_4O$
M W: 113.07	M W: 135.21	M W: 230.27

To a reaction of 1*H*-1,2,4-triazole-3-carboxylic acid (**1,** 226 mg, 2.0 mmol) and 3-phenylpropan-1-amine (**30**, 0.30 mL, 2.1 mmol) in dry DMF (10 mL) was added 1-hydroxybenzotriazole monohydrate (337 mg, 2.2 mmol) and EDC (N-(3-Dimethylaminopropyl)-N′-ethylcarbodiimide) (422 mg, 2.2 mmol) followed by N-methylmorpholine (0.88 mL, 8.0 mmol via syringe). The mixture was stirred at room temperature under nitrogen and the solids were gradually dissolved. The contents were stirred at room temperature for 24.0 h, and then slowly diluted into iced water and extracted with DCM (2 × 50 mL). The DCM phase was washed with ice cold water (2 × 100 mL). The DCM phase was dried over anhydrous Na_2SO_4, filtered and concentrated under reduced pressure and chromatographed on silica gel using EtOAc as eluents to get the desired amide **31** (**VKT-17-P4-22**, 69.7 mg, 15 % yield) as a white solid compound. ^{1}H-NMR (400 MHz, DMSO-d_6): δ14.63 (br s, 1H), 8.68 (br s, 1H), 8.42 (br s, 1H), 7.30–7.18 (m, 5H), 3.40–3.24 (m, 2H), 2.61 (t, J = 7.6 Hz, 2H), 1.87–1.78 (m, 2H) ppm. ESI(+) MH^+ = 231→ 91 *m/z*; t_R = 3.4 min.

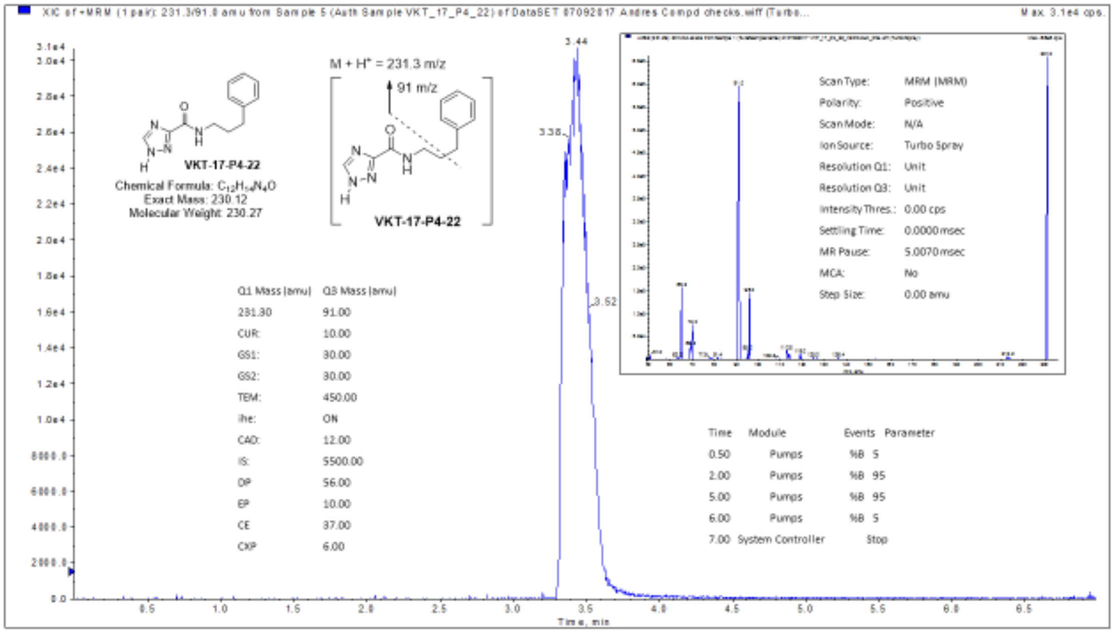

VKT-17-P4-23

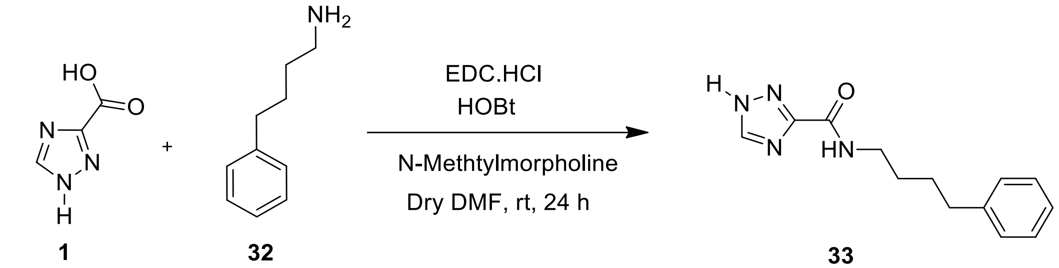

1 CF: $C_3H_3N_3O_2$ M W: 113.07

32 C F: $C_{10}H_{15}N$ M W: 149.23

33 C F: $C_{13}H_{16}N_4O$ M W: 244.29

To a reaction of 1*H*-1,2,4-triazole-3-carboxylic acid (**1,** 226 mg, 2.0 mmol) and 4-phenylbutan-1-amine (**32**, 0.33 mL, 2.1 mmol) in dry DMF (10 mL) was added 1-hydroxybenzotriazole monohydrate (337 mg, 2.2 mmol) and EDC (N-(3-Dimethylaminopropyl)-N′-ethylcarbodiimide) (422 mg, 2.2 mmol) followed by N-methylmorpholine (0.88 mL, 8.0 mmol via syringe). The mixture was stirred at room temperature under nitrogen and the solids were gradually dissolved. The contents were stirred at room temperature for 24.0 h, and then slowly diluted into iced water and extracted with DCM (2 × 50 mL). The DCM phase was washed with ice cold water (2 × 100 mL). The DCM phase was dried over anhydrous Na_2SO_4, filtered and concentrated under reduced pressure and chromatographed on silica gel using EtOAc and Hexanes (75:25) as eluents to get the desired amide **33** (**VKT-17-P4-23**, 129.1 mg, 25 % yield) as a white solid compound. ^{1}H-NMR (400 MHz, DMSO-d_6): δ14.64 (br s, 1H), 8.64 (br s, 1H), 8.42 (br s, 1H), 7.28–7.24 (m, 2H), 7.20–7.14 (m, 3H), 3.31–3.24 (m, 2H), 2.63–2.56 (m, 2H), 1.68–1.54 (m, 4H) ppm. ESI(+) MH$^+$ = 245→ 91 *m/z*; t_R = 3.5 min.

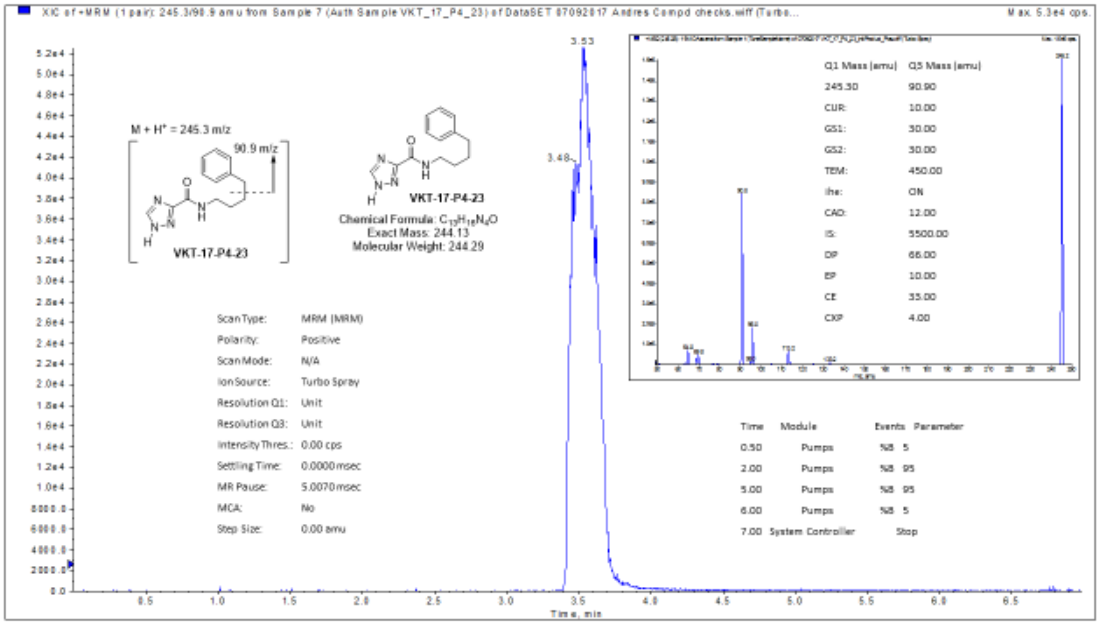

VKT-17-P4-24

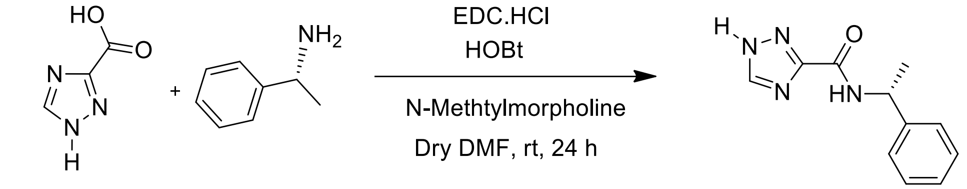

1 CF: $C_3H_3N_3O_2$ M W: 113.07

34 C F: $C_8H_{11}N$ M W: 121.18

35 C F: $C_{11}H_{12}N_4O$ M W: 216.24

To a reaction of 1*H*-1,2,4-triazole-3-carboxylic acid (**1,** 226 mg, 2.0 mmol) and (R)-(+)-a-methyl benzyl amine (**34**, 0.27 mL, 2.1 mmol) in dry DMF (10 mL) was added 1-hydroxybenzotriazole monohydrate (337 mg, 2.2 mmol) and EDC (N-(3-Dimethylaminopropyl)-N′-ethylcarbodiimide) (422 mg, 2.2 mmol) followed by N-methylmorpholine (0.88 mL, 8.0 mmol via syringe). The mixture was stirred at room temperature under nitrogen and the solids were gradually dissolved. The contents were stirred at room temperature for 24.0 h, and then slowly diluted into iced water and extracted with DCM (2 × 50 mL). The DCM phase was washed with ice cold water (2 × 100 mL). The DCM phase was dried over anhydrous Na_2SO_4, filtered and concentrated under reduced pressure and chromatographed on silica gel using EtOAc and Hexanes (75:25) as eluents to get the desired amide **35** (**VKT-17-P4-24,** 88 mg, 20 % yield) as a white solid compound. ^{1}H-NMR (400 MHz, DMSO-*d*$_6$): δ14.64 (br s, 1H), 8.97 (br s, 1H), 8.47 (br s, 1H), 7.42–7.40 (m, 2H), 7.34–7.30 (m, 2H), 7.25–7.21 (m, 1H), 5.19–5.12 (m, 1H), 1.50 (d, *J* = 6.8 Hz, 3H) ppm. ESI(+) MH^+ = 217→ 113 *m/z*; t_R = 3.3 min.

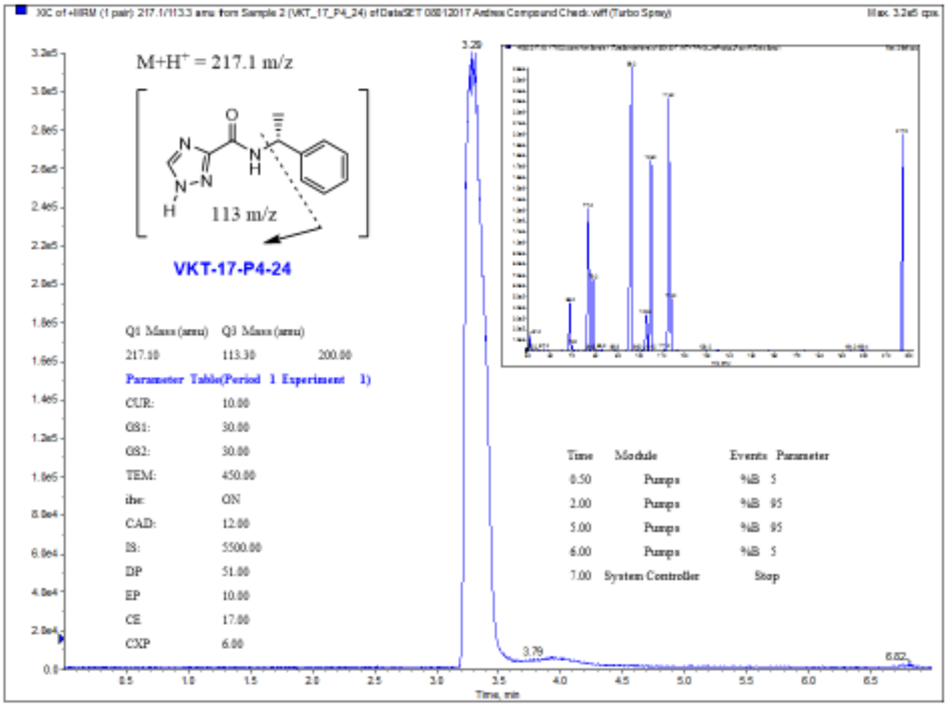

VKT-17-P4-25

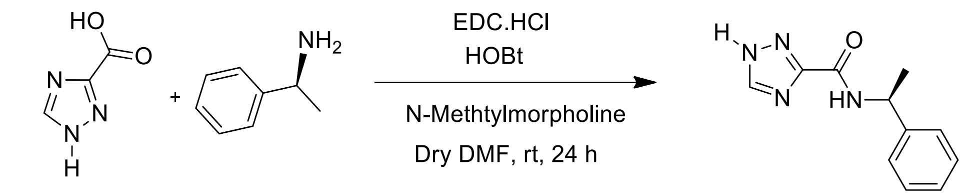

1	36	37
CF: $C_3H_3N_3O_2$	C F: $C_8H_{11}N$	C F: $C_{11}H_{12}N_4O$
M W: 113.07	M W: 121.18	M W: 216.24

To a reaction of 1*H*-1,2,4-triazole-3-carboxylic acid (**1,** 226 mg, 2.0 mmol) and (S)-(a)-methyl benzyl amine (**36**, 0.27 mL, 2.1 mmol) in dry DMF (10 mL) was added 1-hydroxybenzotriazole monohydrate (337 mg, 2.2 mmol) and EDC (N-(3-Dimethylaminopropyl)-N′-ethylcarbodiimide) (422 mg, 2.2 mmol) followed by N-methylmorpholine (0.88 mL, 8.0 mmol via syringe). The mixture was stirred at room temperature under nitrogen and the solids were gradually dissolved. The contents were stirred at room temperature for 24.0 h, and then slowly diluted into iced water and extracted with DCM (2 × 50 mL). The DCM phase was washed with ice cold water (2 × 100 mL). The DCM phase was dried over anhydrous Na_2SO_4, filtered and concentrated under reduced pressure and chromatographed on silica gel using EtOAc and Hexanes (75:25) as eluents to get the desired amide **37** (**VKT-17-P4-25**, 211 mg, 49 % yield) as a white solid compound. ^{1}H-NMR (400 MHz, DMSO-d_6): δ14.67 (br s, 1H), 8.97 (br s, 1H), 8.47 (br s, 1H), 7.42–7.40 (m, 2H), 7.32 (t, J = 7.4 Hz, 2H), 7.25–7.21 (m, 1H), 5.20–5.12 (m, 1H), 1.50 (d, J = 6.8 Hz, 3H,) ppm. ESI(+) MH$^+$ = 217→ 113 *m/z*; t_R = 3.3 min.

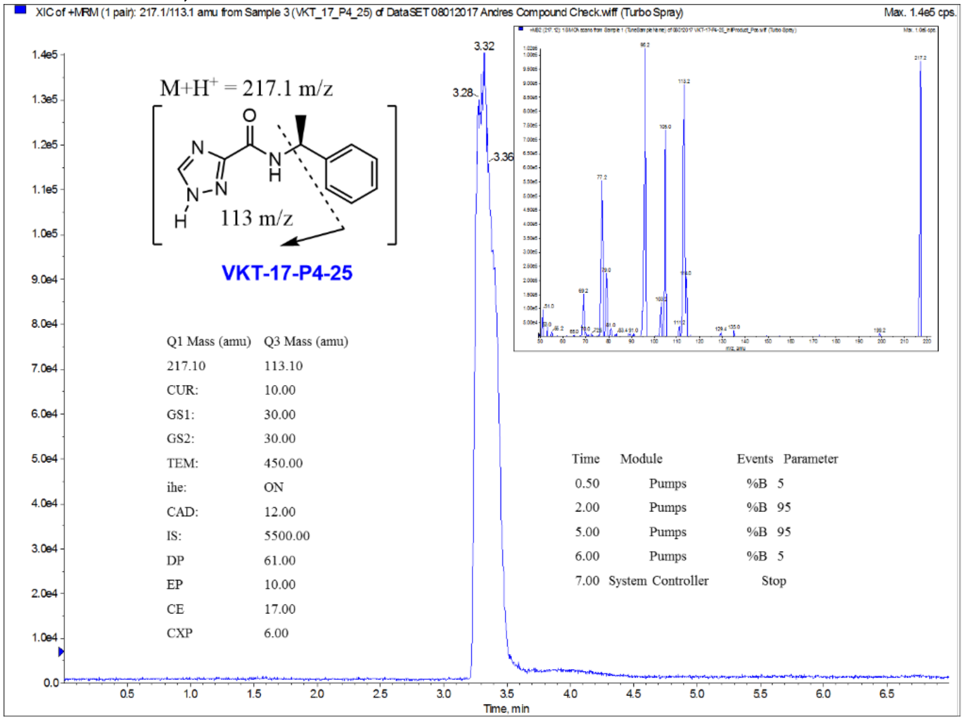

VKT-17-P4-27

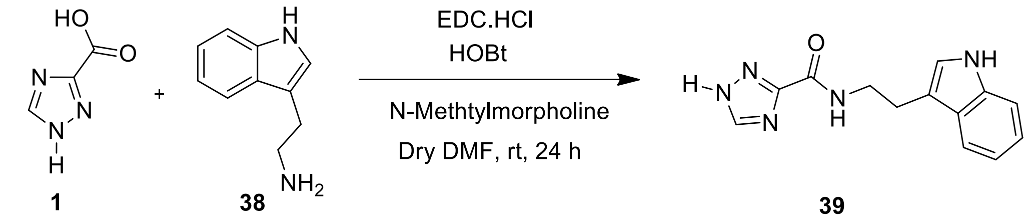

1 CF: $C_3H_3N_3O_2$ M W: 113.07

38 C F: $C_{10}H_{12}N_2$ M W: 160.22

39 C F: $C_{13}H_{13}N_5O$ M W: 255.28

To a reaction of 1*H*-1,2,4-triazole-3-carboxylic acid (**1,** 226 mg, 2.0 mmol) and 2-(1*H*-indol-3-yl)ethanamine (**38**, 0.34 mg, 2.1 mmol) in dry DMF (10 mL) was added 1-hydroxybenzotriazole monohydrate (337 mg, 2.2 mmol) and EDC (N-(3-Dimethylaminopropyl)-N′-ethylcarbodiimide) (422 mg, 2.2 mmol) followed by N-methylmorpholine (0.88 mL, 8.0 mmol via syringe). The mixture was stirred at room temperature under nitrogen and the solids were gradually dissolved. The contents were stirred at room temperature for 24.0 h, and then slowly diluted into iced water and extracted with DCM (2 × 50 mL). The DCM phase was washed with ice cold water (2 × 100 mL). The DCM phase was dried over anhydrous Na_2SO_4, filtered and concentrated under reduced pressure and chromatographed on silica gel using MeOH and DCM (10:90) as eluents to get the desired amide **39** (**VKT-17-P4-27**, 193.3 mg, 38 % yield) as a white solid compound. ^{1}H-NMR (400 MHz, DMSO-d_6): δ14.90–14.48 (m, 1H), 10.82 (s, 1H), 8.80–8.13 (m, 2H), 7.61 (d, J = 7.6 Hz, 1H), 7.35 (d, J = 8 Hz, 1H), 7.21–7.18 (m, 1H), 7.11–7.05 (m, 1H), 7.04–6.96 (m, 1H), 3.61–3.53 (m, 2H), 2.97 (t, J = 7.6 Hz, 2H) ppm. ESI(+) MH^+ = 256→ 144 *m/z*; t_R = 3.2 min.

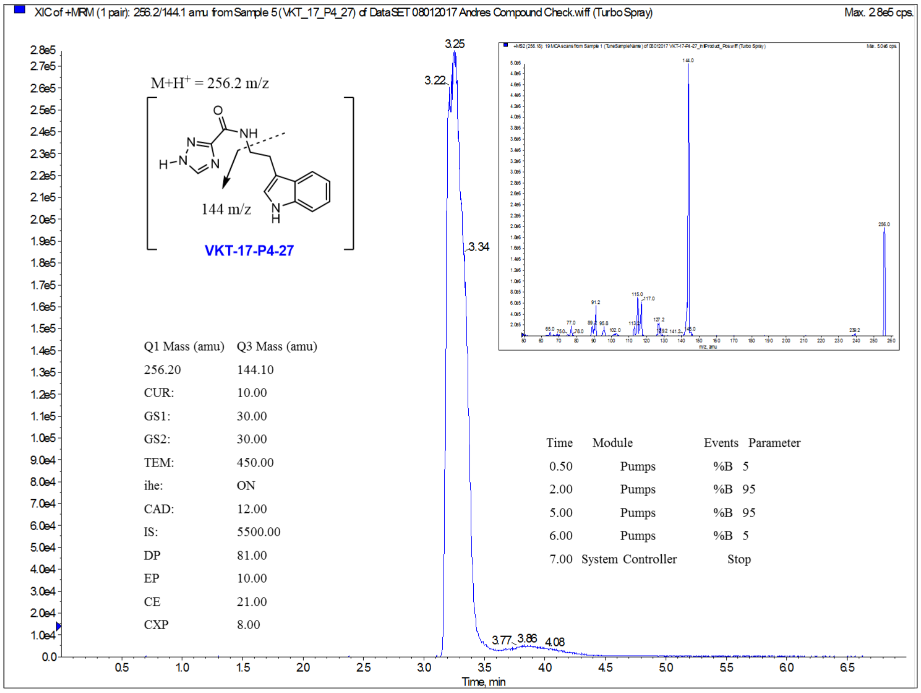

VKT-17-P4-28

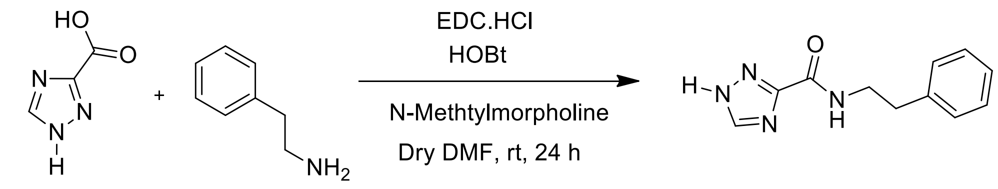

1: CF: $C_3H_3N_3O_2$, M W: 113.07

40: C F: $C_8H_{11}N$, M W: 121.18

41: C F: $C_{11}H_{12}N_4O$, M W: 216.24

To a reaction of 1*H*-1,2,4-triazole-3-carboxylic acid (**1,** 226 mg, 2.0 mmol) and 2-phenylethanamine (**40**, 0.27 mL, 2.1 mmol) in dry DMF (10 mL) was added 1-hydroxybenzotriazole monohydrate (337 mg, 2.2 mmol) and EDC (N-(3-Dimethylaminopropyl)-N′-ethylcarbodiimide) (422 mg, 2.2 mmol) followed by N-methylmorpholine (0.88 mL, 8.0 mmol via syringe). The mixture was stirred at room temperature under nitrogen and the solids were gradually dissolved. The contents were stirred at room temperature for 24.0 h, and then slowly diluted into iced water and extracted with DCM (2 × 50 mL). The DCM phase was washed with ice cold water (2 × 100 mL). The DCM phase was dried over anhydrous Na_2SO_4, filtered and concentrated under reduced pressure and chromatographed on silica gel using MeOH and DCM (5:95) as eluents to get the desired amide **41** (**VKT-17-P4-28**, 189.4 mg, 44 % yield) as a white solid compound. ^{1}H-NMR (400 MHz, DMSO-d_6): δ14.63–14.52 (m, 1H), 8.65–8.25 (m, 2H), 7.33–7.17 (m, 5H), 3.55–3.44 (m, 2H), 2.85 (t, J = 7.6 Hz, 2H,) ppm. ESI(+) MH^+ = 217→ 105 *m/z*; t_R = 3.3 min.

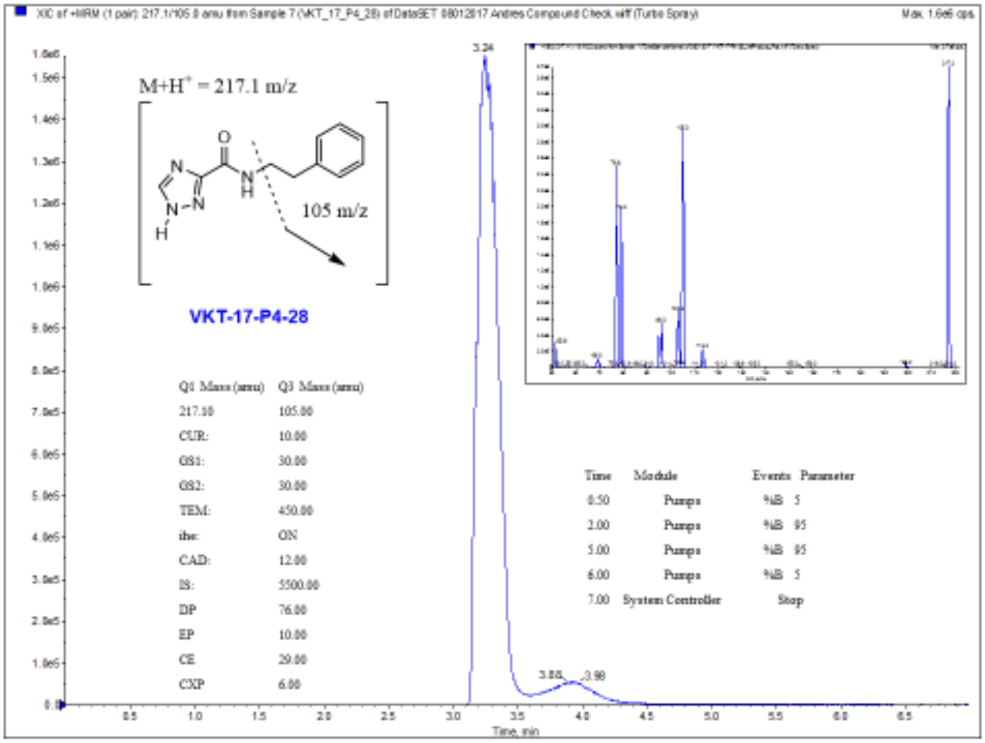

VKT-17-P4-29

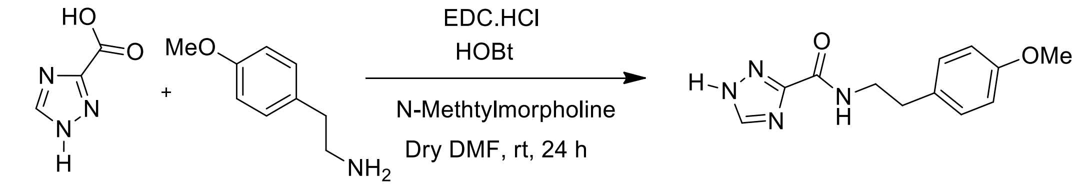

1

CF: $C_3H_3N_3O_2$
M W: 113.07

42

C F: $C_9H_{13}NO$
M W: 151.21

43

C F: $C_{12}H_{14}N_4O_2$
M W: 246.27

To a reaction of 1*H*-1,2,4-triazole-3-carboxylic acid (**1,** 226 mg, 2.0 mmol) and 2-(4-methoxyphenyl)ethanamine (**42**, 0.31 mL, 2.1 mmol) in dry DMF (10 mL) was added 1-hydroxybenzotriazole monohydrate (337 mg, 2.2 mmol) and EDC (N-(3-Dimethylaminopropyl)-N′-ethylcarbodiimide) (422 mg, 2.2 mmol) followed by N-methylmorpholine (0.88 mL, 8.0 mmol via syringe). The mixture was stirred at room temperature under nitrogen and the solids were gradually dissolved. The contents were stirred at room temperature for 24.0 h, and then slowly diluted into iced water and extracted with DCM (2 × 50 mL). The DCM phase was washed with ice cold water (2 × 100 mL). The DCM phase was dried over anhydrous Na_2SO_4, filtered and concentrated under reduced pressure and chromatographed on silica gel using MeOH and DCM (6:94) as eluents to get the desired amide **43** (**VKT-17-P4-29**, 193 mg, 39 % yield) as a white solid compound. ^{1}H-NMR (400 MHz, DMSO-d_6): δ14.76–14.35 (br s, 1H), 8.80–8.30 (m, 2H), 7.15 (d, J = 8.8 Hz, 2H), 6.85 (d, J = 8.8 Hz, 2H), 3.72 (s, 3H), 3.48–3.40 (m, 2H), 2.78 (t, J = 7.6 Hz, 2H) ppm. ESI(+) MH^+ = 247→ 135 m/z; t_R = 3.2 min.

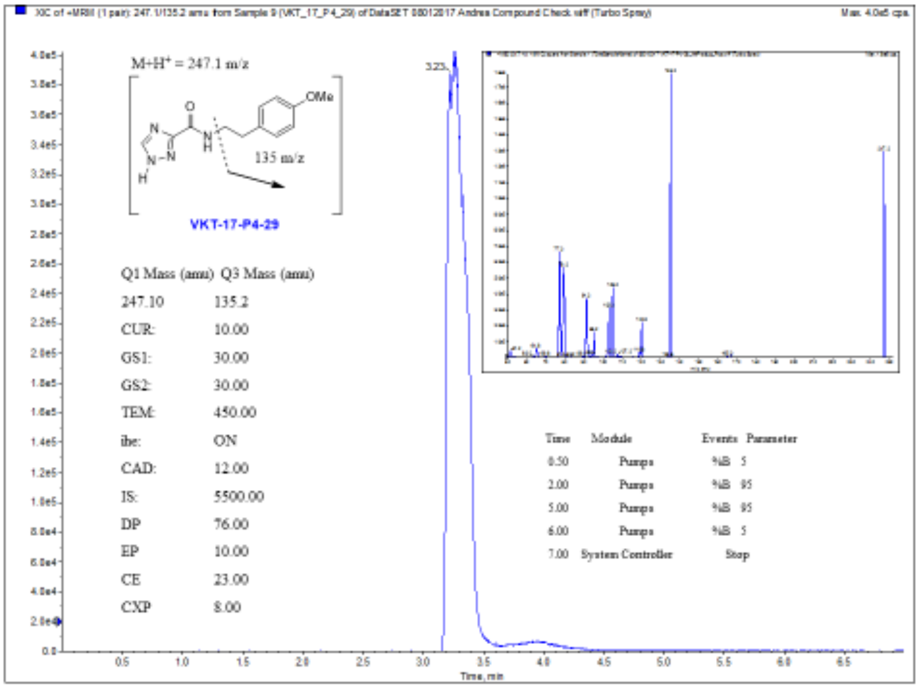

VKT-17-P4-32

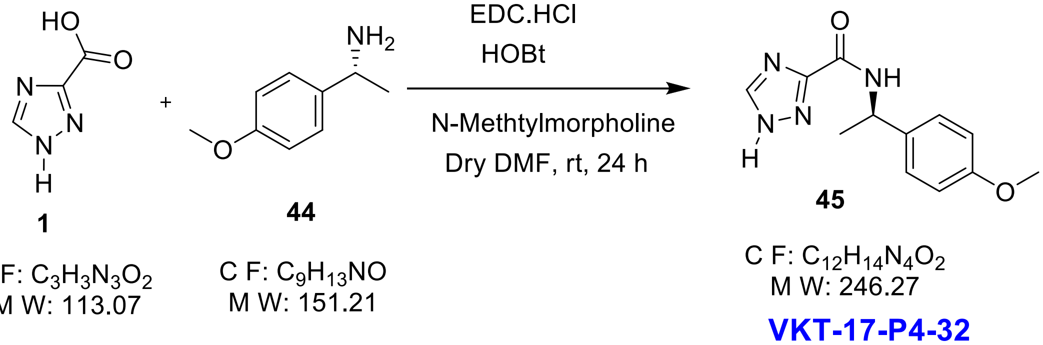

1 CF: $C_3H_3N_3O_2$ M W: 113.07

44 C F: $C_9H_{13}NO$ M W: 151.21

45 C F: $C_{12}H_{14}N_4O_2$ M W: 246.27 **VKT-17-P4-32**

To a reaction of 1*H*-1,2,4-triazole-3-carboxylic acid (**1,** 226 mg, 2.0 mmol) and (R)-(+)-4-Methoxy-α-methylbenzylamine (**10**, 0.28 mL, 2.1 mmol) in dry DMF (10 mL) was added 1-hydroxybenzotriazole monohydrate (337 mg, 2.2 mmol) and EDC (N-(3-Dimethylaminopropyl)-N′-ethylcarbodiimide) (422 mg, 2.2 mmol) followed by N-methylmorpholine (0.88 mL, 8.0 mmol via syringe). The mixture was stirred at room temperature under nitrogen and the solids were gradually dissolved. The contents were stirred at room temperature for 24 h, and then slowly diluted into iced water and extracted with DCM (50 mL × 2). The DCM phase was washed with ice cold water (100 mL x 2). The DCM phase was dried over anhydrous Na_2SO_4, filtered and concentrated under reduced pressure and chromatographed on silica gel using MeOH and DCM (5:95) as eluents to get the desired amide (**VKT-17-P4-32**, 287.1 mg, 58 % yield) as a white solid compound. ^{1}H-NMR (400 MHz, DMSO-d_6): δ14.98–14.44 (m, 1H), 9.16–8.08 (m, 2H), 7.34 (d, J = 8.4 Hz, 2H), 6.88 (d, J = 8.8 Hz, 2H,), 5.18–5.04 (m, 1H), 3.73 (s, 3H), 1.48 (d, J = 7.2 Hz, 3H,) ppm. ESI(+) MH^+ = 247→ 135 *m/z*; t_R = 3.3 min.

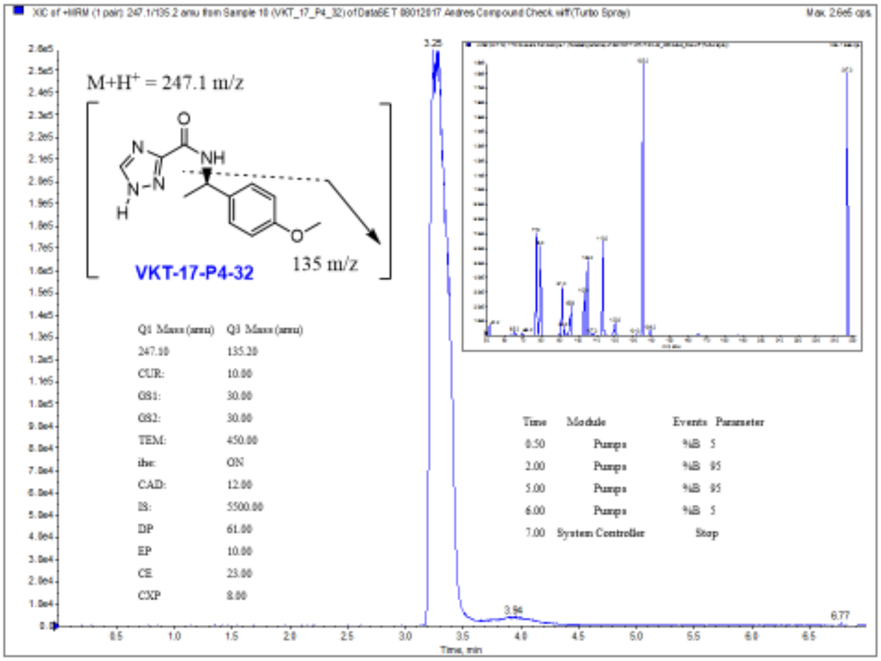

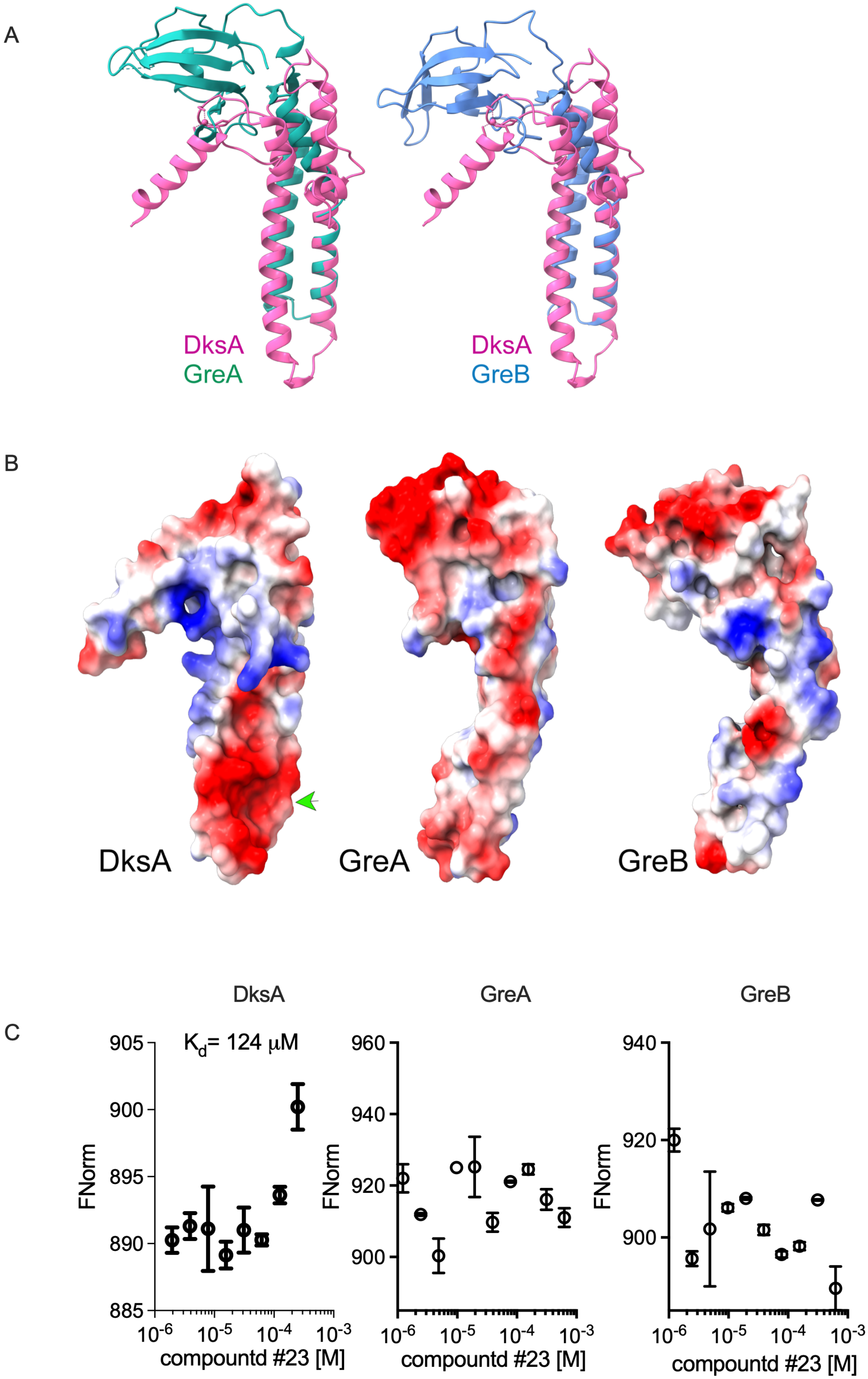

A
DksA
GreA
DksA
GreB
B
DksA
GreA
GreB
C
DksA
GreA
GreB
K_d= 124 μM
905
900
895
890
885
FNorm
10^{-6}
10^{-5}
10^{-4}
10^{-3}
compoundt #23 [M]
960
940
920
900
FNorm
compoundt #23 [M]
940
920
900
FNorm
compoundt #23 [M]

Figure S4. Binding of compound VKT-17-P4-23 to recombinant DksA, GreA and GreB proteins. (A) Superimposition of GreA (PDB 1GRJ), GreB (PDB 2P4V) and DksA (PDB 1TJL) protein structures. (B) Surface charge of factors that bind to the secondary channel of RNA polymerase. (C) Binding of compound VKT-17-P4-23 to recombinant DksA, GreA and GreB proteins was determined using microscale thermophoresis. Compound VKT-17-P4-23 serially diluted in PBS-Tween buffer was mixed with an equal volume of 20 nM NT647-labeled DksA, GreA and GreB proteins. The values are the mean ± SD from three independent experiments.

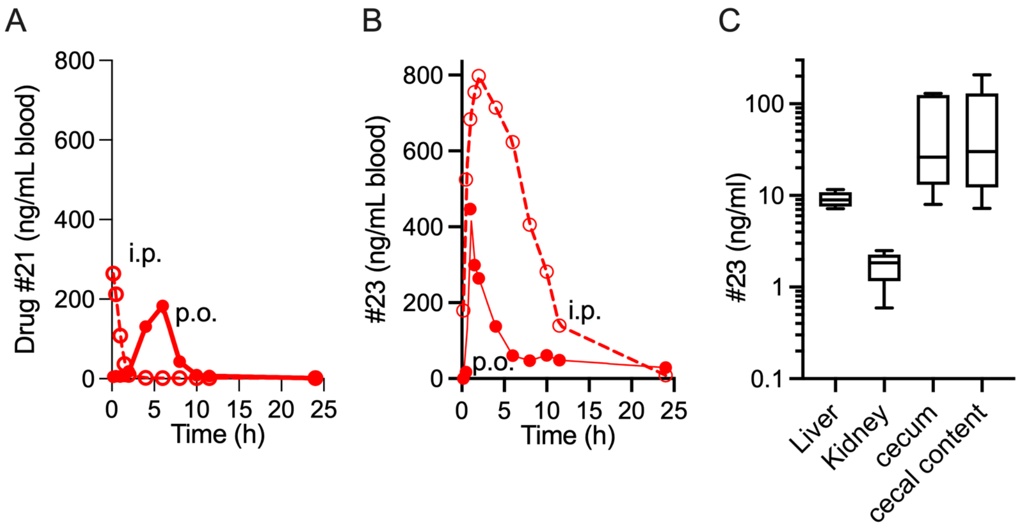

Figure S5. Pharmacokinetic and pharmacodynamic analyses. Rats were dosed i.v. at 1.0 mg/kg with (A) VKT-17-P4-21 or (B) VKT-17-P4-23. Concentration of the analogs were quantified by LC-MS and the data expressed as semi-log scale of blood concentration versus time data. (C) Concentration of VKT-17-P4-23 in the indicated organs of *Salmonella*-infected C57BL/6 mice dosed at 12 h intervals for 2 days. The organs were collected 2 days after i.p. infection with *Salmonella*.
